# Frequency and Distribution of Incidental Chromosomal Abnormalities Detected by Peripheral Blood Karyotyping: A Retrospective Study

**DOI:** 10.1155/genr/4906805

**Published:** 2026-05-17

**Authors:** Yaqin Zhang, Zongkai Bai, Rui Li, Han Yang, Jinfeng Wang, Yiying Chen

**Affiliations:** ^1^ Department of Reproductive Genetics, Pu’er People’s Hospital, Pu’er Hospital Affiliated to Kunming University of Science and Technology, Puer, China; ^2^ Department of Ophthalmology, Pu’er People’s Hospital, Pu’er Hospital Affiliated to Kunming University of Science and Technology, Puer, China

**Keywords:** clinical genetics, incidental chromosomal abnormalities, karyotype analysis, peripheral blood, retrospective study

## Abstract

**Objective:**

This study aims to examine the frequency and distribution of incidental chromosomal abnormalities identified through peripheral blood karyotype analysis and to evaluate their associations with sex, age, and chromosomal characteristics.

**Methods:**

This retrospective study conducted an analysis of 2397 peripheral blood karyotype assessments carried out between September 2023 and February 2025. The study employed conventional lymphocyte culture and G‐banding techniques, with karyotypes characterized in accordance with the International System for Human Cytogenomic Nomenclature (ISCN) 2024 guidelines. Chromosomal abnormalities that did not satisfy clinical reporting criteria were systematically documented. Detection rates were evaluated and compared across different sex and age groups, as well as chromosomal categories, utilizing chi‐square tests and linear trend analysis.

**Results:**

A total of 511 cases of incidental chromosomal abnormalities were identified, yielding an overall detection rate of 21.32%. No statistically significant difference was observed in the detection rates of abnormalities between females and males (*p* > 0.05). However, significant differences were noted among various age groups, with detection rates exhibiting an increasing trend with advancing age (*p* < 0.05). The distribution of abnormalities was uneven across chromosomes, predominantly concentrated in the C and D chromosome groups. Chromosomes 7, 9, and 14 were the most frequently affected. The most prevalent types of abnormalities included balanced translocations, deletions, and inversions, with t(7; 14) and del(9) (q13) being the most commonly observed. Additionally, 23 marker chromosomes were identified, displaying considerable morphological variability.

**Conclusion:**

Incidental chromosomal abnormalities identified through peripheral blood karyotype analysis exhibit nonrandom distributions associated with variables such as age and specific chromosomes. These low‐frequency occurrences may represent underlying somatic chromosomal variation and offer valuable reference data for the interpretation of karyotypes and the enhancement of laboratory quality control.

## 1. Introduction

Karyotype analysis constitutes a pivotal methodology in cytogenetic research, facilitating the systematic examination of chromosome number, morphology, and structure to identify various chromosomal abnormalities [1]. Numerous genetic disorders are intricately linked to numerical or structural chromosomal alterations [2], and even in the context of monogenic diseases, karyotype analysis can aid in the identification of potential chromosomal abnormalities [3]. Furthermore, tumor cells frequently exhibit numerical and structural chromosomal changes, and karyotype analysis of peripheral blood can yield critical insights for tumor diagnosis, classification, and prognostic evaluation [4, 5]. In the realm of prenatal screening, karyotype analysis of fetal cells obtained through amniocentesis in cases of advanced maternal age or within high‐risk populations can accurately detect trisomies or microdeletion syndromes [6].

The ongoing advancements in molecular genetic technologies have markedly improved the detection of small deletions, duplications, and complex genomic alterations through techniques such as fluorescence in situ hybridization (FISH) [7] and chromosomal microarray analysis (CMA) [8]. These methods offer significant advantages in terms of resolution and targeted analysis. However, they often depend on predefined probes or reference sequences, which can limit their capacity to comprehensively represent the overall chromosomal architecture and create challenges in identifying balanced translocations, inversions, and mosaicism. In contrast, conventional G‐banding karyotype [9] analysis provides direct visualization of chromosomal number and structural changes, thereby maintaining its status as the most fundamental and comprehensive method in both clinical and research contexts.

At present, karyotype analysis is advancing toward enhanced resolution, increased automation, and heightened precision. The integration of ultrahigh‐resolution microscopy, nanotechnology, and automated image analysis systems significantly improves the detection of low‐frequency or subtle chromosomal abnormalities [10–12]. These automated systems employ image recognition and computational algorithms to facilitate the automatic acquisition, analysis, and classification of chromosomal images, effectively minimizing human error and subjective bias and thereby enhancing reproducibility.

Despite rigorous compliance with standard operating procedures (SOPs), karyotype analysis presents several issues that require careful consideration. Low‐frequency incidental chromosomal abnormalities are often detected, yet they typically do not satisfy the criteria for reporting. The nature and significance of these abnormalities are not fully elucidated; they may indicate low‐level chromosomal instability events within the cell population or simply constitute background noise generated during culture or analysis [13]. A significant challenge in modern cytogenetic research is differentiating between biologically meaningful abnormalities and these incidentally observed variants.

In light of this, the current study systematically collected and analyzed 2397 peripheral blood karyotype results from a single institution over a specified timeframe. The study focused on reviewing and statistically analyzing incidental abnormalities that fell below established reporting standards. By examining distributional differences across variables such as sex, age, and chromosomal groups, the research sought to elucidate the overarching characteristics and potential patterns of incidental chromosomal abnormalities. The findings aim to furnish cytogenetic laboratory professionals with practical reference information pertinent to karyotype analysis, with a specific focus on the interpretation of incidental chromosomal abnormalities identified during routine examinations.

## 2. Materials and Methods

### 2.1. Source of Data

A total of 2397 peripheral blood karyotype analysis results were obtained from the Reproductive Genetics Laboratory of a hospital during the period from September 2023 to February 2025. The samples were collected from individuals undergoing genetic evaluation at the hospital, predominantly comprising adult patients seeking fertility‐related assessments and children or adolescents being evaluated for growth or developmental abnormalities.

Peripheral venous blood was drawn from all participants following a clinical assessment by physicians. The samples underwent routine lymphocyte culture followed by karyotype analysis. Basic clinical information for each sample was extracted from laboratory records, with all data anonymized prior to analysis to ensure the confidentiality of participants. This study utilized preexisting clinical data without the need for additional sample collection or interventional procedures, adhering to medical ethical standards, and received approval from the hospital’s ethics committee.

### 2.2. Karyotype Analysis Procedure

Peripheral blood karyotype analysis was meticulously performed in accordance with the laboratory’s SOPs. Two milliliters of peripheral venous blood was collected from each participant into heparinized tubes, and lymphocyte cultures were initiated within 24 h postcollection. The lymphocytes were cultured in a lymphocyte culture medium (Guangzhou Dahui Biotechnology Co., Ltd., China) using 0.5 mL of peripheral blood at 37°C for approximately 72 h. Colcemid was introduced to arrest cell division. Subsequently, the cells were subjected to a 0.075 mol/L KCl hypotonic solution for 30 min and fixed three times using a methanol–glacial acetic acid solution (3:1). The fixed cell suspension was then dispensed onto clean glass slides, which were baked at 80°C for 180 min. Subsequent to a brief digestion of the slides using an optimal concentration of trypsin solution, the enzymatic reaction was halted, and the slides were rinsed with phosphate‐buffered saline. This was followed by Giemsa staining to achieve distinct G‐banding patterns of the chromosomes.

Microscopic examination and image acquisition were conducted utilizing an Imager.Z2 microscope equipped with a digital imaging system (Zeiss, Germany). Initial analysis of chromosome images was carried out using the automated karyotyping system AutoVision V2.0.7.1 (Hangzhou Deshi Biotechnology Co., Ltd., China).

### 2.3. Karyotype Interpretation Criteria and Procedure

For each sample, a minimum of 30 metaphase cells were analyzed, with an expanded examination of 100 cells conducted in cases where mosaicism was suspected. Karyotypes were assigned and described in accordance with the ISCN 2024 guidelines. Structural abnormalities were deemed clonal if detected in two or more cells, while numerical abnormalities were classified as clonal when present in three or more cells. Although single‐cell abnormalities were documented, they were excluded from the formal clinical report. The analytical resolution achieved was approximately 400 bands. G‐banding analysis for each sample was independently conducted by two professionally trained cytogenetic analysts using a “dual‐review” workflow to ensure the accuracy and reproducibility of the findings. Upon completion of the karyotype report, all single‐cell abnormal karyotypes that did not satisfy the criteria for clinical reporting were reevaluated and incorporated into subsequent statistical analyses.

### 2.4. Statistical Analysis and Image Processing

All karyotype analysis results were meticulously verified and systematically entered into a comprehensive database. Statistical analyses were conducted on the entire cohort as well as pertinent subgroups utilizing SPSS software (Version 22.0, IBM Corp., USA). Initially, descriptive statistics were employed to summarize the detection rates of chromosomal abnormalities across various groups and to assess the distribution of these abnormalities among different subgroups. Chi‐square (*χ* ^2^) tests were utilized to compare detection rates of abnormalities across sexes, age groups, and chromosomal categories, with statistical significance set at *p* < 0.05. To facilitate a clear and intuitive presentation of the results, all statistical outputs and figures were standardized. Postprocessing and layout adjustments of abnormal karyotype images were executed using Inkscape software (Version 1.4.2) for labeling, formatting, and comparative display, ensuring that no substantive image content was altered.

## 3. Results

### 3.1. Sex Distribution

**FIGURE 1:**
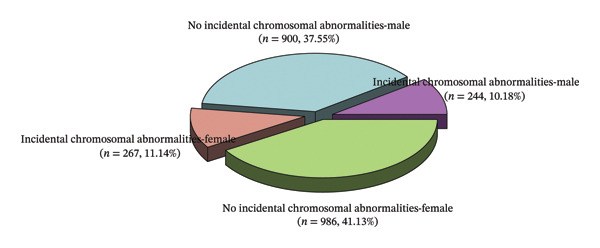
Sex distribution of samples in chromosomal karyotype analysis.

In this study, a total of 2397 valid samples were analyzed, consisting of 1144 male and 1253 female participants. Among the male cohort, 244 cases exhibited incidental abnormal karyotypes, whereas 900 cases were free of abnormalities. In the female cohort, 267 cases presented with incidental abnormal karyotypes, while 986 cases displayed no abnormalities. Statistical analysis revealed no significant difference between the sexes (*p* > 0.05) (Table 1 and Figure 1).

**TABLE 1:** Sex distribution of incidental chromosomal abnormalities [*n* (%)].

Category	Male (%)	Female (%)	*χ* ^2^	*p*
Incidental chromosomal abnormalities	244 (21.33)	267 (21.31)	0.000	0.991
No incidental chromosomal abnormalities	900 (78.67)	986 (78.69)		
Total	1144 (100.00)	1253 (100.00)		

### 3.2. Age Distribution of Incidentally Detected Chromosomal Abnormalities

**FIGURE 2:**
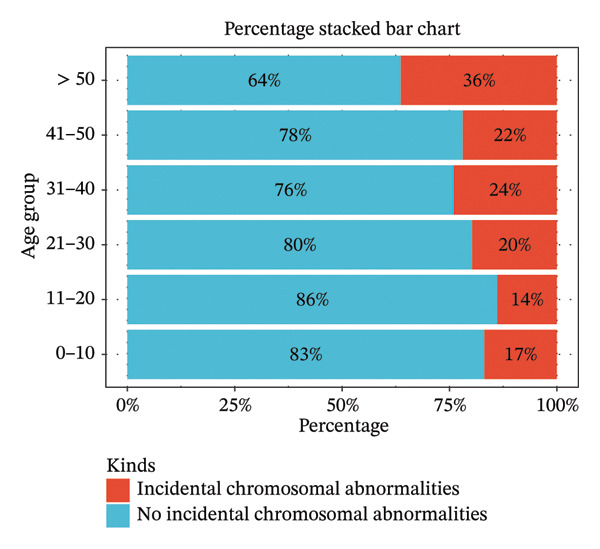
Proportion of incidentally detected chromosomal abnormalities across different age groups.

All 2397 samples were categorized into six age groups: 0–10, 11–20, 21–30, 31–40, 41–50, and > 50 years, with 319, 79, 759, 1051, 178, and 11 samples in each group, respectively. The numbers of incidentally detected chromosomal abnormalities in these groups were 54, 11, 150, 253, 39, and 4, corresponding to detection rates of 10.57%, 2.15%, 29.35%, 49.51%, 7.63%, and 0.78%. Chi‐square test indicated a statistically significant difference in detection rates among age groups (*χ* ^2^ = 13.610, *p* < 0.05). Further linear trend analysis revealed a significant linear relationship between age and the detection rate of incidental chromosomal abnormalities (linear trend *χ* ^2^ = 9.700, *p* < 0.05) (Table 2). The distribution of incidental chromosomal abnormalities across different age groups is shown in Figure 2.

**TABLE 2:** Age‐stratified distribution of incidental chromosomal abnormalities [*n* (%)].

Age (years)	Abnormal cases (%)	Total cases (%)	Abnormal rate (%)	*χ* ^2^	*p*	Linear trend *χ* ^2^	*p*
0–10	54 (10.57)	319 (13.31)	16.93	13.610	0.018	9.700	0.002
11–20	11 (2.15)	79 (3.30)	13.92				
21–30	150 (29.35)	759 (31.66)	19.76				
31–40	253 (49.51)	1051 (43.85)	24.07				
41–50	39 (7.63)	178 (7.43)	21.91				
> 50	4 (0.78)	11 (0.46)	36.36				
Total	511 (100.00)	2397 (100.00)	21.32				

### 3.3. Distribution of Incidentally Detected Chromosomal Abnormalities by Chromosome Groups (A–G, mar)

**FIGURE 3:**
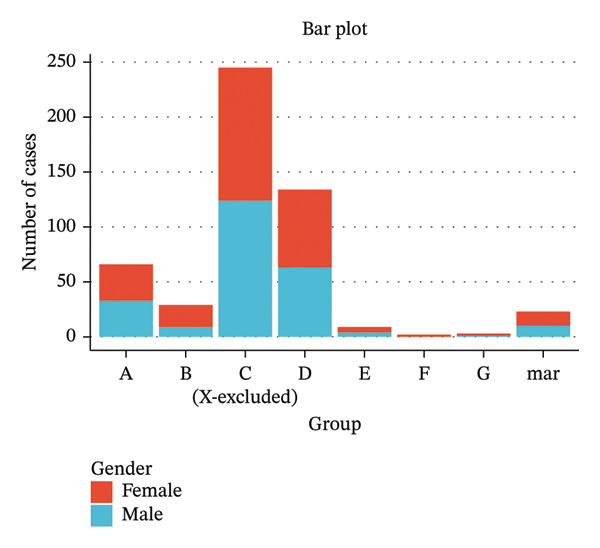
Sex distribution of incidentally detected chromosomal abnormalities across different chromosome groups.

To further investigate the distribution characteristics of incidentally detected chromosomal abnormalities, autosomes 1–22 were categorized into Groups A–G according to the International Chromosome Grouping System, while the X chromosome and marker chromosomes were analyzed separately. A total of 511 incidental abnormalities were identified. Among these, Group A chromosomes [1–3] comprised 66 cases (12.92%); Group B [4, 5] included 29 cases (5.68%); Group C [6–12] encompassed 245 cases (47.95%); Group D [13–15] accounted for 134 cases (26.22%); Group E [16–18] represented 9 cases (1.76%); Group F [19, 20] consisted of 2 cases (0.39%); and Group G [21, 22] included 3 cases (0.59%). A total of 23 marker chromosomes were identified, representing 4.50% of the sample. The prevalence of incidental abnormalities was notably higher in chromosomes belonging to the C and D groups, while it was comparatively lower in the F and G groups. Stratified analyses by sex and the overall population revealed significant variations in the detection rates of incidental chromosomal abnormalities across different chromosomal groups (*p* < 0.001) (Table 3). The distribution of incidental chromosomal abnormalities by sex across various chromosomal groups is illustrated in Figure 3.

**TABLE 3:** Grouped statistics of incidental chromosomal abnormalities [*n* (%)].

Chromosome groups	Male (%)	Female (%)	Total (%)
A	33 (6.46)	33 (6.46)	66 (12.92)
B	9 (1.76)	20 (3.91)	29 (5.68)
C (excluding X)	124 (24.27)	121 (23.68)	245 (47.95)
D	63 (12.33)	71 (13.89)	134 (26.22)
E	4 (0.78)	5 (0.98)	9 (1.76)
F	0 (0.00)	2 (0.39)	2 (0.39)
G	1 (0.20)	2 (0.39)	3 (0.59)
mar	10 (1.96)	13 (2.54)	23 (4.50)
*χ* ^2^	347.902	373.39	800.953
*p*	< 0.001	< 0.001	< 0.001

### 3.4. Distribution of Incidentally Detected Chromosomal Abnormalities by Chromosome Number

**FIGURE 4:**
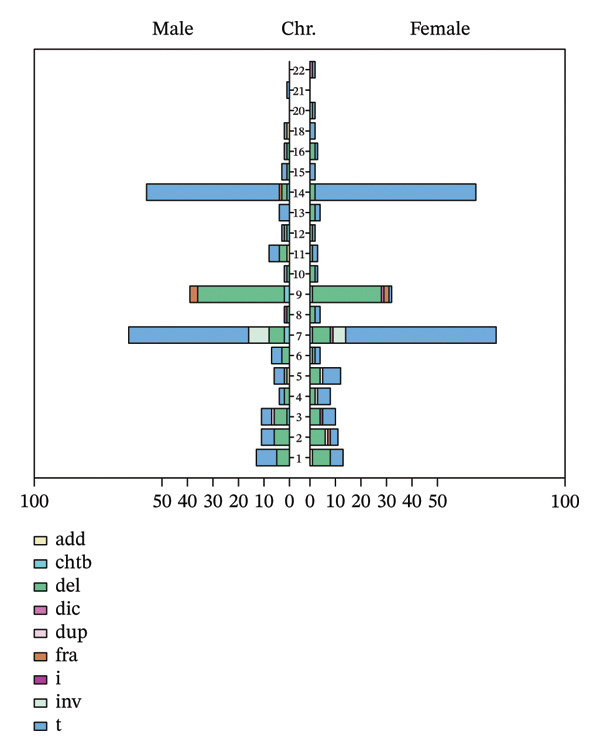
Distribution of karyotype types for incidentally detected abnormalities across chromosomes 1–22.

Following a comprehensive statistical analysis of incidental chromosomal abnormalities identified in all 2397 samples, it was determined that these abnormalities were distributed across multiple chromosomes, with notable variations in detection frequency among them. Among the autosomes, chromosomes 7, 9, and 14 exhibited the highest frequency of abnormalities, accounting for 26.61%, 13.89%, and 23.68% of all incidental abnormalities, respectively. A total of 23 marker chromosomes were identified, constituting 4.50% of the total abnormalities. These were characterized as supernumerary marker chromosomes of unknown origin, exhibiting variable fragment sizes. Chi‐square analysis revealed that the distribution of incidental chromosomal abnormalities across different chromosomes was statistically significant in males, females, and the overall population (*p* < 0.001) (refer to Table 4). The distribution of karyotype types of incidental abnormalities in chromosomes 1–22 is illustrated in Figure 4.

**TABLE 4:** Distribution of incidental chromosomal abnormalities [*n* (%)].

Chromosomes	Male (%)	Female (%)	Total (%)
1	13 (2.54)	13 (2.54)	26 (5.09)
2	11 (2.15)	11 (2.15)	22 (4.31)
3	9 (1.76)	9 (1.76)	18 (3.52)
4	3 (0.59)	8 (1.57)	11 (2.15)
5	6 (1.17)	12 (2.35)	18 (3.52)
6	7 (1.37)	4 (0.78)	11 (2.15)
7	63 (12.33)	73 (14.29)	136 (26.61)
8	2 (0.39)	4 (0.78)	6 (1.17)
9	39 (7.63)	32 (6.26)	71 (13.89)
10	2 (0.39)	3 (0.59)	5 (0.98)
11	8 (1.57)	3 (0.59)	11 (2.15)
12	3 (0.59)	2 (0.39)	5 (0.98)
13	4 (0.78)	4 (0.78)	8 (1.57)
14	56 (10.96)	65 (12.72)	121 (23.68)
15	3 (0.59)	2 (0.39)	5 (0.98)
16	2 (0.39)	3 (0.59)	5 (0.98)
18	2 (0.39)	2 (0.39)	4 (0.78)
20	0 (0.00)	2 (0.39)	2 (0.39)
21	1 (0.20)	0 (0.00)	1 (0.20)
22	0 (0.00)	2 (0.39)	2 (0.39)
mar	10 (1.96)	13 (2.54)	23 (4.50)
*χ* ^2^	480.648	588.506	1178.164
*p*	< 0.001	< 0.001	< 0.001

### 3.5. High‐Frequency Autosomal Incidentally Detected Abnormalities

**FIGURE 5:**
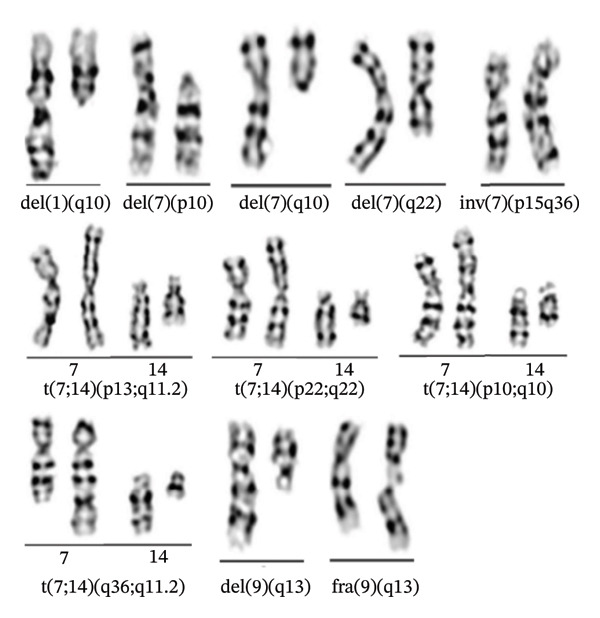
Representative karyotypes of high‐frequency autosomal incidental abnormalities.

All incidentally identified autosomal abnormalities within the study samples underwent systematic analysis. The findings revealed that chromosomes 7 and 9 exhibited the highest detection rates of abnormalities. Table 5 delineates the specific types and frequencies of prevalent high‐frequency incidental abnormalities, while Figure 5 provides representative karyotype images. These images depict the primary structural variants observed, including pericentromeric inversions, small fragment deletions, and balanced translocations.

**TABLE 5:** Representative high‐frequency incidental chromosomal abnormalities [*n* (%)].

No.	Chromosomes	Abnormal karyotypes	Number [*n* (%)]
1	1	del(1) (q10)	3 (0.13)
2	7	del(7) (p10)	6 (0.25)
3	7	del(7) (q10)	3 (0.13)
4	7	del(7) (q22)	4 (0.17)
5	7	inv(7) (p15q36)	10 (0.42)
6	7, 14	t(7; 14) (p13; q11.2)	36 (1.50)
7	7, 14	t(7; 14) (p22; q22)	4 (0.17)
8	7, 14	t(7; 14) (q10; q10)	9 (0.38)
9	7, 14	t(7; 14) (q36; q11.2)	45 (1.88)
10	9	del(9) (q13)	56 (2.34)
11	9	fra(9) (q13)	6 (0.25)

A comprehensive summary of all incidentally detected abnormalities for each chromosome is provided, accompanied by a complete set of images (refer to Appendix/Supporting Information Table S1 and Figures S1–S13).

### 3.6. Distribution of Marker Chromosome Abnormalities

**FIGURE 6:**
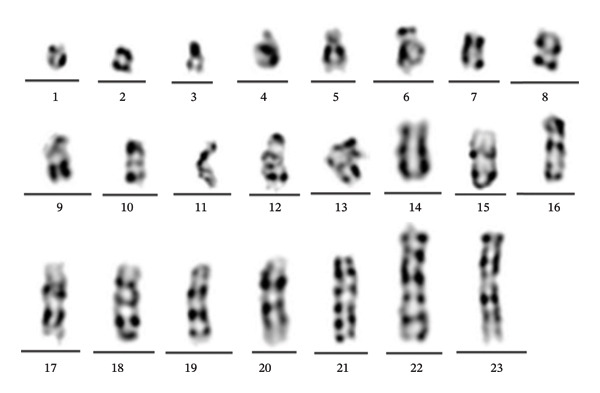
Karyotype images of marker chromosomes.

All identified marker chromosomes were systematically cataloged and analyzed. A cohort of 23 individuals was found to possess marker chromosomes, with representative images provided in Figure 6. These marker chromosomes demonstrated significant variation in size, morphology, and banding patterns, effectively highlighting the diversity and complexity of incidentally detected abnormalities associated with marker chromosomes.

## 4. Discussion

This study conducted a systematic analysis of 2397 peripheral blood karyotype results, concentrating on the patterns of occurrence of incidental chromosomal abnormalities. The findings suggest that such incidental abnormalities are not uncommon in the general population. Although their detection rate is lower than that of clinically apparent chromosomal anomalies, they are observed in individuals undergoing fertility evaluations and those with growth and developmental abnormalities. Additionally, incidental chromosomal abnormalities demonstrate significant variation across sex, age, and chromosome type, with multiple recurrent autosomal abnormalities and marker chromosomes identified. These results enhance our understanding of somatic chromosomal structural variability in peripheral blood and provide foundational data for evaluating chromosomal stability at the population level. The systematic cataloging and distribution analysis of these low‐frequency abnormalities aid in distinguishing background laboratory noise from potentially genuine variants, thereby offering a basis for further exploration of the spectrum of human chromosomal variation.

A more academic revision of the sentence could be: An in‐depth examination of incidental chromosomal abnormalities uncovers their varied and intricate origins. Certain abnormalities may result from technical artifacts or pseudoaberrations encountered during cell culture processes; for instance, in samples with a low mitotic index, incomplete cell synchronization or suboptimal chromosome spreading can lead to apparent chromosome breaks or uneven stretching [14]. Minor variations in the culture conditions of peripheral blood lymphocytes, such as culture duration, mitogen concentration, or banding quality, may also induce nonspecific structural alterations [15]. However, even after verification by two analysts and strict compliance with SOPs, a significant proportion of these abnormalities demonstrate reproducibility and patterned recurrence, manifesting at specific chromosomal regions or banding intervals at frequencies surpassing random background levels. This observation suggests that these abnormalities are not merely technical artifacts but may indicate somatic genetic instability or early clonal expansion. While these variants do not fulfill the criteria for formal reporting, their frequency and distribution continue to be of significant research interest. These incidental variants may indicate subtle fluctuations in an individual’s somatic genetic background or represent the “latent phase” of early clonal aberrations. Conducting systematic identification and statistical analysis of these abnormalities within large‐scale karyotype datasets can enhance laboratory quality control and yield valuable insights into chromosomal stability, population genetic diversity, and subclinical somatic genomic variation.

This study conducted a comparative analysis of the sex distribution of incidental chromosomal abnormalities identified through peripheral blood karyotype analysis. The findings indicated no statistically significant difference in the detection rates of abnormalities between male and female subjects. This outcome suggests that the prevalence of incidental chromosomal abnormalities in the general population under investigation does not demonstrate a pronounced sex bias. From a genetic perspective, most chromosomal structural abnormalities predominantly result from errors in chromosome segregation during meiosis or early embryonic cell divisions [16–18]. Theoretically, these errors are not intrinsically associated with the individual’s sex. It is important to note that the incidental abnormalities examined in this study are likely somatic chromosomal changes, which are more closely related to stochastic chromosomal instability events during cell proliferation rather than fixed hereditary mutations. Consequently, there is no evident biological correlation with the sex of the individual. These results further corroborate the hypothesis that incidental chromosomal abnormalities in the general population primarily reflect random cytogenetic events rather than sex‐related genetic susceptibility.

This study identified significant variations in the detection rates of incidental chromosomal abnormalities across different age groups, with a notable upward trend correlating with advancing age. These findings imply that age is a critical factor influencing chromosomal stability in peripheral blood cells. At the cytogenetic level, aging is associated with the accumulation of DNA damage in somatic cells due to environmental factors such as oxidative stress, radiation, and chemical exposures. Concurrently, the efficiency of DNA repair mechanisms diminishes, and cell cycle regulatory processes weaken, thereby increasing the likelihood of chromosomal breaks, aneuploidy, and structural abnormalities [19–21]. Furthermore, the clonal expansion of hematopoietic stem cell populations during aging [22] may contribute to genetic heterogeneity within peripheral blood lymphocyte populations, thereby enhancing the detection rate of incidental chromosomal abnormalities. Comparable patterns have been documented in the academic literature, with numerous recent large‐scale genomic and cytogenetic investigations suggesting that sporadic or low‐frequency somatic structural or sequence variants—such as mosaic chromosomal alterations and other nonclonal chromosomal abnormalities—exhibit a significant increase with age in the peripheral blood of healthy adults, indicative of somatic genetic drift [23–25]. As individuals advance in age, the cumulative likelihood of random errors occurring during chromosomal replication and division escalates. Although many of these abnormalities do not exhibit clonal characteristics, they nonetheless serve as markers of progressively heightened genomic instability [26]. While these low‐frequency incidental abnormalities are predominantly nonpathogenic, they may function as a “background signal” of disease susceptibility under certain conditions, particularly in older populations, and could be potentially linked to an increased risk of hematologic disorders, immune senescence, and tumor development [19, 27, 28].

From a detection standpoint, age‐associated alterations in cellular activity and culture characteristics may indirectly impact karyotype outcomes. Peripheral blood lymphocytes from older individuals frequently demonstrate diminished responsiveness to mitotic stimulants, leading to a reduced number of metaphase spreads and potentially lower banding quality [29]. This may elevate the probability of sporadic structural abnormalities or banding artifacts. Nevertheless, in this study, the implementation of standardized SOPs alongside a double‐review system effectively mitigated technical artifacts. This suggests that the observed age‐related trends are more likely indicative of authentic biological differences rather than detection errors.

This study identified that among incidentally detected karyotype abnormalities, chromosomes 7, 9, and 14 demonstrated relatively higher detection rates. Group‐based analysis further revealed that abnormalities in Groups C and D occurred with significantly greater frequency compared to other groups. These findings imply that the occurrence of incidental chromosomal abnormalities is not random but may exhibit specific predilections toward certain chromosomes.

Structurally, chromosomes 7, 9, and 14 are metacentric or near‐metacentric, characterized by banding regions enriched in heterochromatic segments, notably the qh region of chromosome 9 and the pericentromeric region of chromosome 14. These regions are frequently considered fragile sites, susceptible to nonspecific stretching, breaks, or centromeric rearrangements during chromosome segregation, replication, or banding, potentially manifesting as sporadic or nonclonal abnormalities [30, 31]. Furthermore, chromosome 7 has been reported to contain a relatively high proportion of incidental structural variations across various cell lines and peripheral blood samples, possibly attributable to its substantial content of repetitive sequences and active recombination hotspots [32].

In this dataset, the chromosomal abnormalities t(7; 14) (p13; q11.2), t(7; 14) (q36; q11.2), and del(9) (q13) were identified as commonly occurring incidental autosomal abnormalities. The 14q11.2 region contains essential T‐cell receptor loci (TCRA/D) in lymphocytes and is subject to somatic V(D)J recombination, which predisposes it to breaks and translocations in peripheral blood cells [33]. Chromosomal disruptions involving 14q11.2 significantly increase the probability of structural rearrangements linked to immune cell‐specific recombination, including translocations with other chromosomes. This phenomenon accounts for the relatively high incidence of t(7; 14) translocations involving 14q11.2 observed in peripheral blood samples. In considering 7p13 and 7q36 as breakpoint regions on chromosome 7, prior research has demonstrated that multiple bands on chromosome 7, including 7p13, are enriched with tandem repeat sequences. Additionally, certain fragile regions on chromosome 7, such as FRA7H, display replication asynchrony and a propensity for breakage [34, 35]. Moreover, investigations involving male carriers of chromosome 7 inversions have identified 7q36 as a recurrent breakpoint, indicating structural instability at this locus [36]. Regarding the del(9) (q13) breakpoint, it is situated near the pericentromeric/heterochromatic region of chromosome 9. The proximal long arm of 9q is characterized by an abundance of satellite sequences and heterochromatin, which exhibit significant variability in banding patterns and are susceptible to contraction, stretching, or pseudobreakage during slide preparation and banding processes [30, 37].

During the data compilation phase of this study, certain samples exhibited gains or losses of the X chromosome. It is important to acknowledge that, in routine karyotype analysis of female peripheral blood, the occurrence of X chromosome gain or loss in a limited number of metaphases is relatively common and is generally attributed to physiological or culture‐related phenomena. Such variations are typically not considered to have definitive clinical significance. Consequently, these alterations were excluded from the statistical analysis in this study. Furthermore, no incidental abnormalities involving the Y chromosome were detected among the samples analyzed. In light of these considerations, this study did not conduct further statistical analysis on sex chromosome–related changes and instead concentrated on the characteristics of incidental abnormalities in the autosomes.

Overall, the specific distribution of incidental chromosomal abnormalities likely reflects the combined influence of multiple factors, including intrinsic chromosomal fragility, heterochromatin content, replication timing, banding variability, and differences in methodological sensitivity. Future studies integrating higher‐resolution molecular cytogenetic techniques, such as multicolor FISH, CMA, or long‐read sequencing, may help distinguish true low‐frequency structural variants from technical background noise, thereby further elucidating the biological nature of these incidental abnormalities and their potential implications for individual genomic stability.

In this study, a total of 23 morphologically diverse marker chromosomes were identified, demonstrating significant heterogeneity in terms of size, chromosomal origin, and banding patterns. Marker chromosomes, a prevalent form of abnormality in peripheral blood karyotype analysis, have garnered considerable attention due to their formation mechanisms and clinical implications. These chromosomes can originate from any chromosomal fragment and typically represent small structural rearrangements or ring chromosomes with variable gene content. The formation of marker chromosomes may involve mechanisms such as incomplete chromosomal rejoining postbreakage, ring chromosome formation, or partial homologous recombination [37]. Aneuploid or ring structures of chromosomal fragments can be detected during in vitro culture and banding; however, their pathological significance is often challenging to ascertain due to the absence of complete banding landmarks and clonal support. Furthermore, marker chromosomes may exhibit substantial variability across individuals and cells, with some potentially representing technical artifacts or banding background noise, particularly in nonclonal or low‐proportion cell populations, thus necessitating cautious interpretation.

Although most marker chromosomes in this study were classified as incidental findings, their presence still reflects potential somatic chromosomal instability in individuals. Previous studies have indicated that the occurrence of marker chromosomes may be associated with age, sex, and chromosomal fragile sites, yet most peripheral blood marker chromosomes are not accompanied by overt clinical phenotypes [38, 39]. In clinical practice, the identification of marker chromosomes should be classified and reported based on clonality, cell proportion, and chromosomal origin. For recurrent or clearly clonal marker chromosomes, further validation using FISH, CMA, or other molecular techniques is recommended to clarify their chromosomal origin and potential genomic impact.

Collectively, the incidental abnormalities outlined above share a common characteristic: They manifest at low frequencies and do not fulfill the criteria for clonal chromosomal abnormalities. Consequently, these observations may serve as valuable references for routine cytogenetic laboratory practice. During standard karyotype analysis, chromosomal abnormalities may occasionally be detected in a single metaphase, and in some instances, two cells exhibiting the same type of abnormality may be identified. However, such findings should be approached with caution, as they do not necessarily signify a genuine chromosomal abnormality in the individual being tested. In the absence of consistent corroborative evidence, these findings should not be prematurely interpreted or reported as definitive chromosomal abnormalities.

This study constitutes a single‐center retrospective analysis, wherein the sources of the sample and the clinical indications may introduce selection bias, thereby limiting the generalizability of the findings. The analysis relied on previously collected peripheral blood karyotype data, primarily utilizing G‐banding, without validation through molecular cytogenetic techniques such as FISH or copy number variation sequencing (CNV‐seq). The retrospective nature of the study precludes the possibility of conducting additional testing; furthermore, the incidental abnormalities identified lack available material for further analysis, rendering multimethod validation impractical. Additionally, G‐banding karyotype analysis possesses inherent limitations in sensitivity, potentially overlooking small structural variants or low‐level mosaicism, which may impact the accuracy of abnormality classification. This underscores the need for confirmation in future prospective studies.

In conclusion, this study conducted a systematic analysis of the distribution of incidental chromosomal abnormalities in peripheral blood, categorized by sex, age, chromosome group, and marker chromosome specificity. The results demonstrate population‐level heterogeneity and suggest potential patterns in somatic chromosomal structure. These findings enrich the current understanding of the spectrum of chromosomal abnormalities and provide a useful reference for routine cytogenetic laboratory practice. Future research incorporating high‐resolution molecular techniques and longitudinal follow‐up is recommended to further elucidate the molecular basis of incidental chromosomal abnormalities and their association with health and disease risk.

## Author Contributions

Yaqin Zhang designed the study, performed data analysis, and primarily wrote the manuscript. Zongkai Bai and Rui Li conducted the experiments, data entry, and data organization. Han Yang and Jinfeng Wang assisted in data curation and management. Yiying Chen provided resources and supervised the study.

## Funding

This study was supported by the Pu’er City Joint Special Program for Medical Research (2025) (YXLH202525, YXLH202526, and YXLH202527).

## Disclosure

All authors reviewed and approved the final manuscript.

## Ethics Statement

This retrospective study was approved by the Ethics Committee of Pu’er People’s Hospital. As the study utilized previously collected clinical testing data and all data were anonymized, the requirement for informed consent was waived by the Ethics Committee.

## Conflicts of Interest

The authors declare no conflicts of interest.

## Supporting Information

Additional supporting information can be found online in the Supporting Information section.

## Supporting information


**Supporting Information 1** TABLE S1: Clinical characteristics of the enrolled subjects and a comprehensive summary of incidentally detected chromosomal abnormalities for each case.

**Supporting Information 2** FIGURE S1: Representative karyotype images of incidentally detected abnormalities involving chromosome 1.

**Supporting Information 3** FIGURE S2: Representative karyotype images of incidentally detected abnormalities involving chromosome 2.

**Supporting Information 4** FIGURE S3: Representative karyotype images of incidentally detected abnormalities involving chromosome 3.

**Supporting Information 5** FIGURE S4: Representative karyotype images of incidentally detected abnormalities involving chromosome 4.

**Supporting Information 6** FIGURE S5: Representative karyotype images of incidentally detected abnormalities involving chromosome 5.

**Supporting Information 7** FIGURE S6: Representative karyotype images of incidentally detected abnormalities involving chromosome 6.

**Supporting Information 8** FIGURE S7: Representative karyotype images of incidentally detected abnormalities involving chromosome 7.

**Supporting Information 9** FIGURE S8: Representative karyotype images of incidentally detected abnormalities involving chromosome 8.

**Supporting Information 10** FIGURE S9: Representative karyotype images of incidentally detected abnormalities involving chromosome 9.

**Supporting Information 11** FIGURE S10: Representative karyotype images of incidentally detected abnormalities involving chromosomes 10, 12, and 13.

**Supporting Information 12** FIGURE S11: Representative karyotype images of incidentally detected abnormalities involving chromosome 11.

**Supporting Information 13** FIGURE S12: Representative karyotype images of incidentally detected abnormalities involving chromosome 14.

**Supporting Information 14** FIGURE S13: Representative karyotype images of incidentally detected abnormalities involving chromosomes 15, 16, 18, 20, and 22.

## Data Availability

All data generated and/or analyzed during this study are included in this published article and its supporting information files. The raw data supporting the findings of this study are provided in the supporting file entitled “Table S1.”
