# Supplementary figures and images for: Frequency and Distribution of Incidental Chromosomal Abnormalities Detected by Peripheral Blood Karyotyping: A Retrospective Study
Source: Genet Res (Camb). 2026 May 17;2026:4906805. doi: 10.1155/genr/4906805 (PMC13181144; doi:10.1155/genr/4906805)

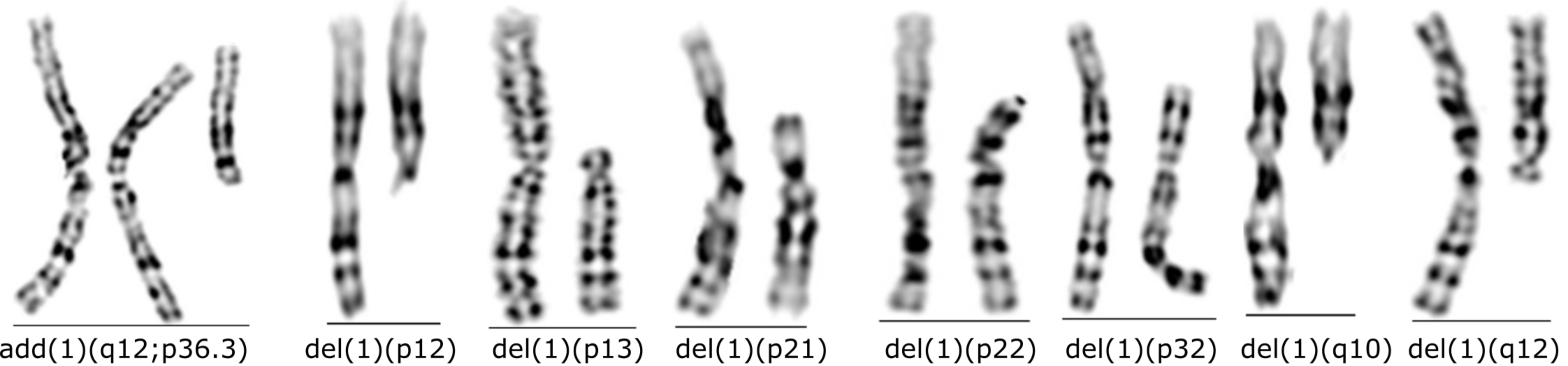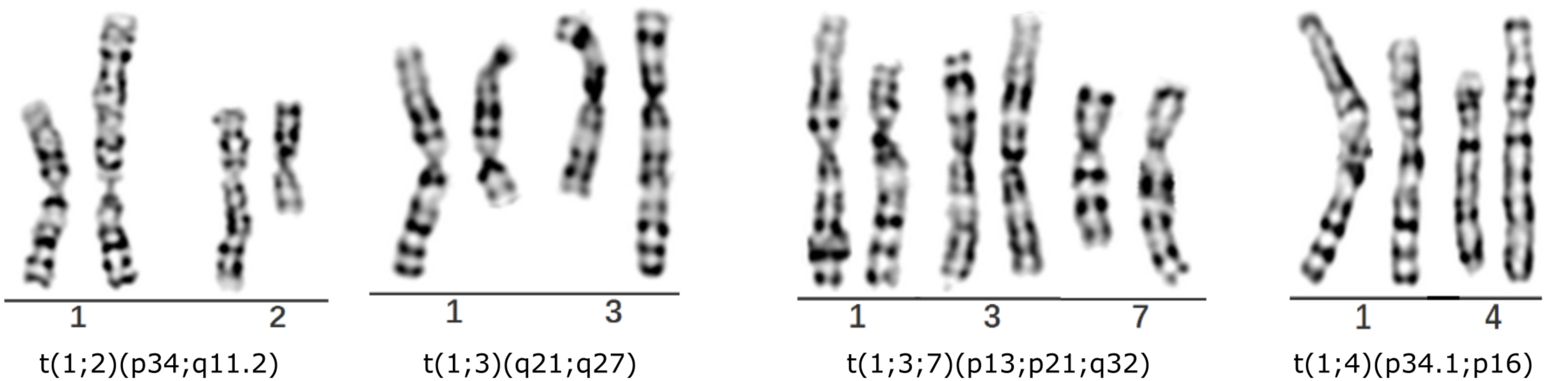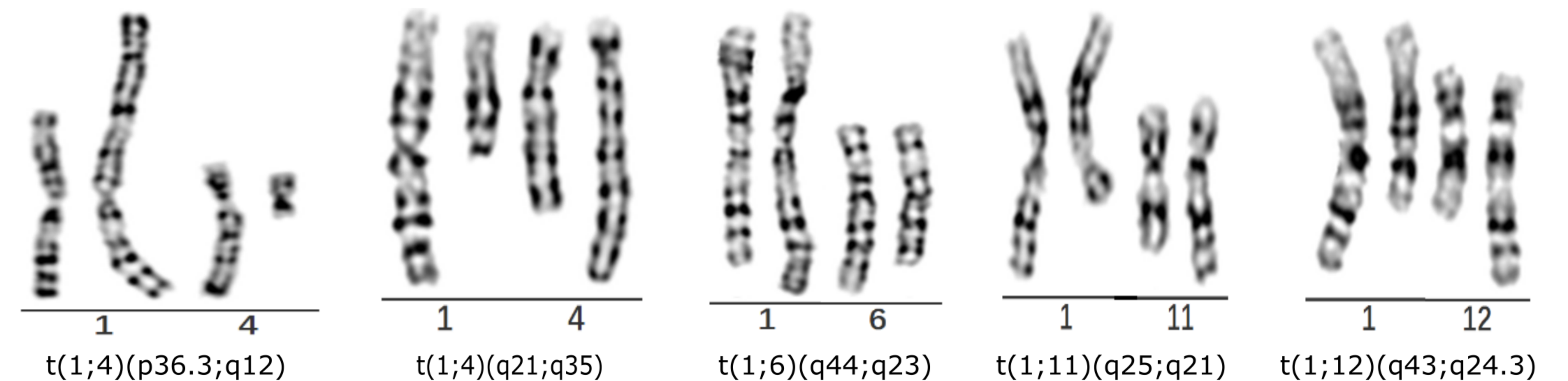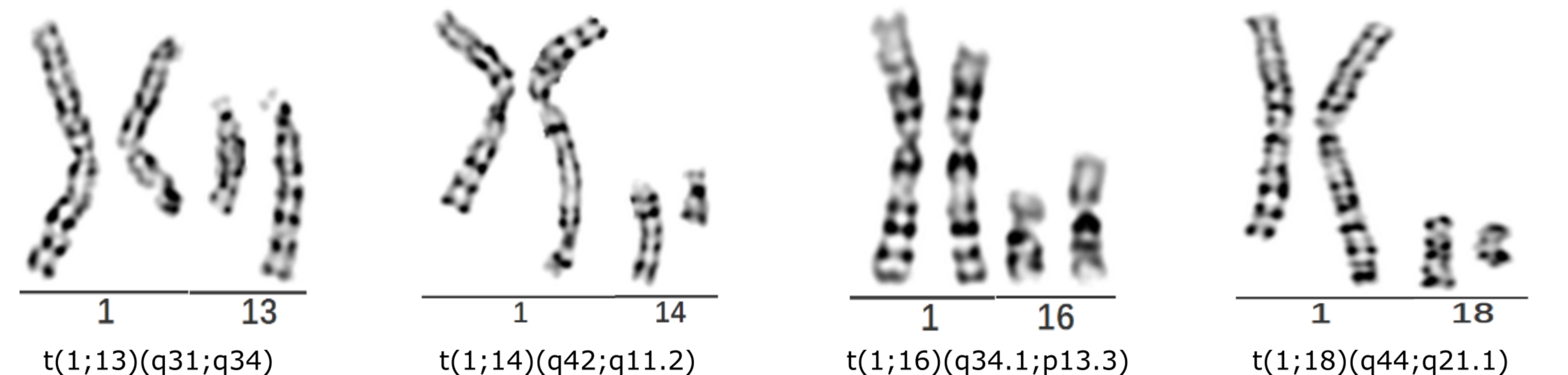

Supplement: Supplementary file 2 — Supporting Information 2 FIGURE S1: Representative karyotype images of incidentally detected abnormalities involving chromosome 1. [file GENR-2026-4906805-s001.pdf]

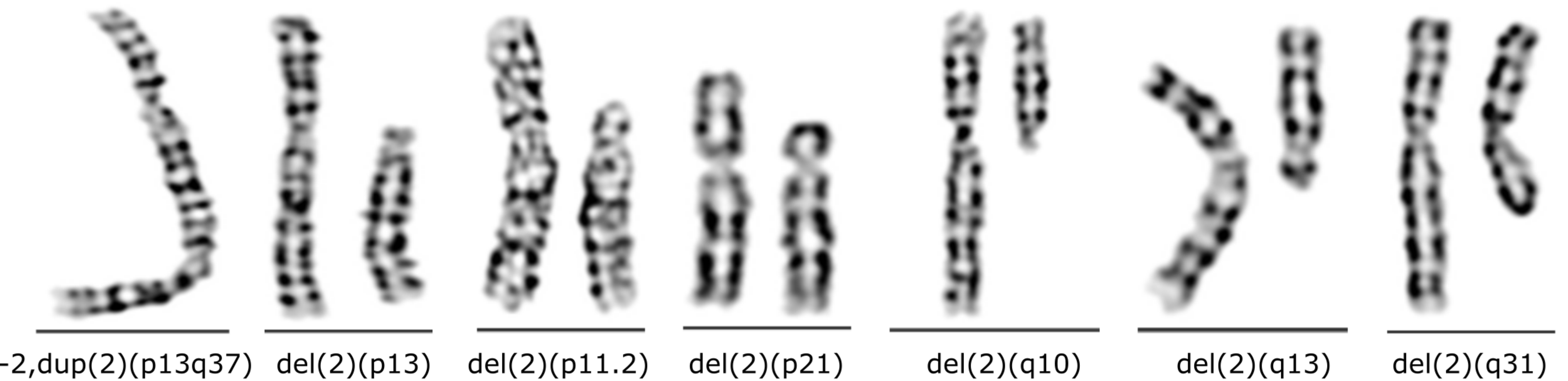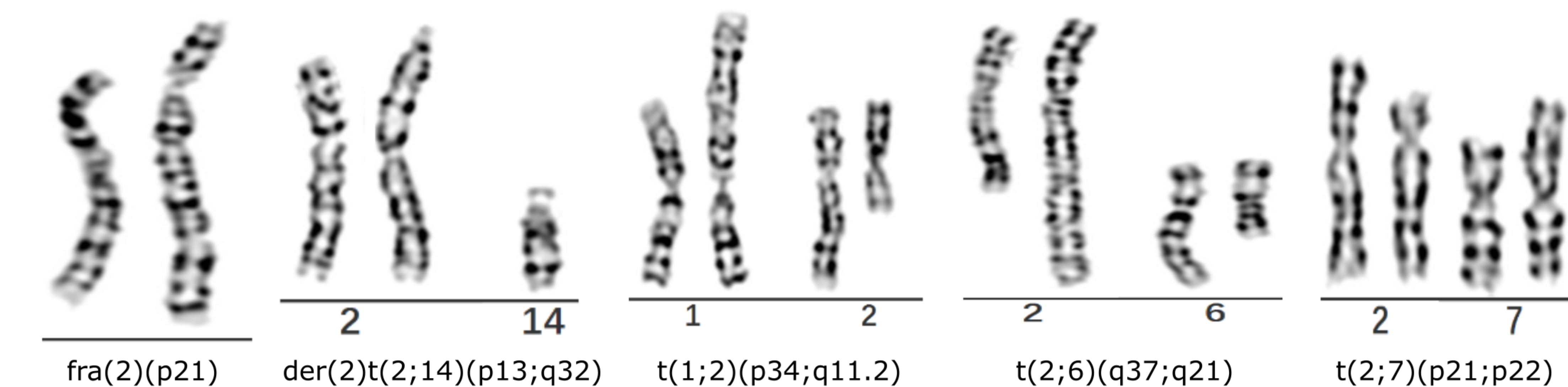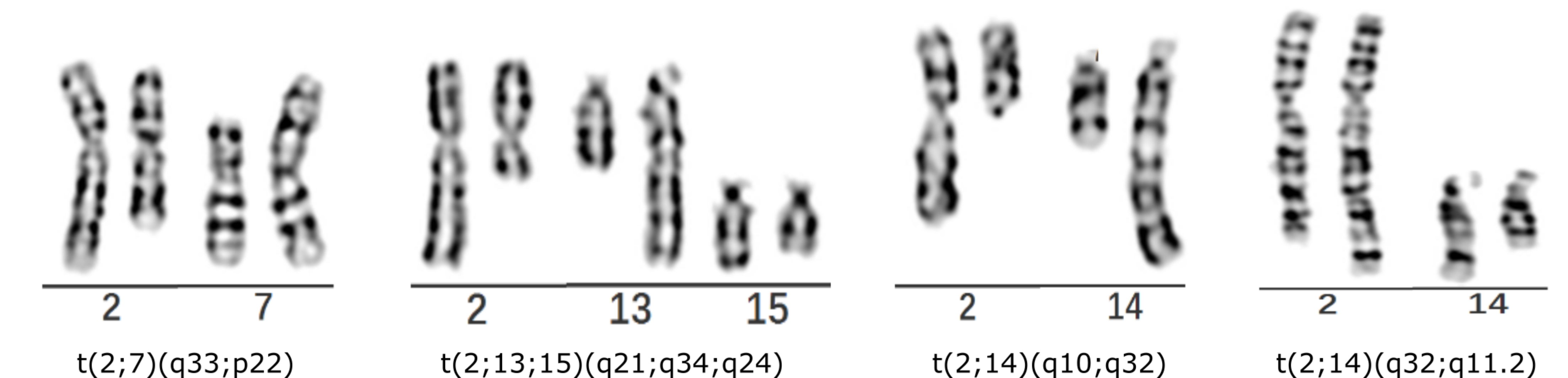

Supplement: Supplementary file 3 — Supporting Information 3 FIGURE S2: Representative karyotype images of incidentally detected abnormalities involving chromosome 2. [file GENR-2026-4906805-s002.pdf]

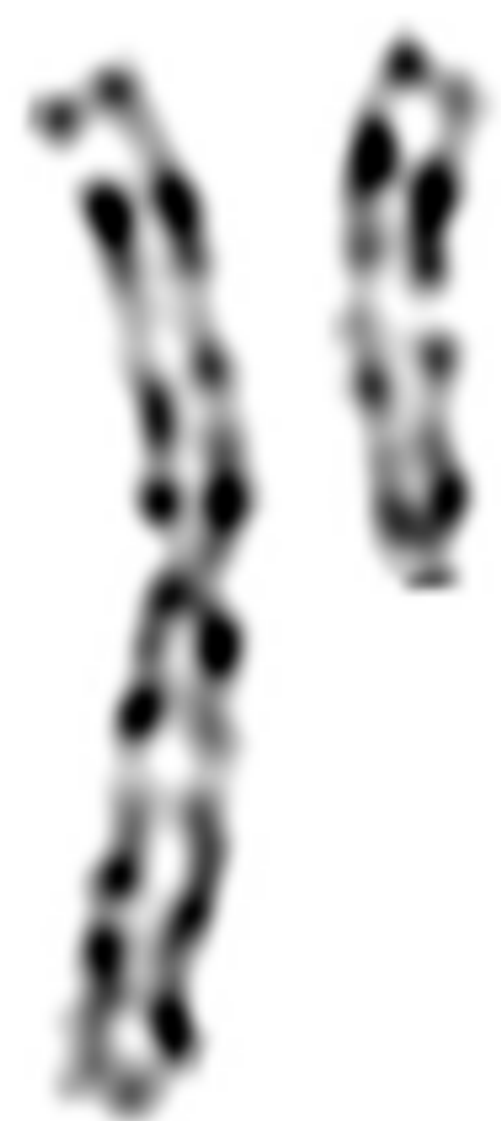

del(3)(p10)

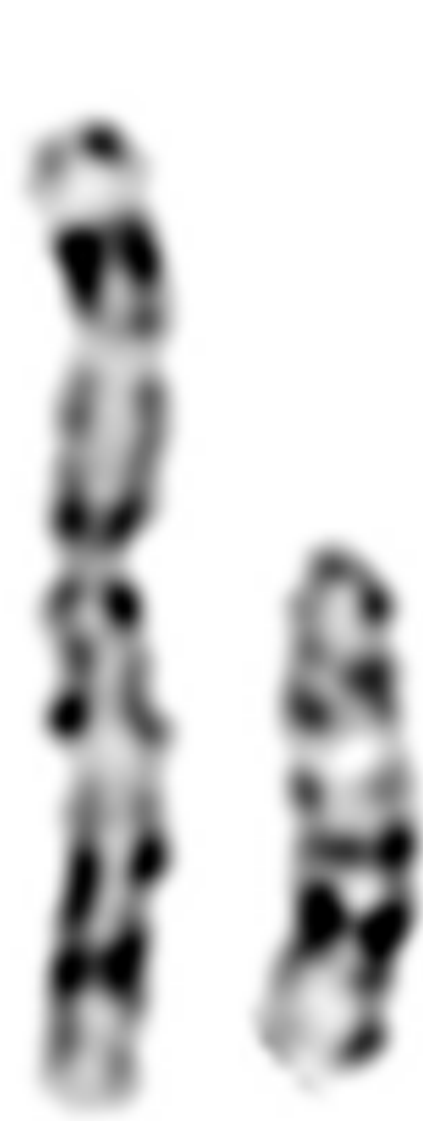

del(3)(q10)

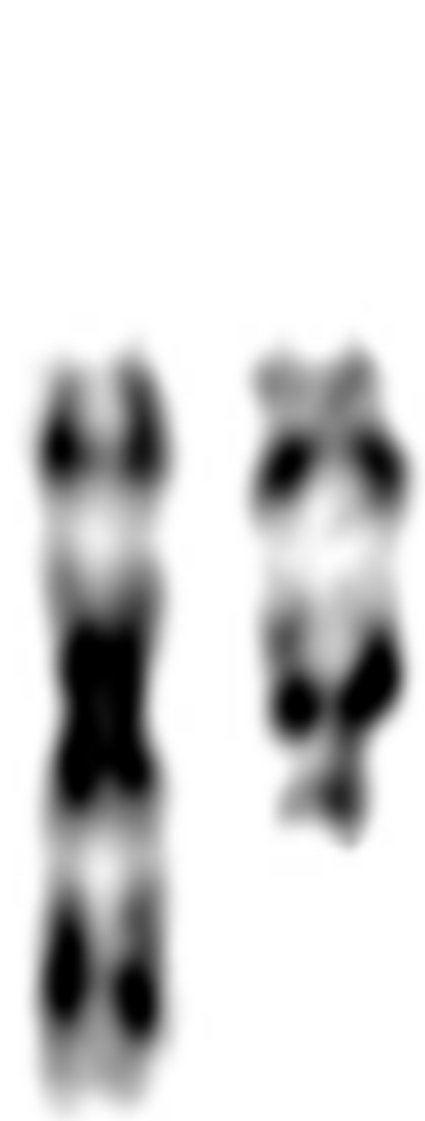

del(3)(q12)

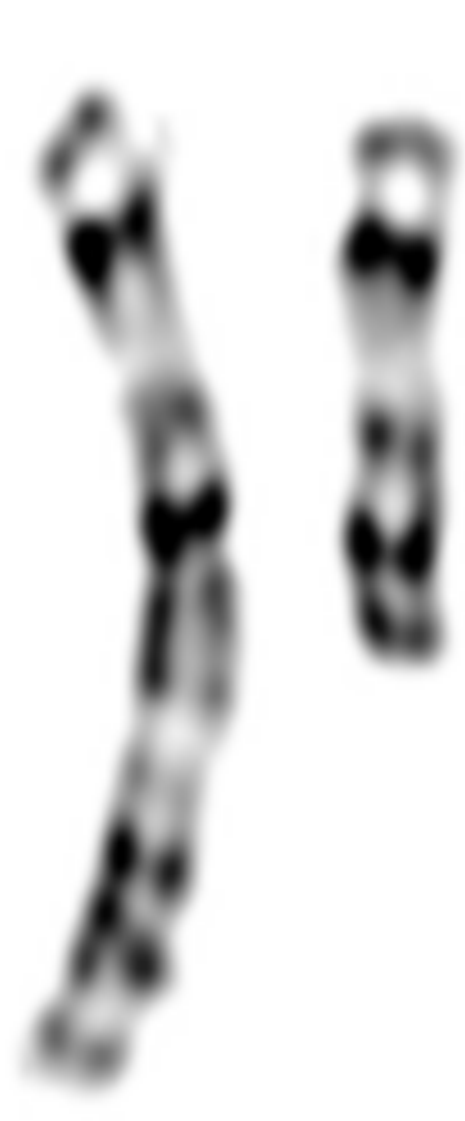

del(3)(q13.1)

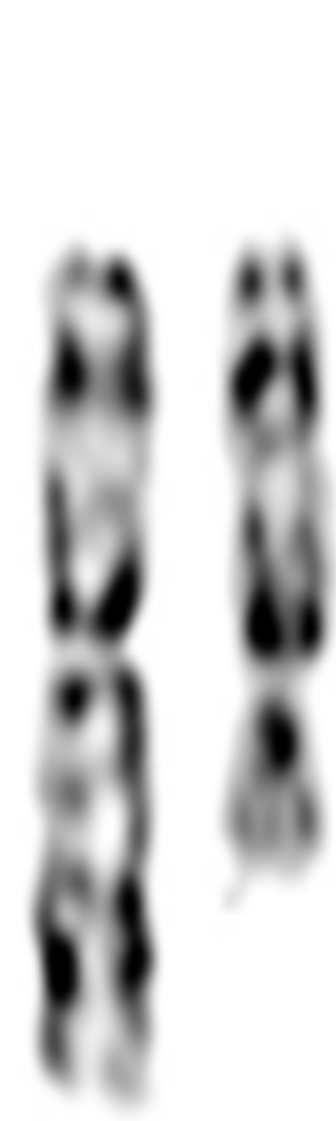

del(3)(q13.2)

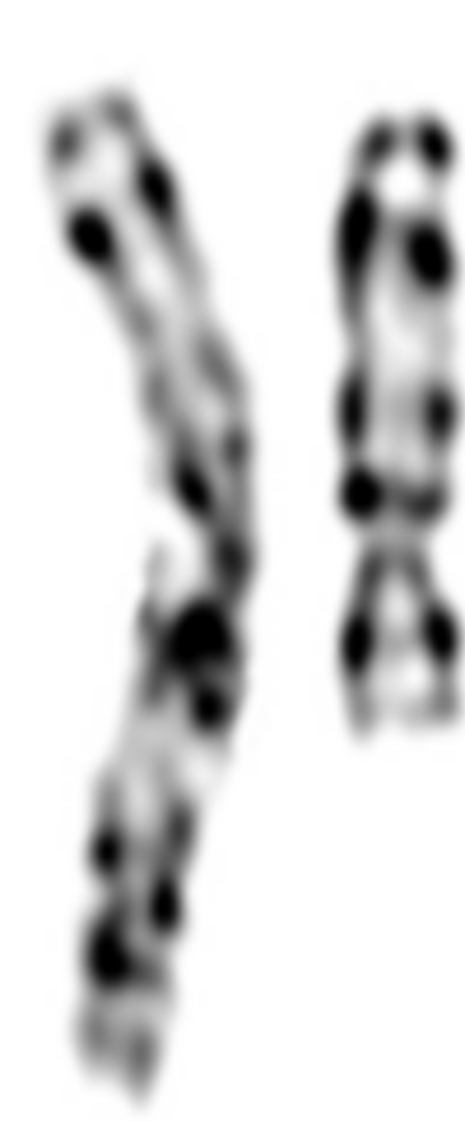

del(3)(q21)

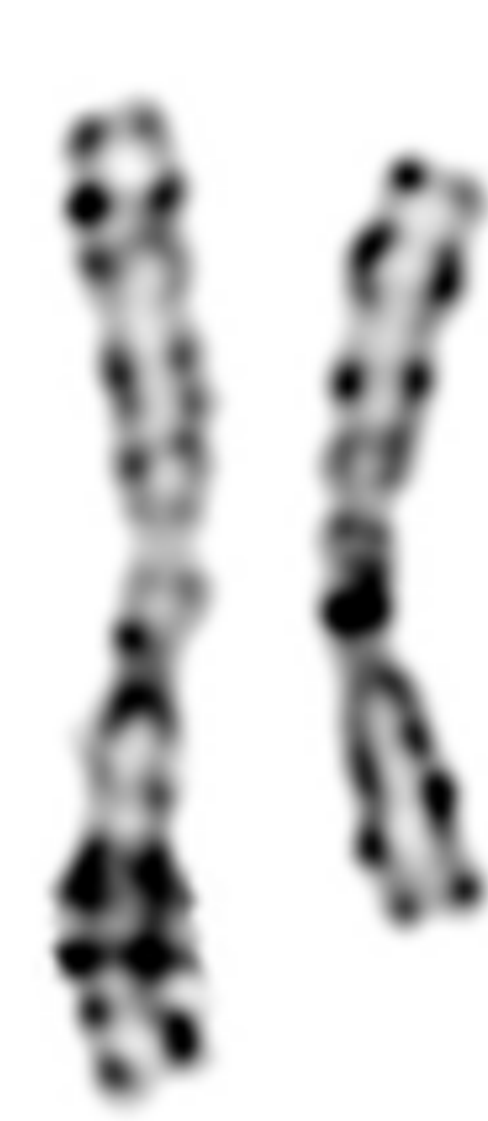

del(3)(q23)

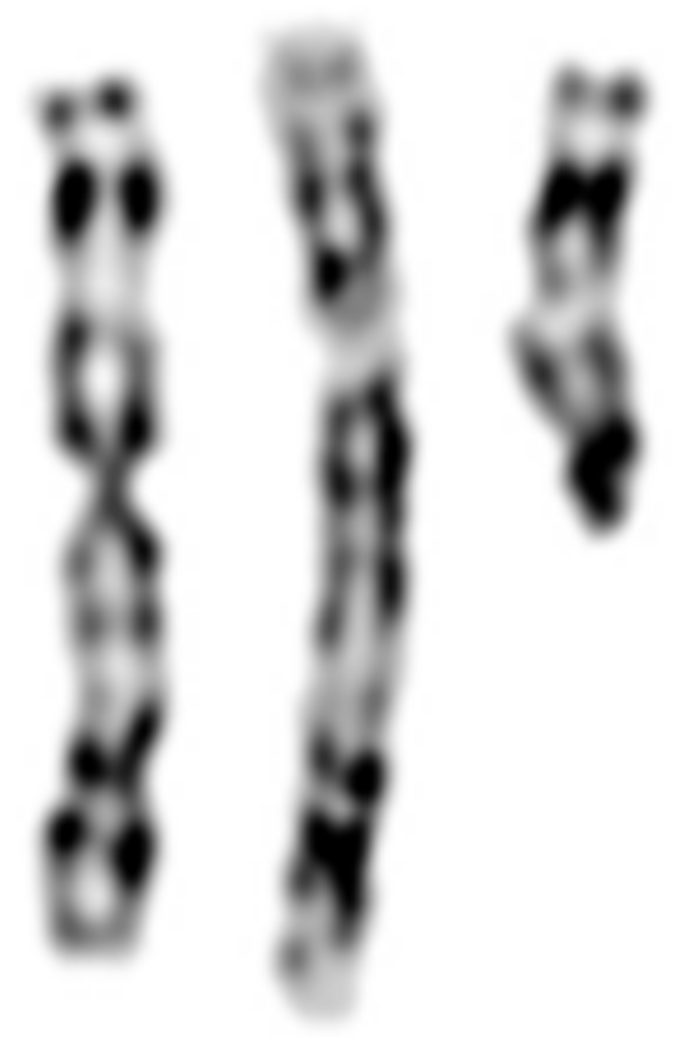

+der(3)del(3)(q11.2),i(3)(q10)

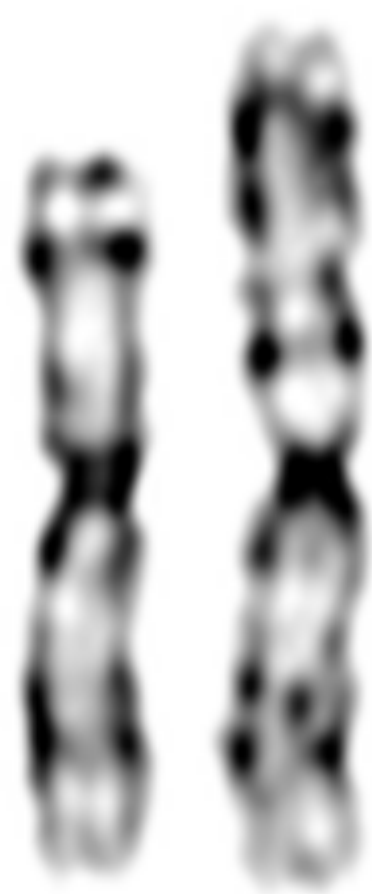

3

5

14

18

t(3;5;14;18)(p13;q13;q32;q23)

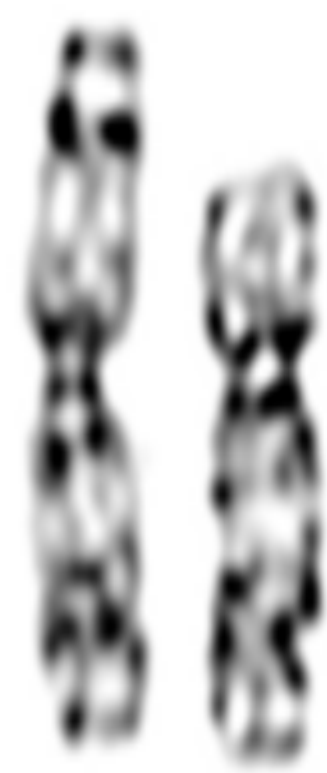

3

7

t(3;7)(p21;q36)

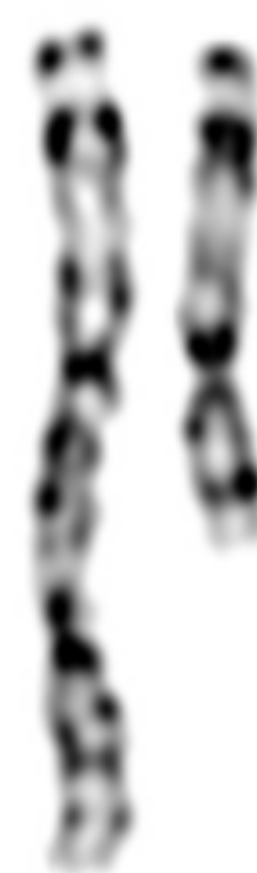

3

7

t(3;7)(q21;p21)

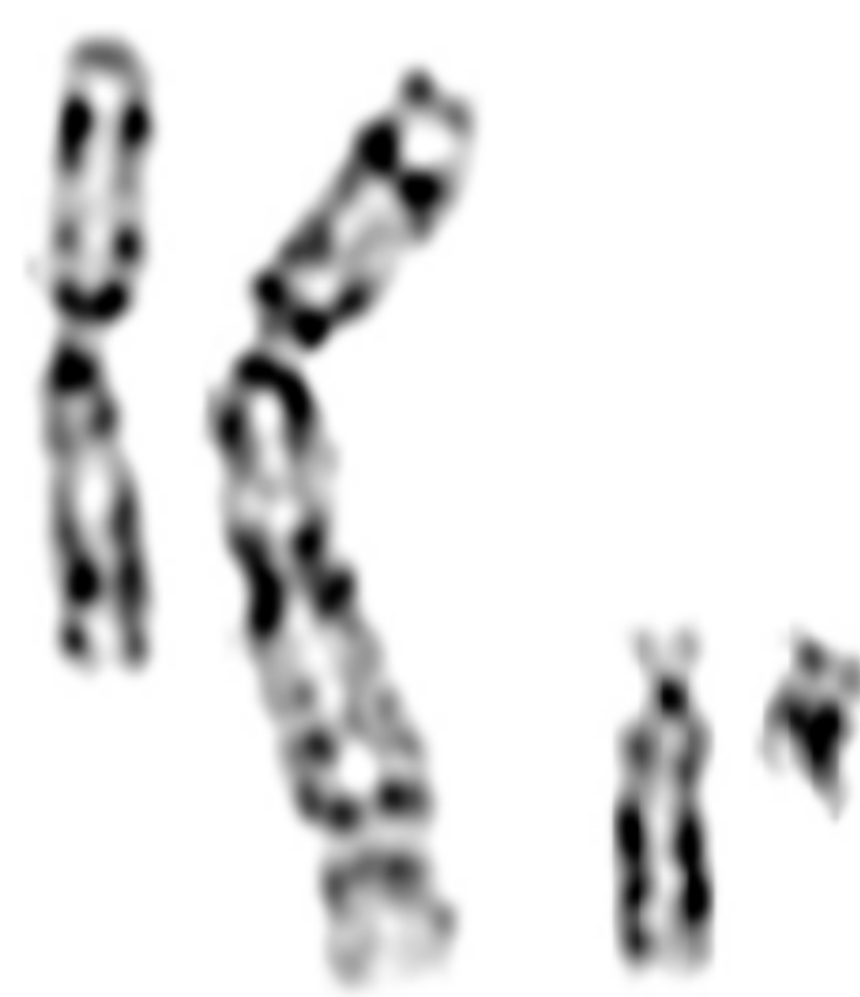

3

13

t(3;13)(q29;q12.1)

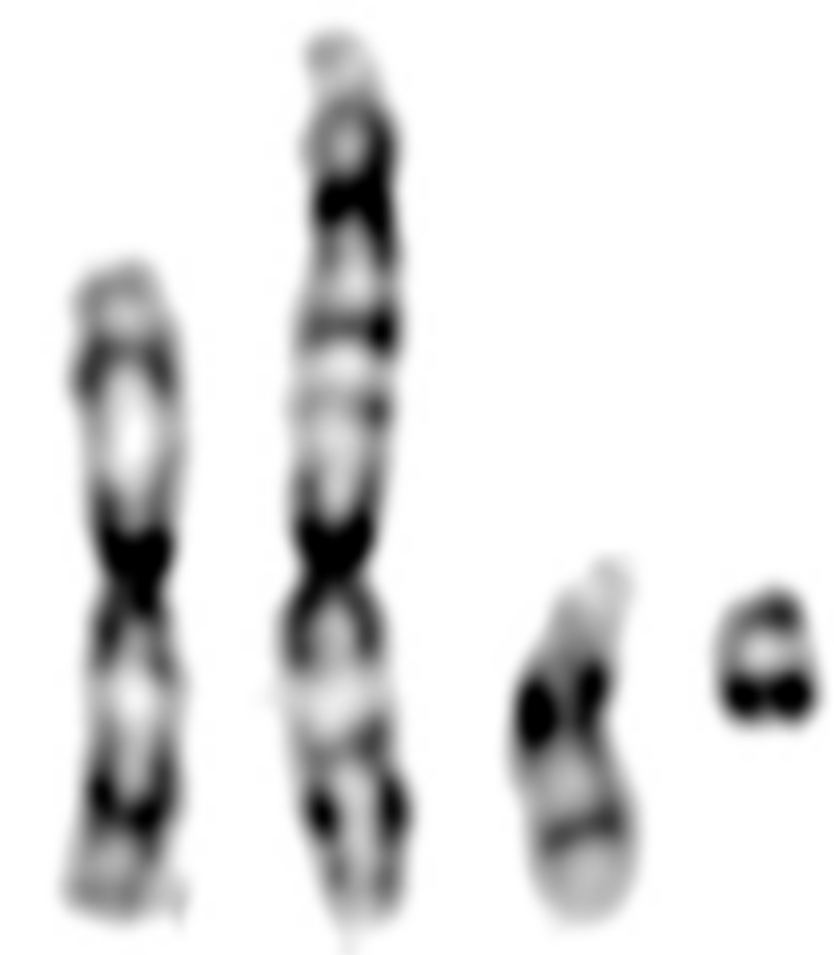

3

14

t(3;14)(p23;q32)

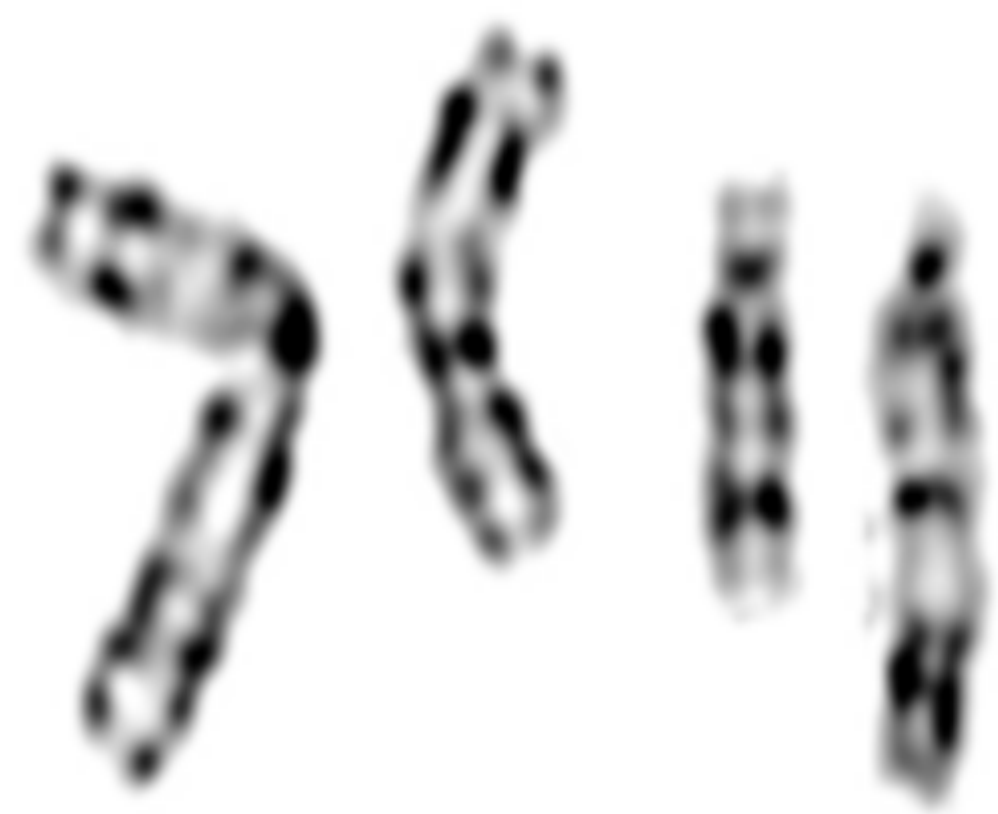

3

14

t(3;14)(q21;q32)

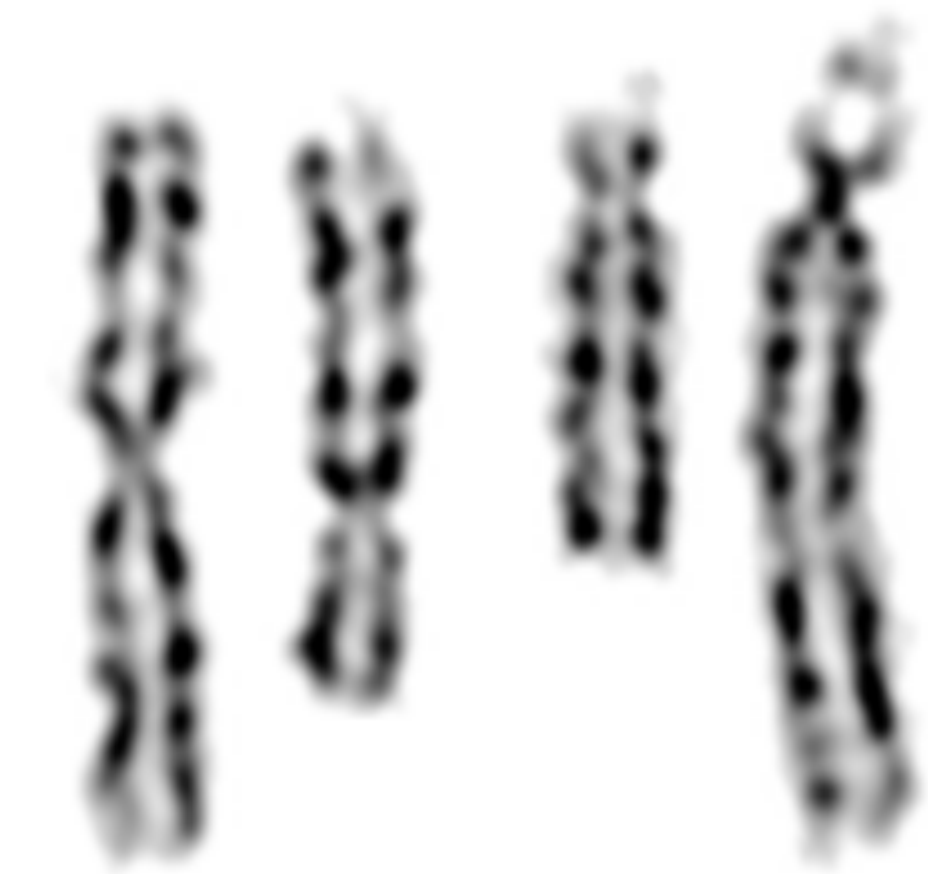

3

15

t(3;15)(q21;q26.3)

Supplement: Supplementary file 4 — Supporting Information 4 FIGURE S3: Representative karyotype images of incidentally detected abnormalities involving chromosome 3. [file GENR-2026-4906805-s003.pdf]

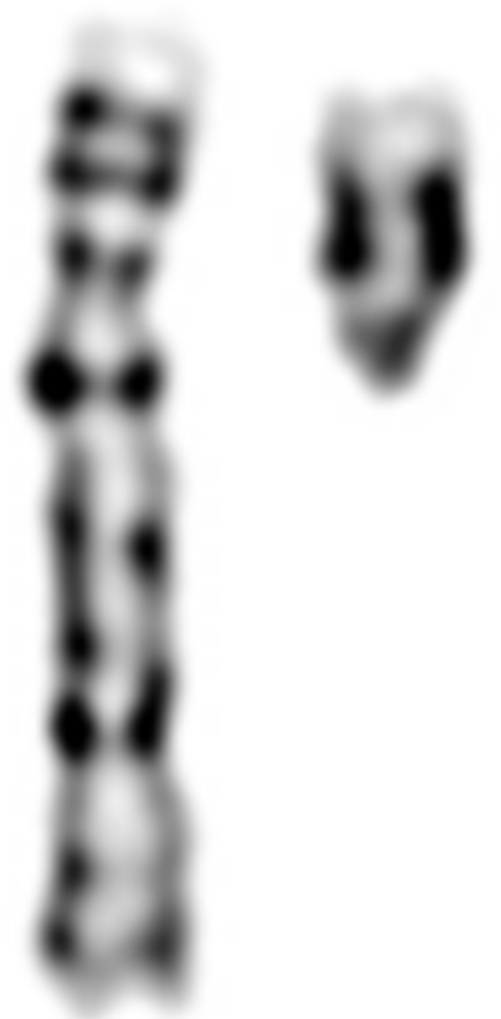

del(4)(q10)

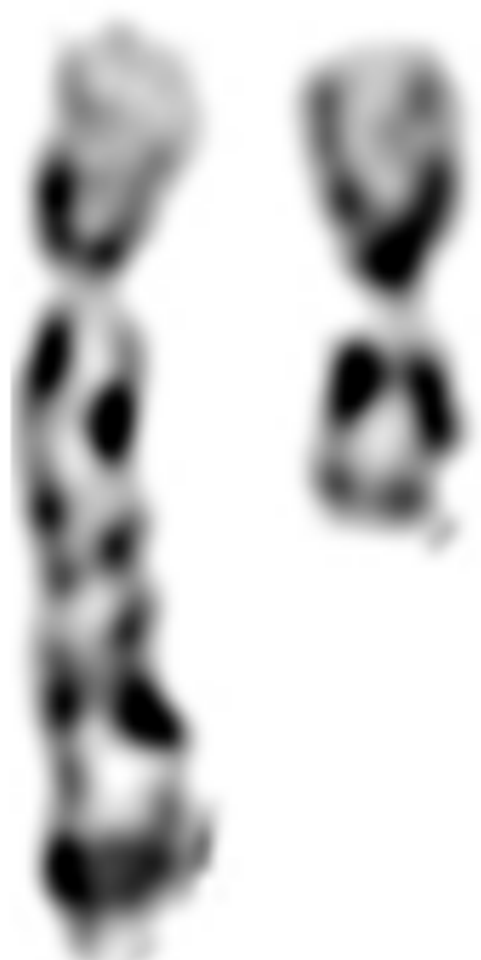

del(4)(q22)

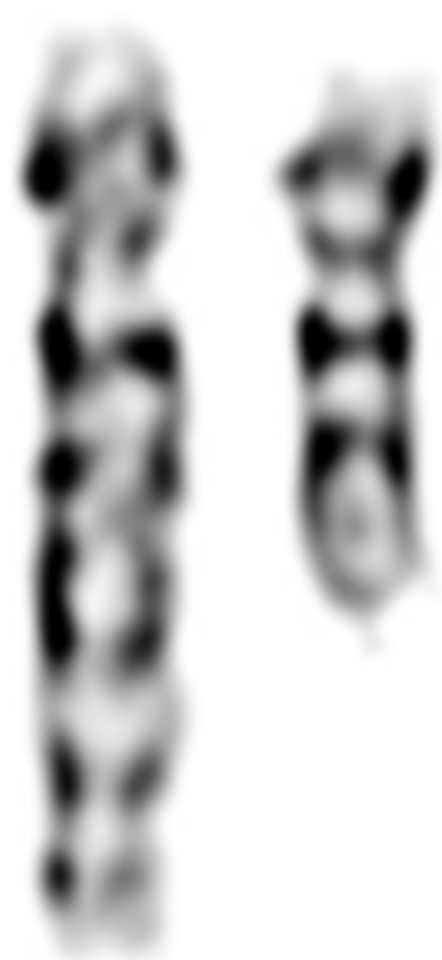

del(4)(q25)

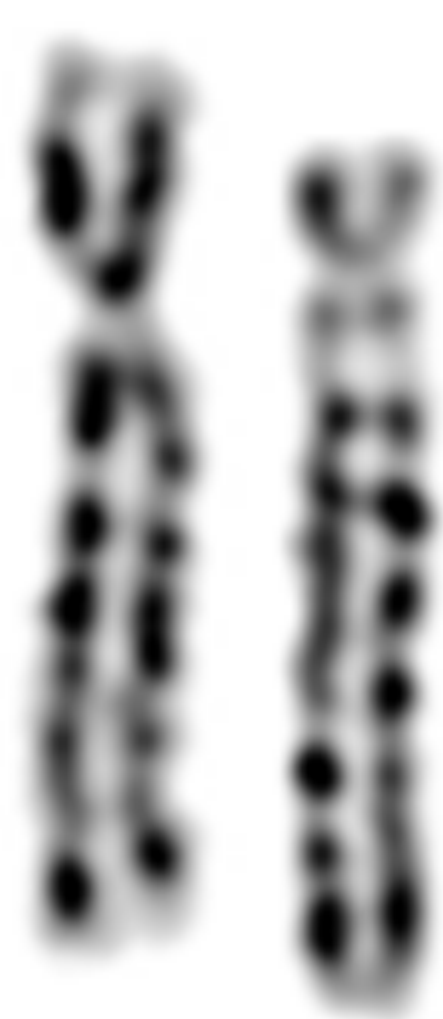

inv(4)(p16q21)

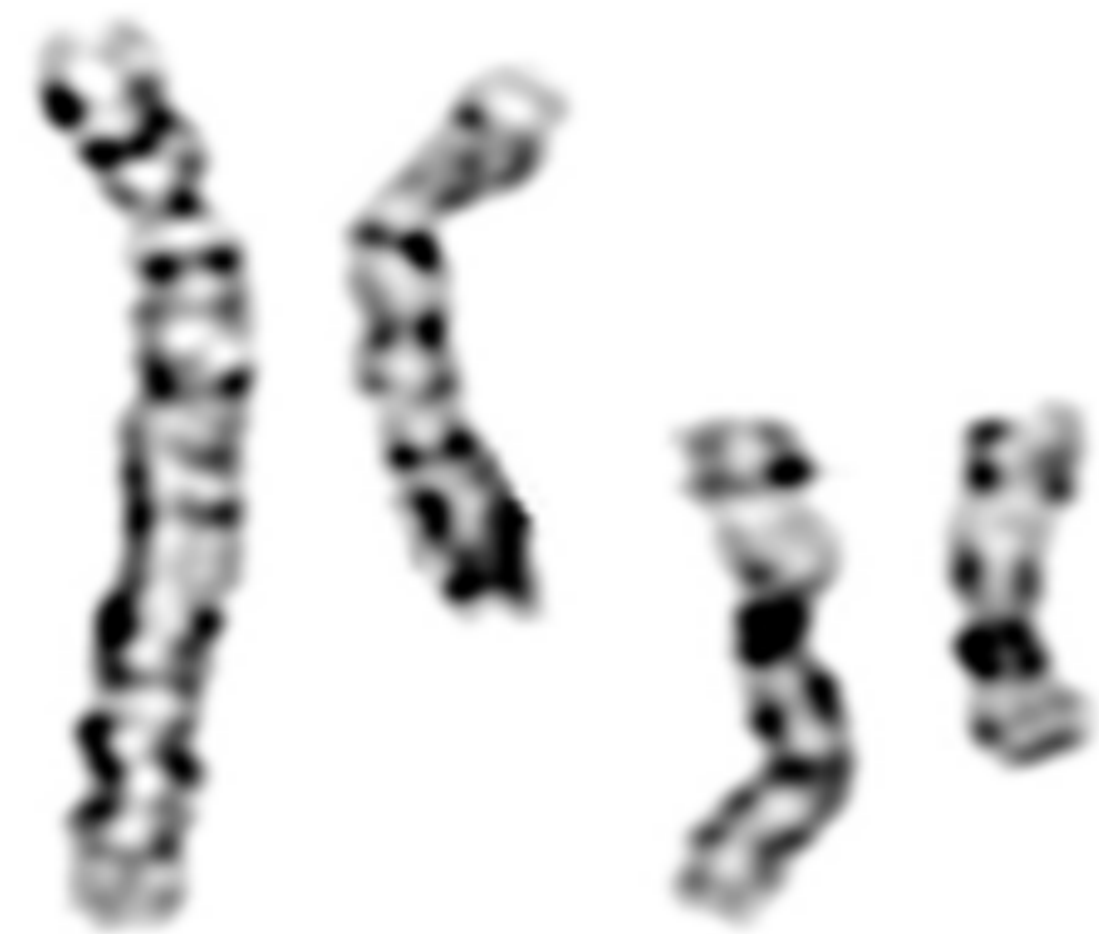

4 6  
t(4;6)(q31.3;q13)

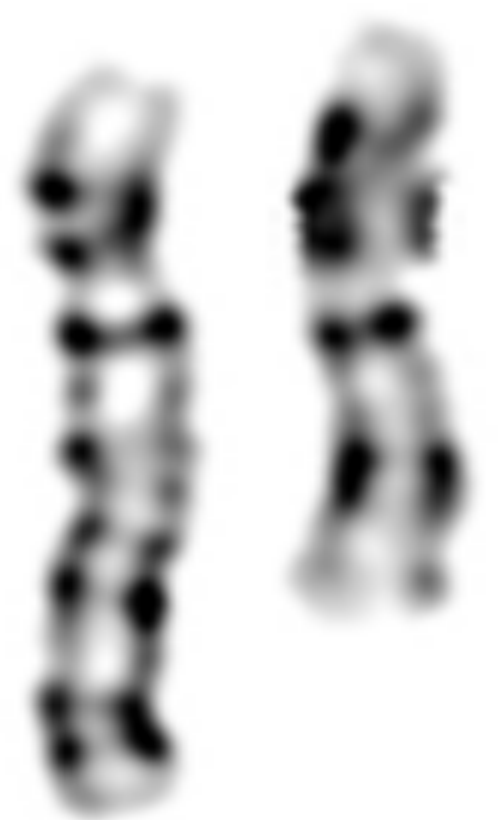

4 11  
t(4;11)(q21;p13)

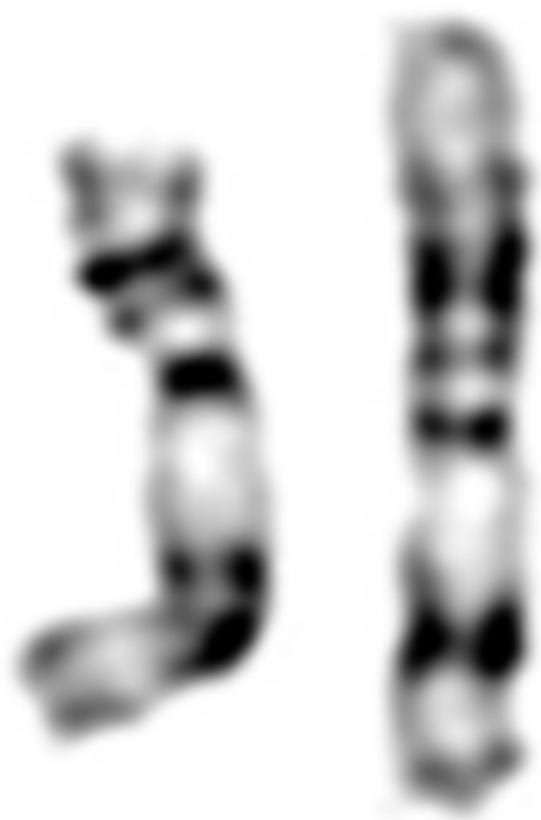

4 12  
t(4;12)(q35;q13)

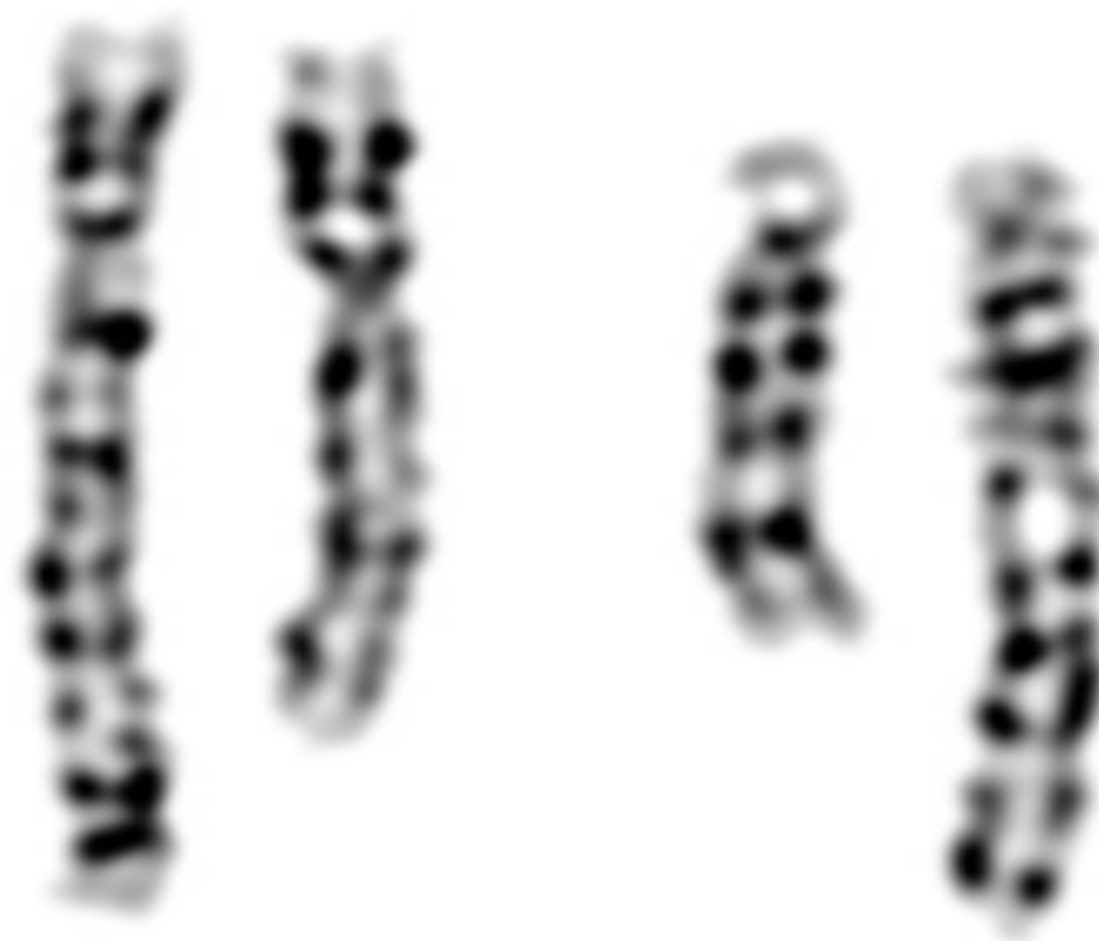

4 14  
t(4;14)(q21;q22)

Supplement: Supplementary file 5 — Supporting Information 5 FIGURE S4: Representative karyotype images of incidentally detected abnormalities involving chromosome 4. [file GENR-2026-4906805-s004.pdf]

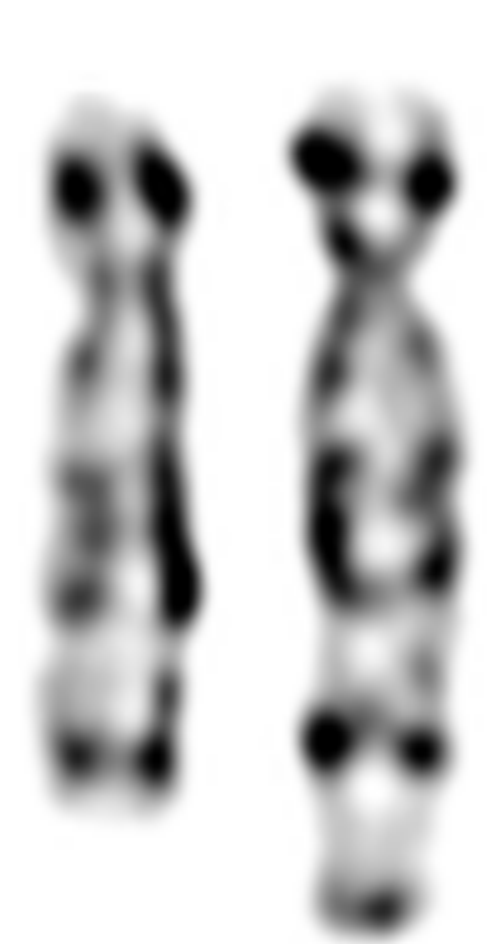

add(5)(q35)

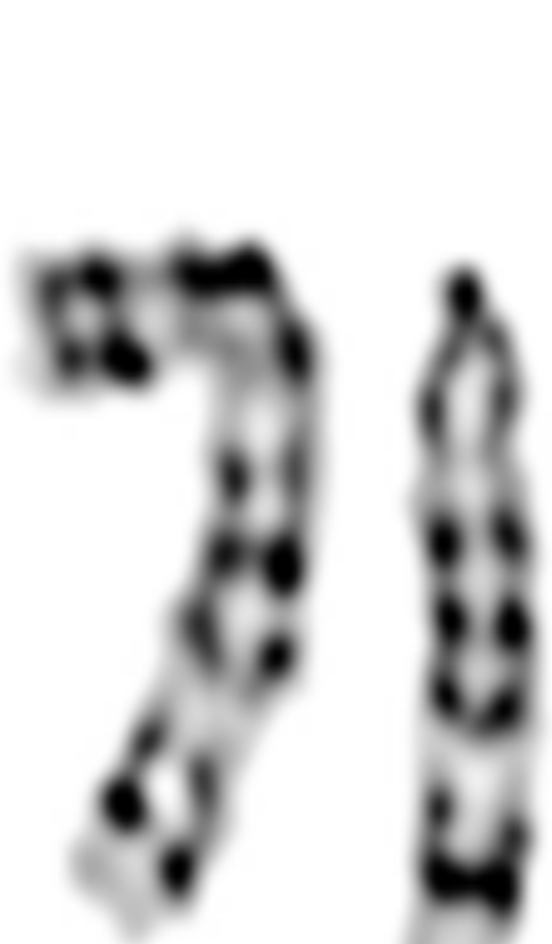

del(5)(p12)

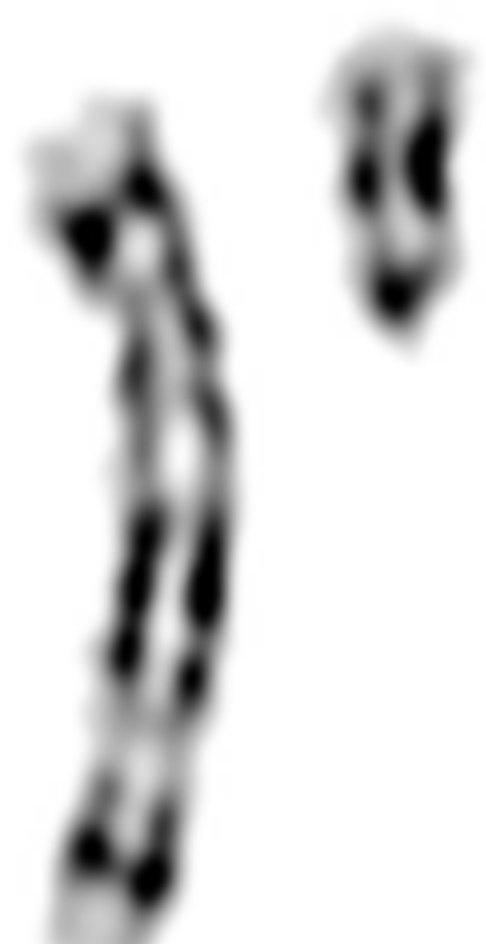

del(5)(q10)

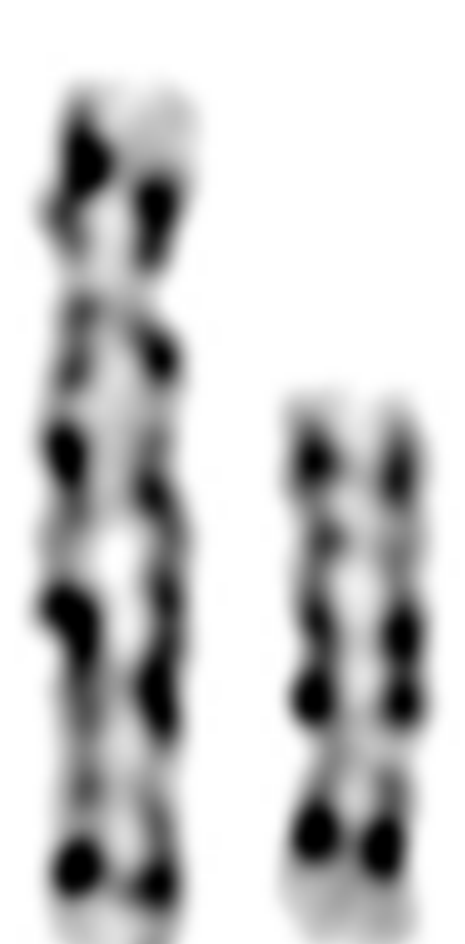

del(5)(q13)

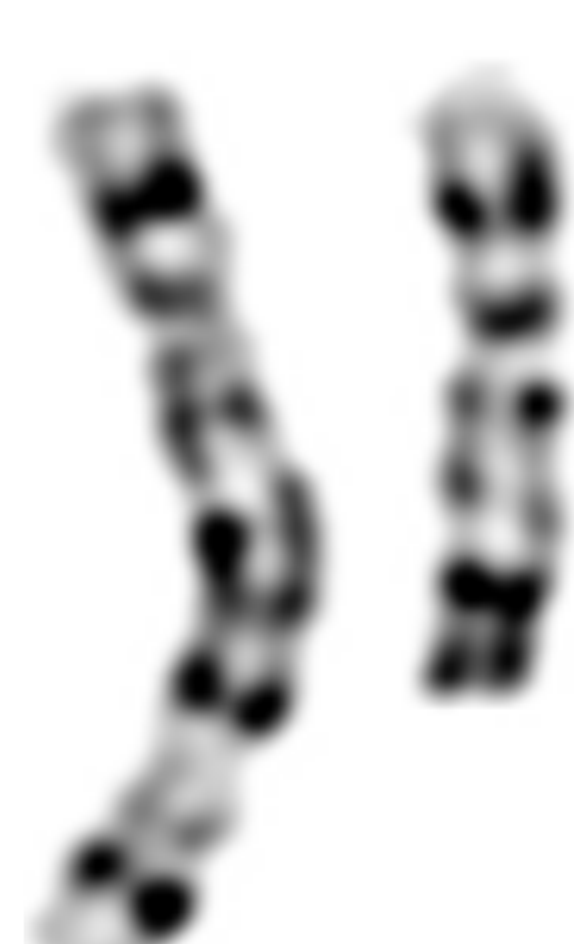

del(5)(q22)

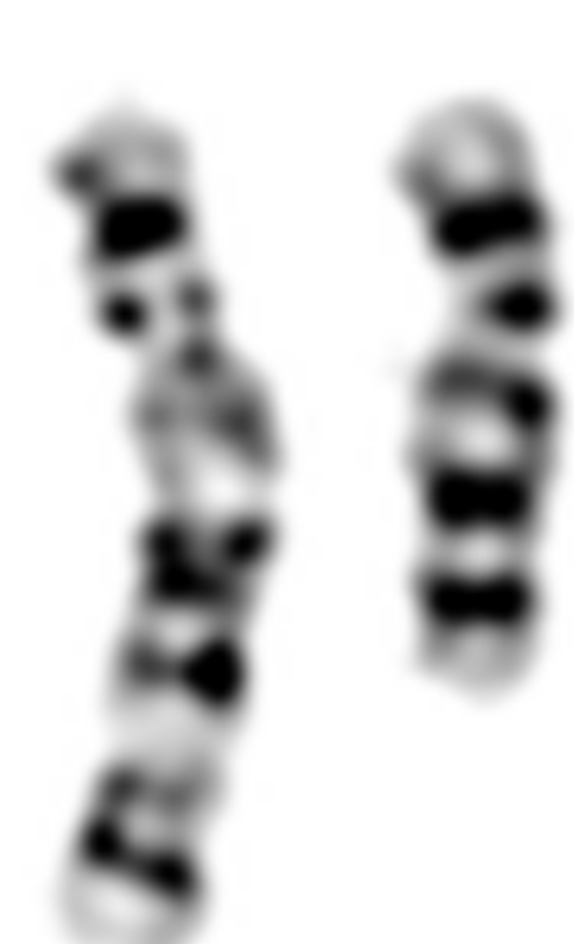

del(5)(q31)

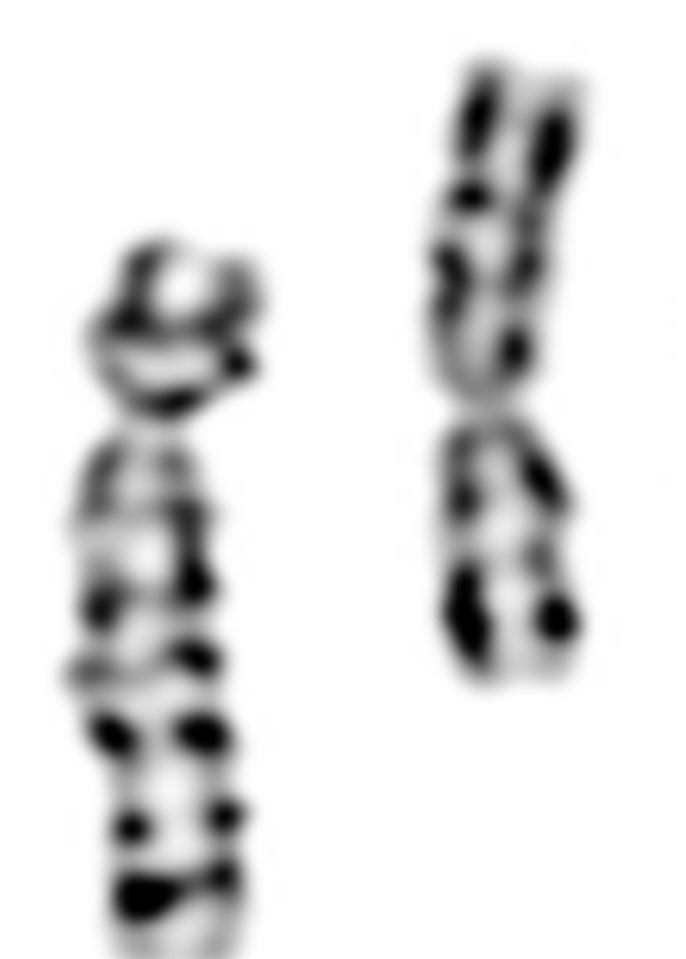

5  
inv(5)(p13q22)

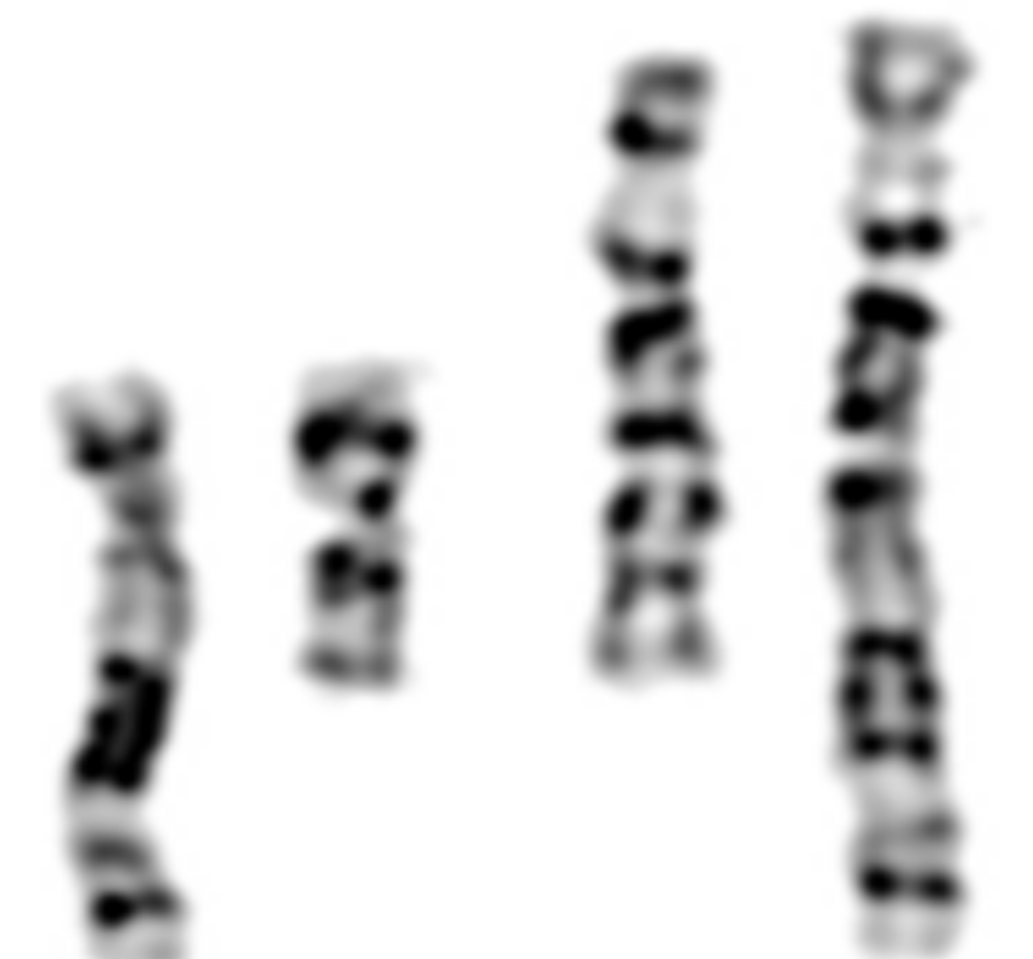

5 6  
t(5;6)(q13;q25)

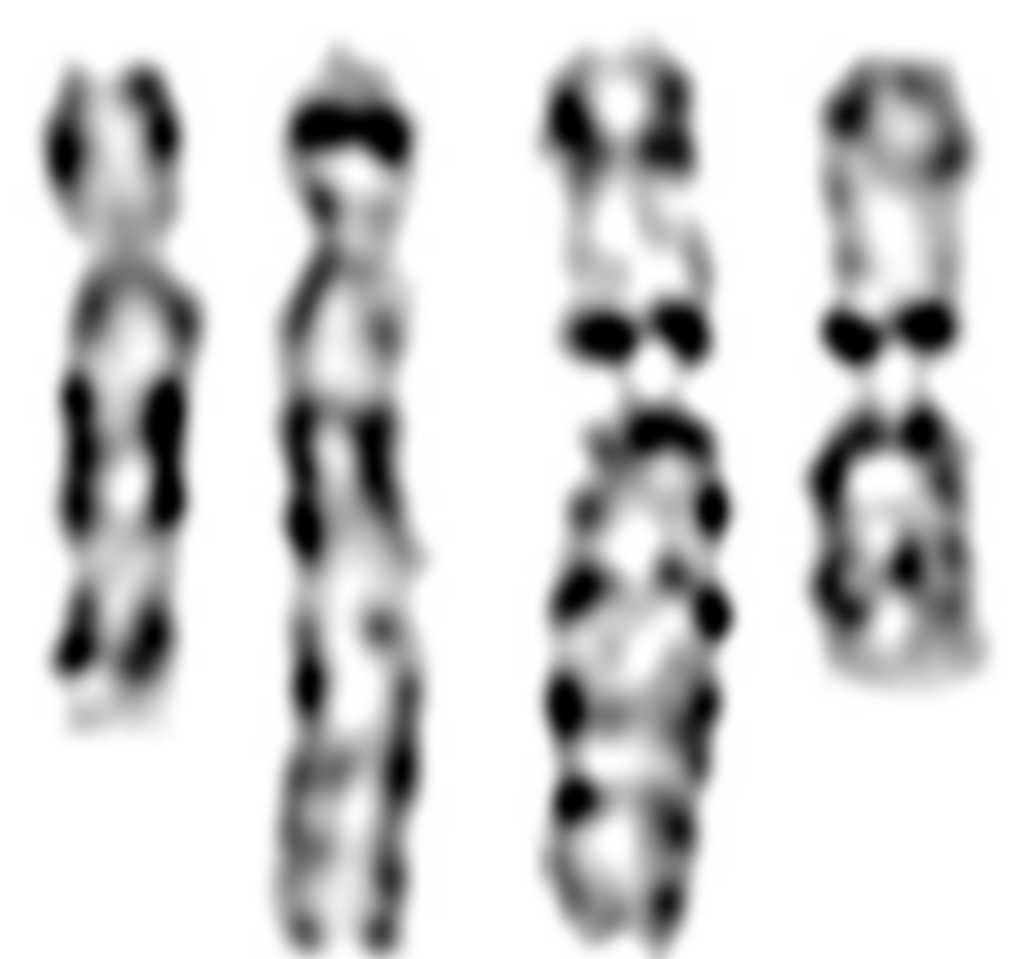

5 6  
t(5;6)(q35;q21)

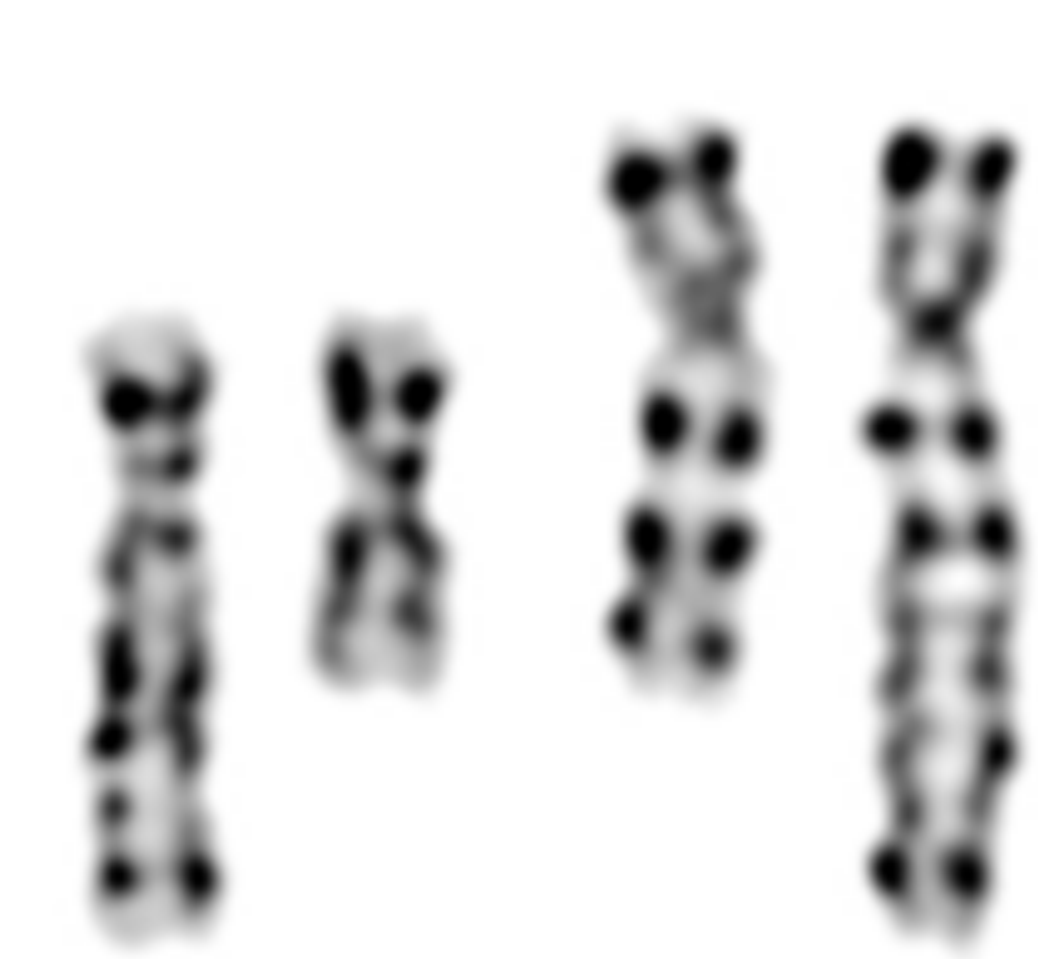

5 7  
t(5;7)(q13;q32)

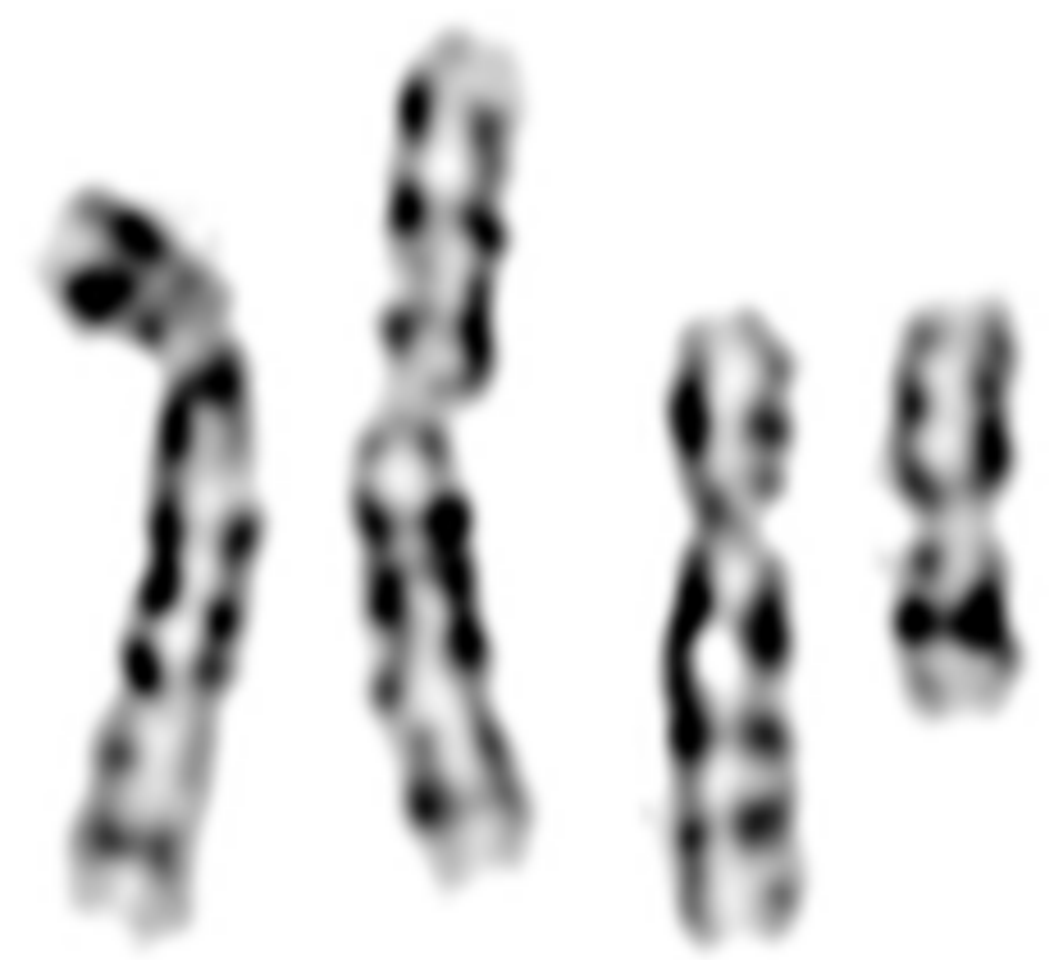

5 10  
t(5;10)(q10;q10)

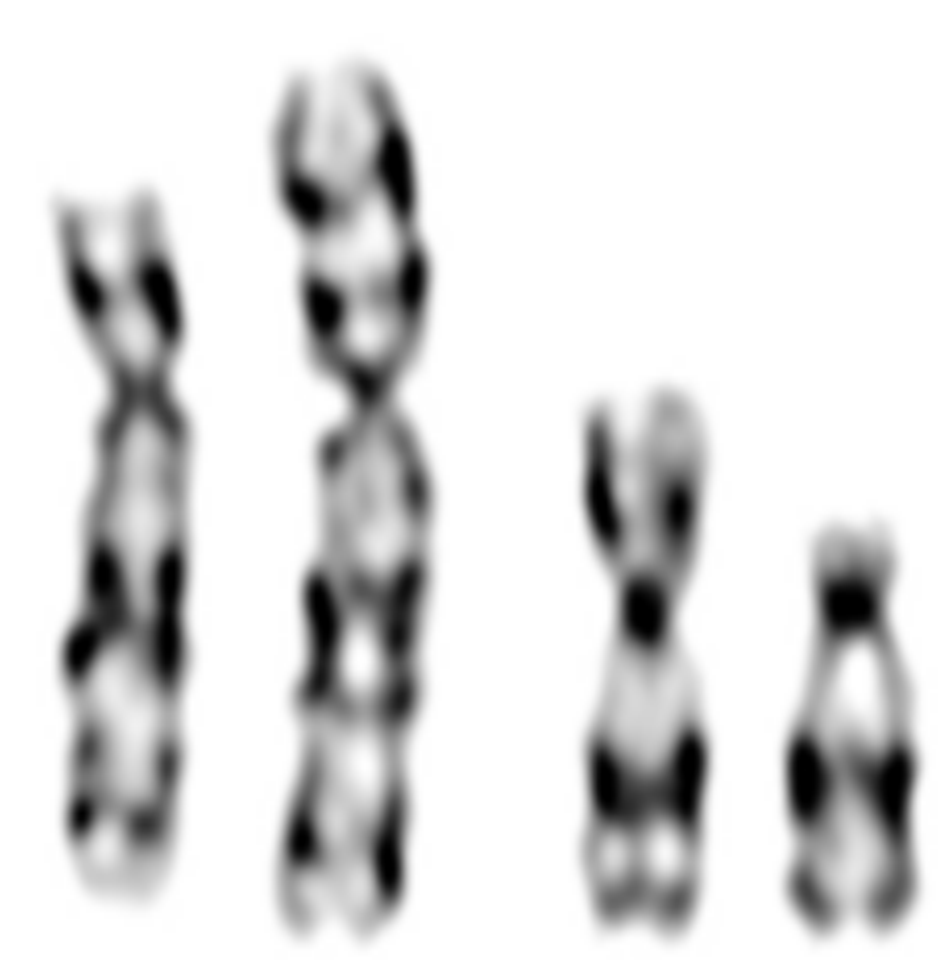

5 11  
t(5;11)(p15.3;p11.2)

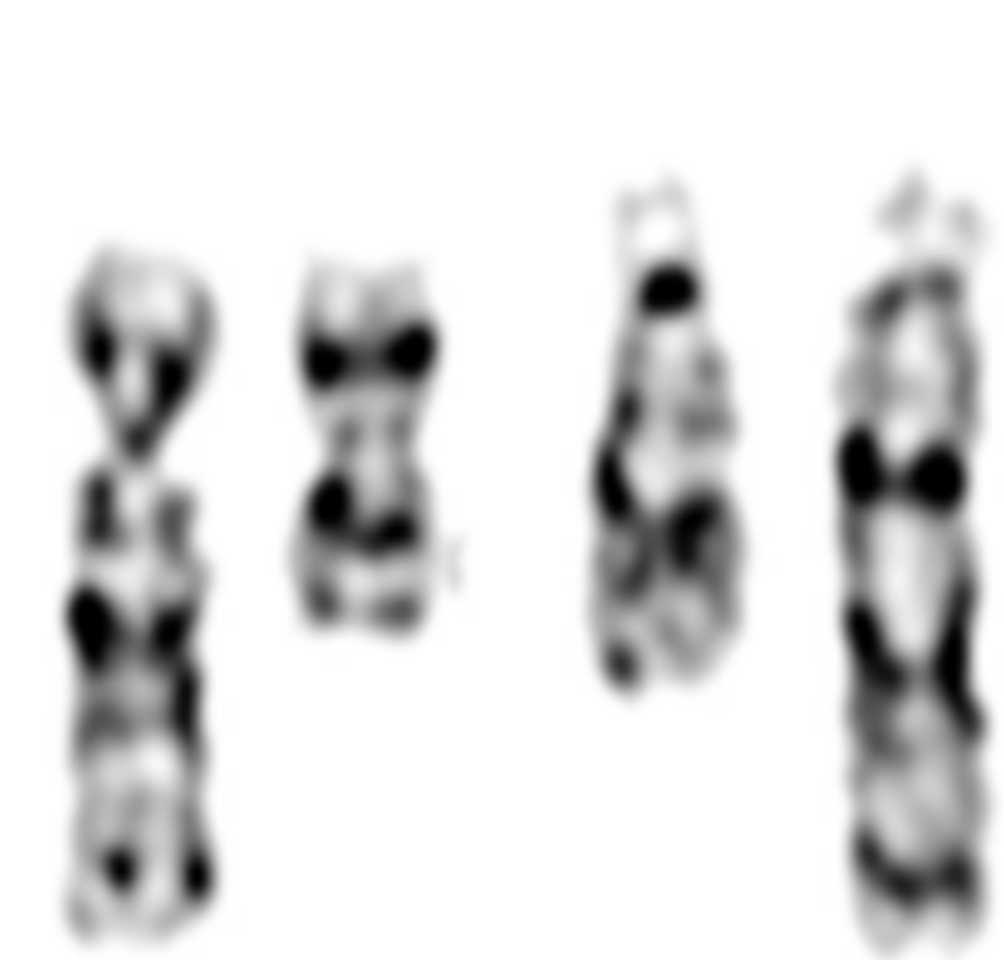

5 13  
t(5;13)(q11.2;q22)

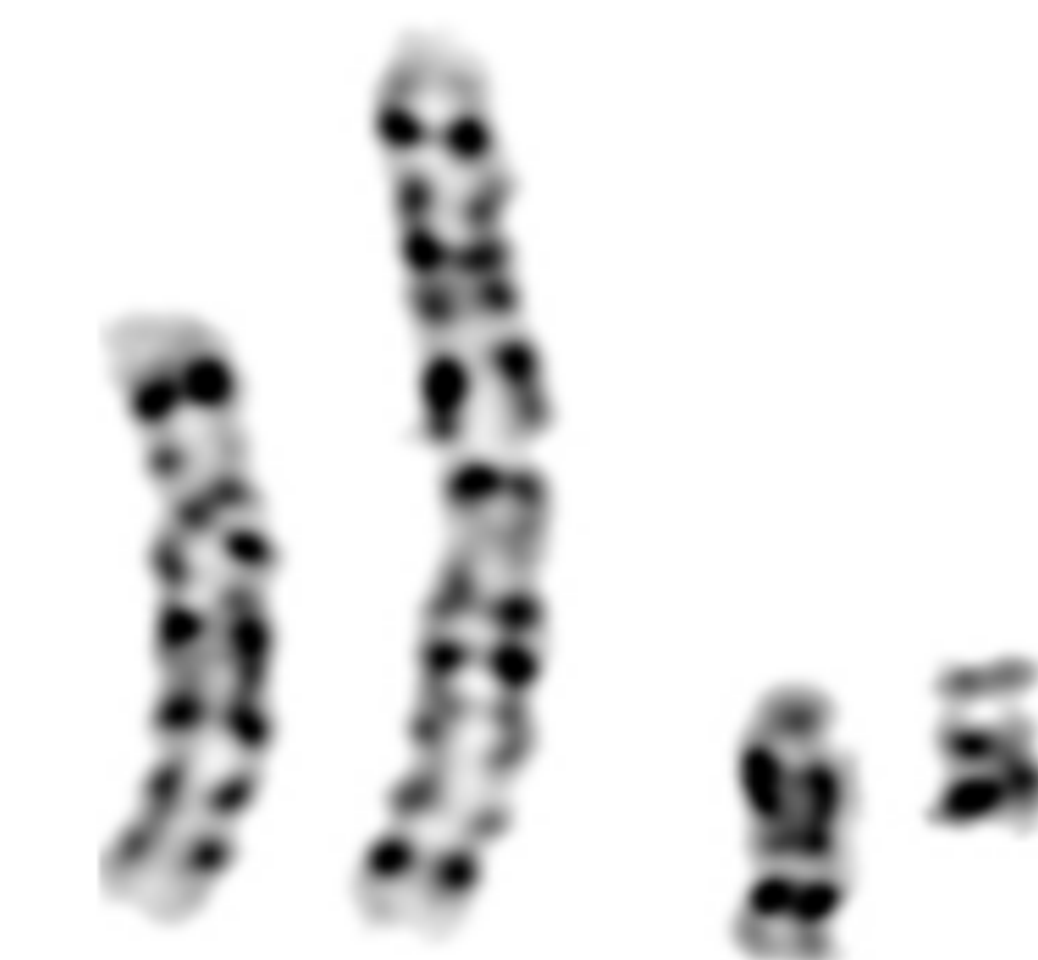

5 14  
t(5;14)(p15.1;q11.2)

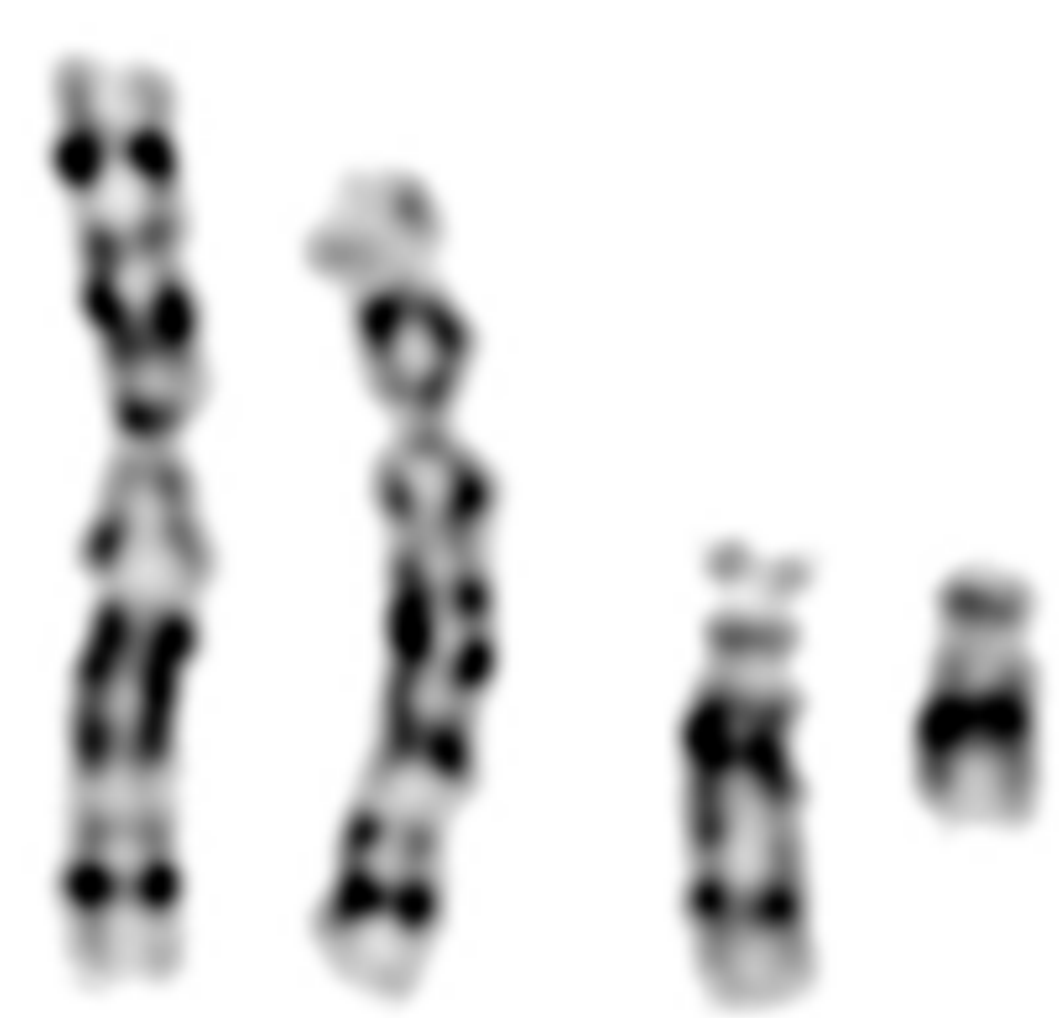

5 14  
t(5;14)(p15.3;q24)

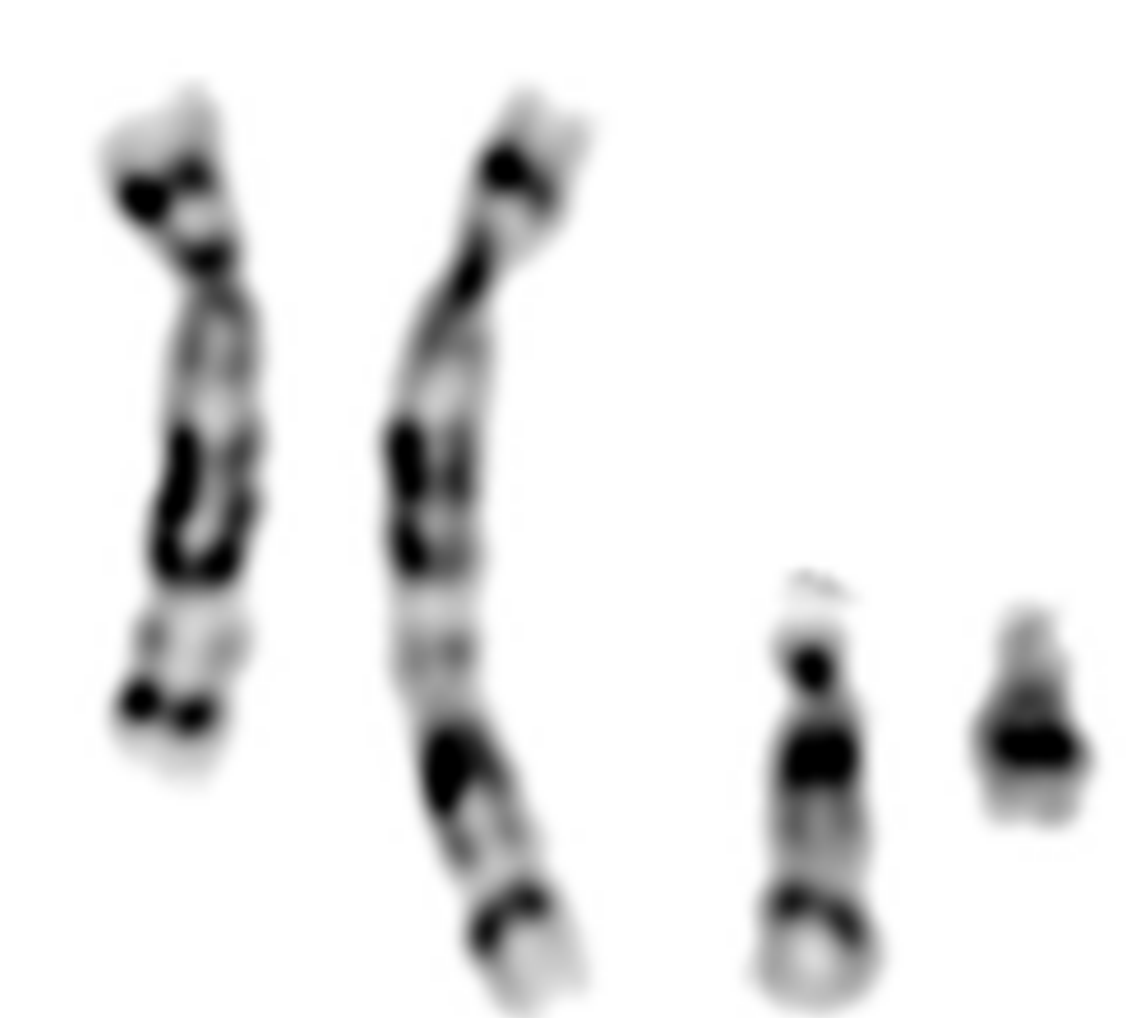

5 14  
t(5;14)(q33;q11.2)

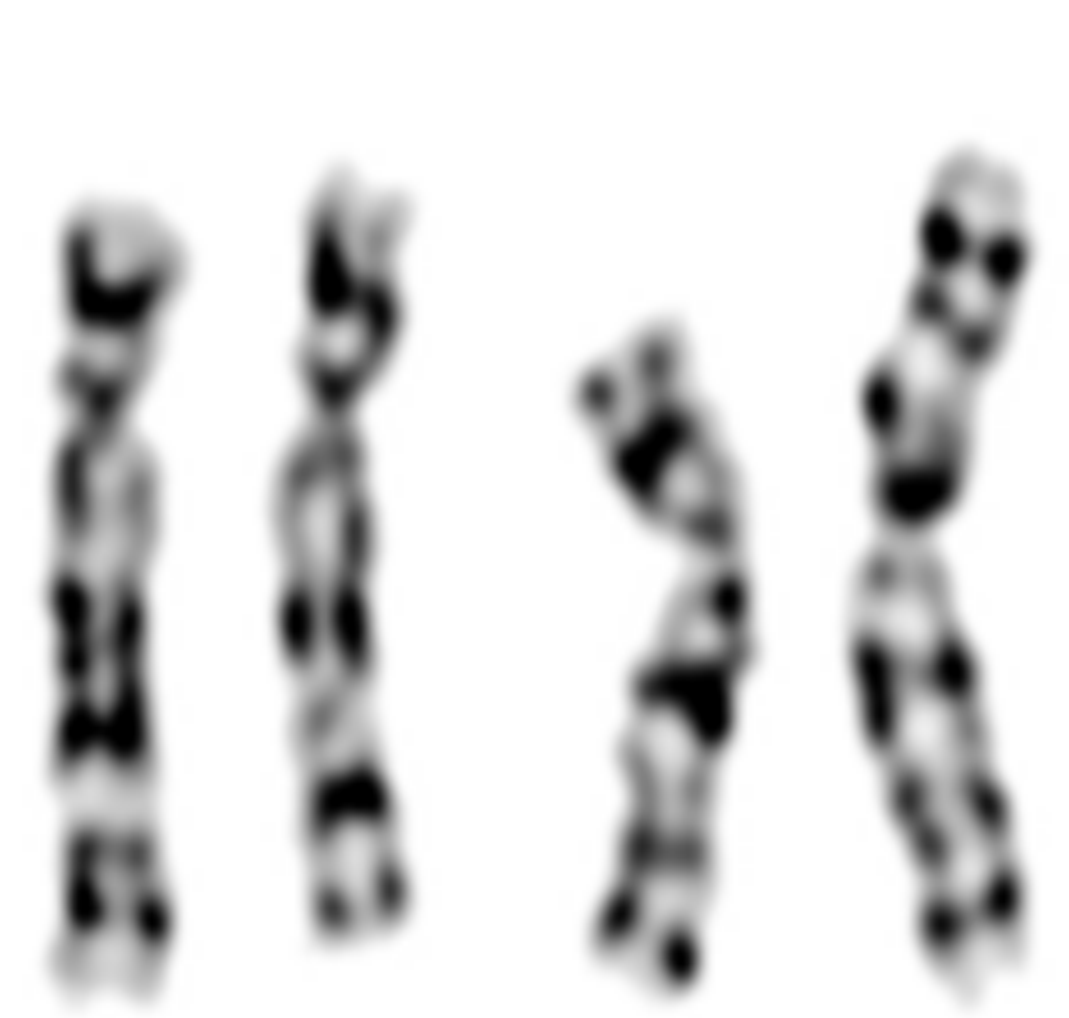

5 X  
t(X;5)(p11.2;q31)

Supplement: Supplementary file 6 — Supporting Information 6 FIGURE S5: Representative karyotype images of incidentally detected abnormalities involving chromosome 5. [file GENR-2026-4906805-s005.pdf]

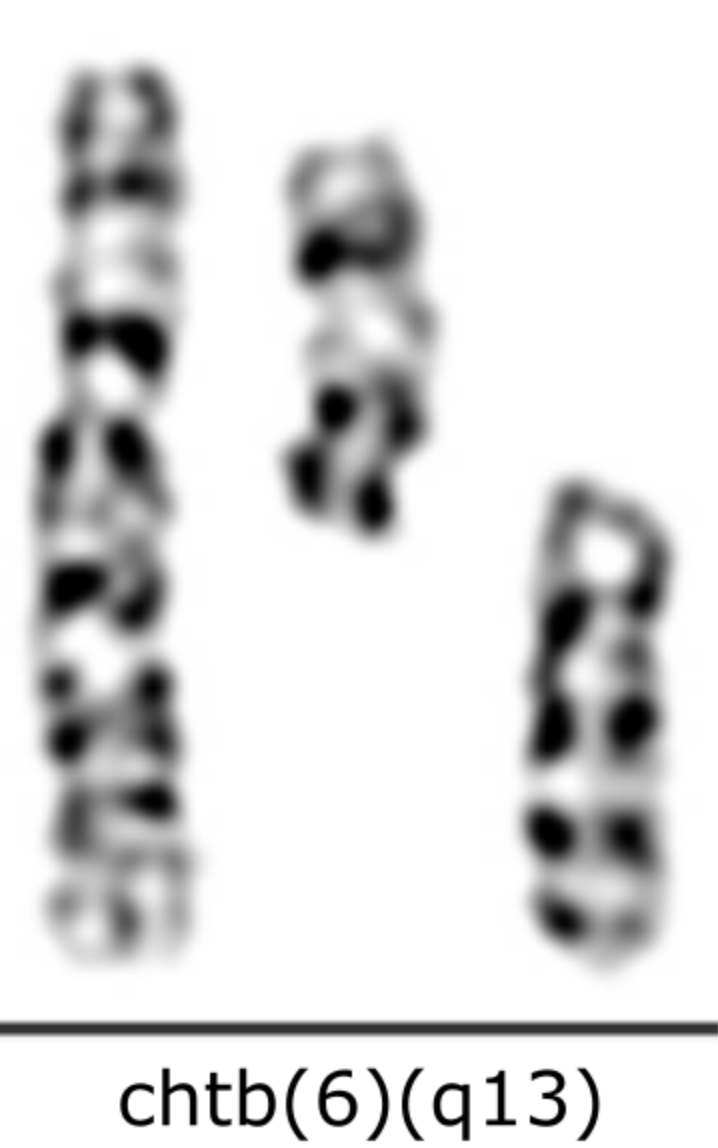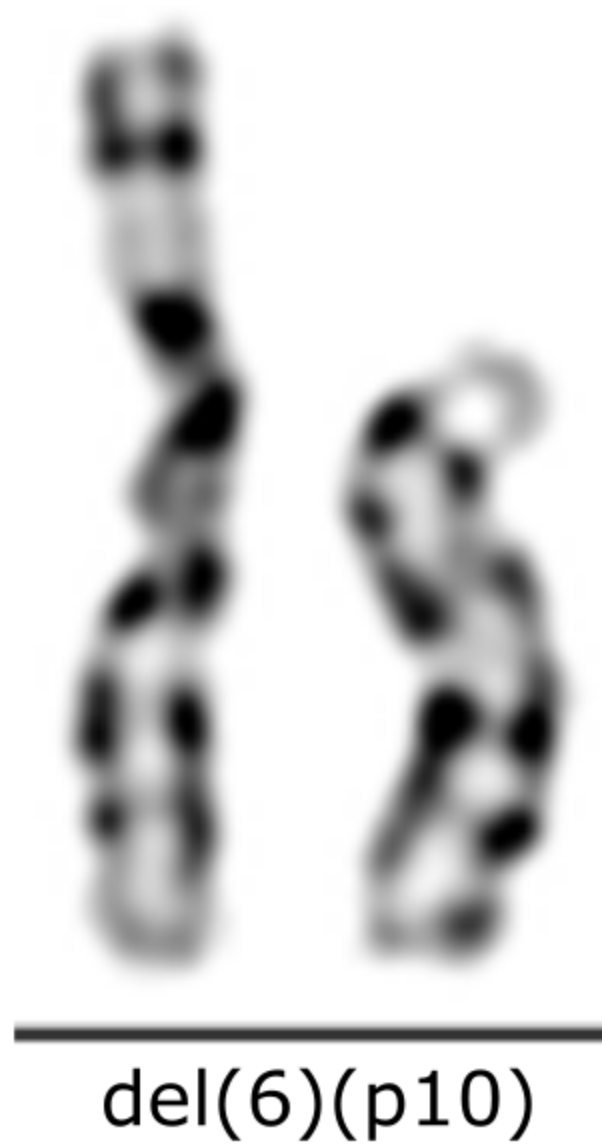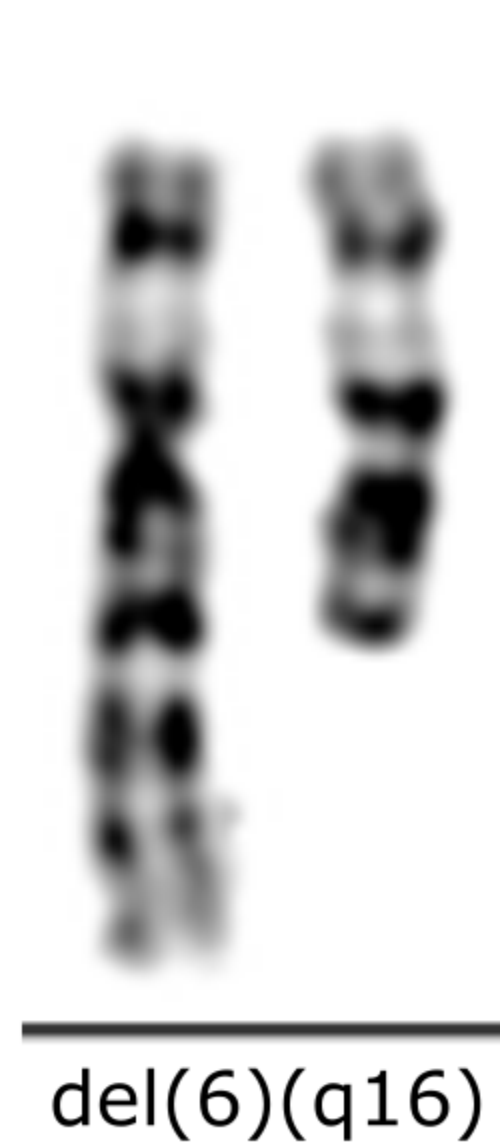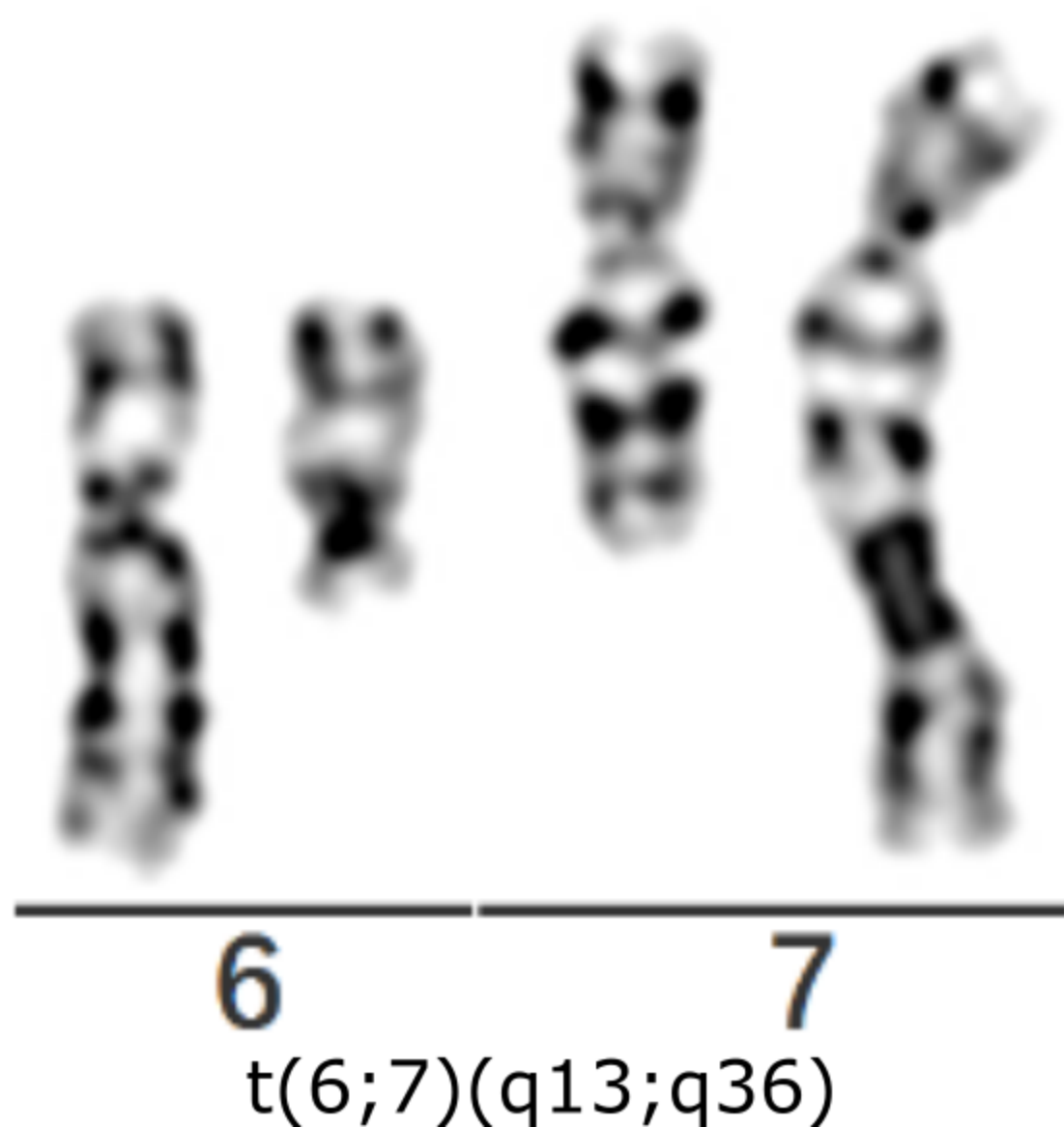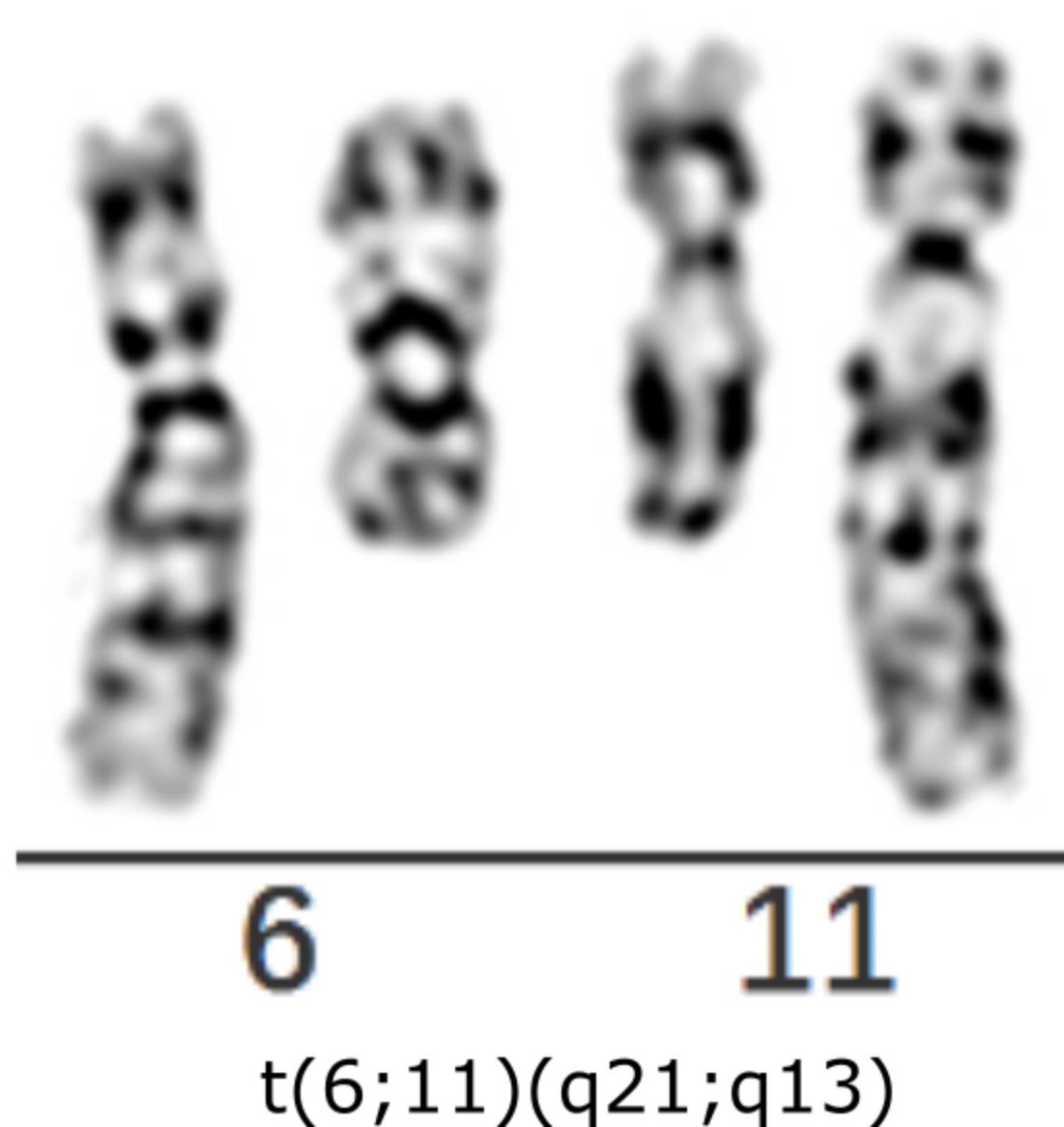

Supplement: Supplementary file 7 — Supporting Information 7 FIGURE S6: Representative karyotype images of incidentally detected abnormalities involving chromosome 6. [file GENR-2026-4906805-s006.pdf]

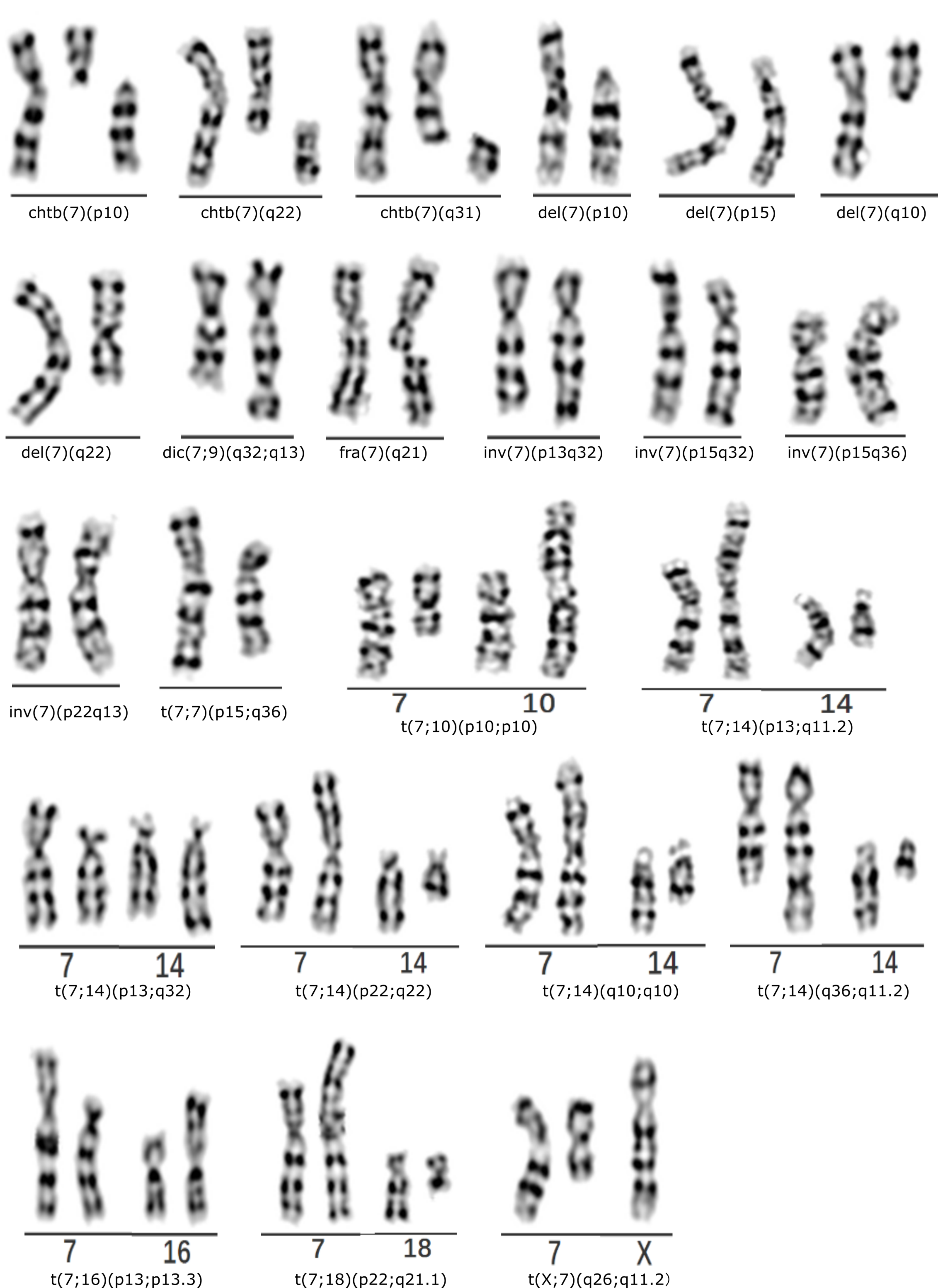

Supplement: Supplementary file 8 — Supporting Information 8 FIGURE S7: Representative karyotype images of incidentally detected abnormalities involving chromosome 7. [file GENR-2026-4906805-s007.pdf]

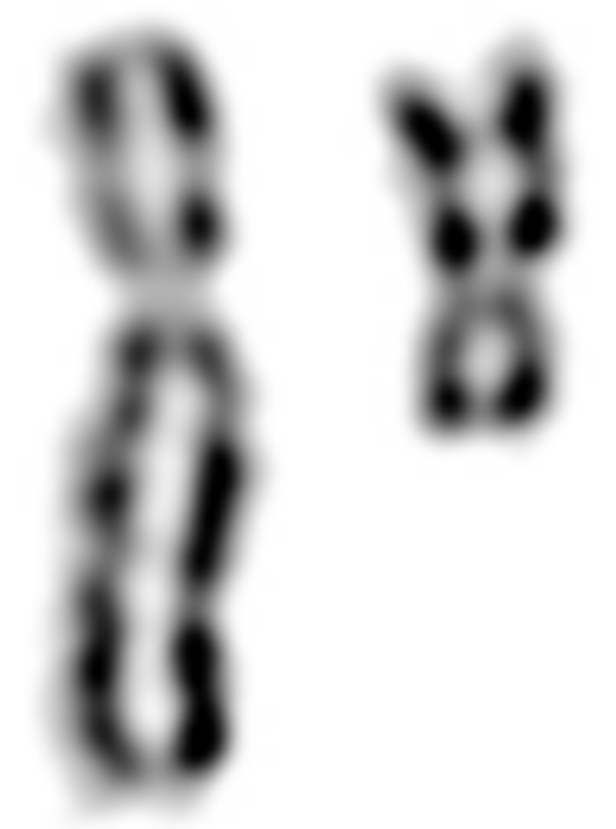

del(8)(q21.1)

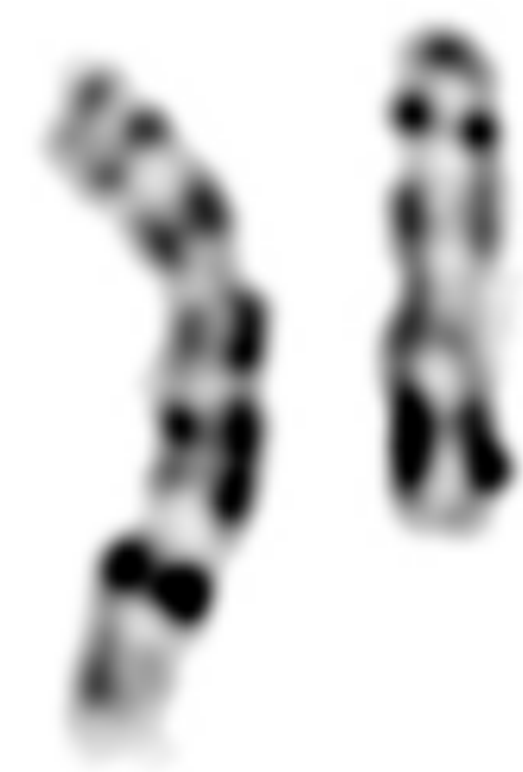

del(8)(q22)

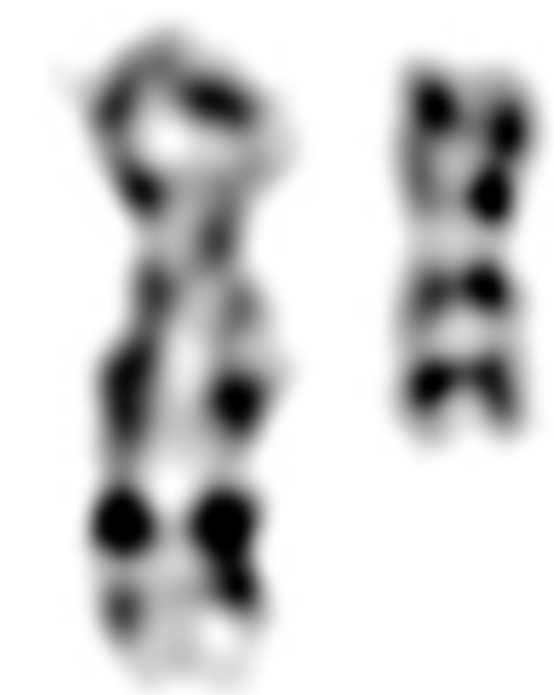

i(8)(p10)

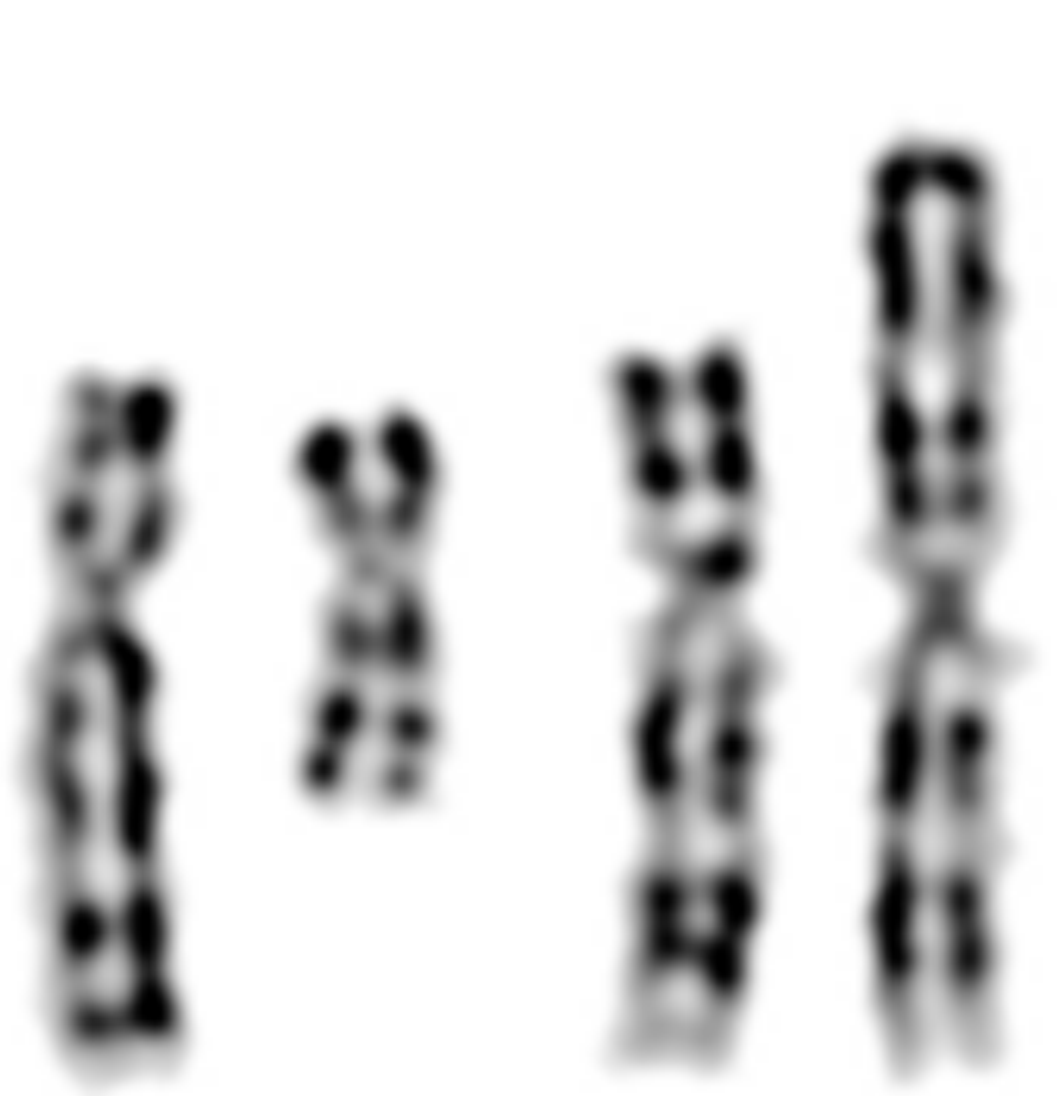

8

9

t(8;9)(p10;p10)

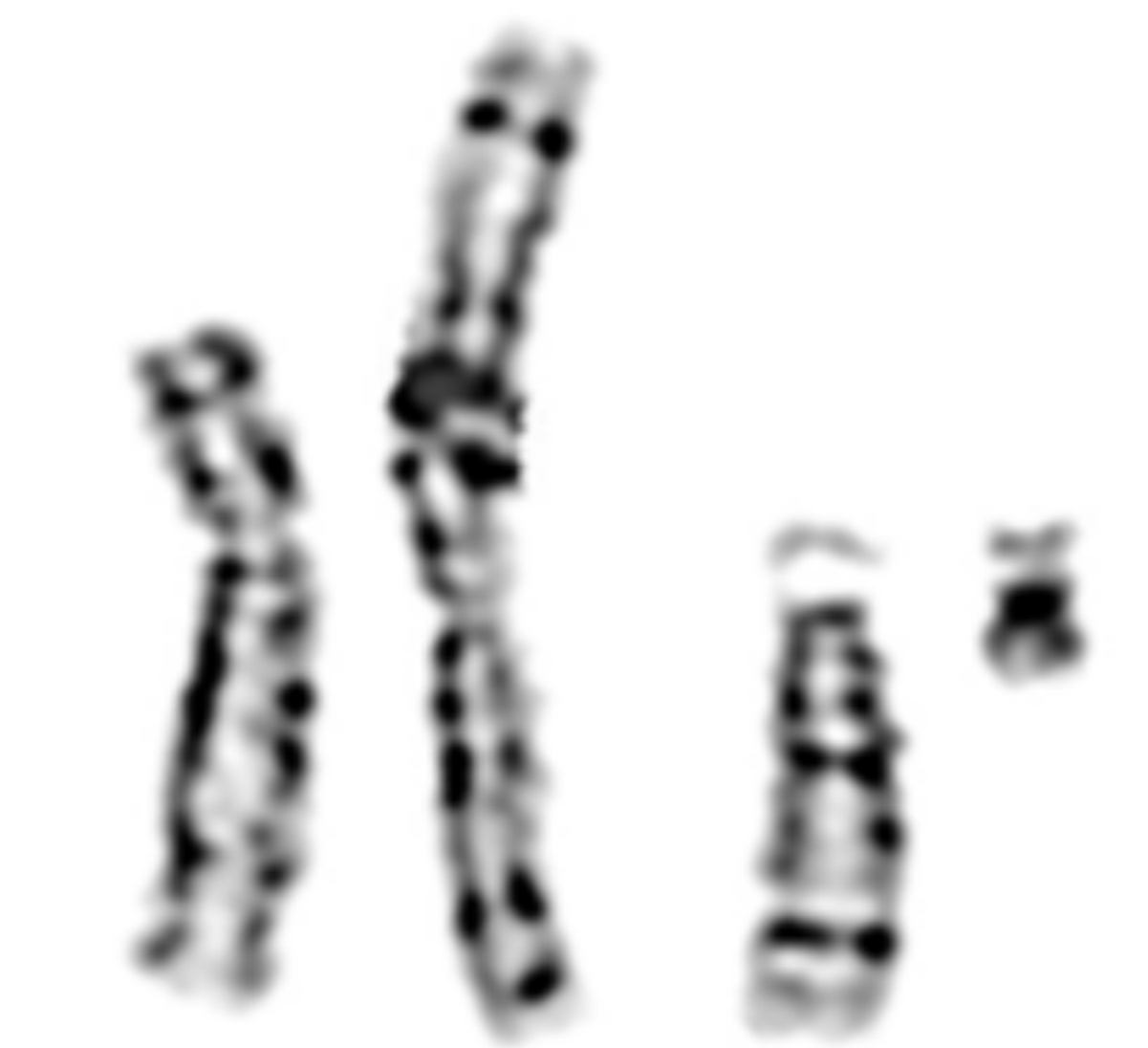

8

14

t(8;14)(p23;q11.2)

Supplement: Supplementary file 9 — Supporting Information 9 FIGURE S8: Representative karyotype images of incidentally detected abnormalities involving chromosome 8. [file GENR-2026-4906805-s008.pdf]

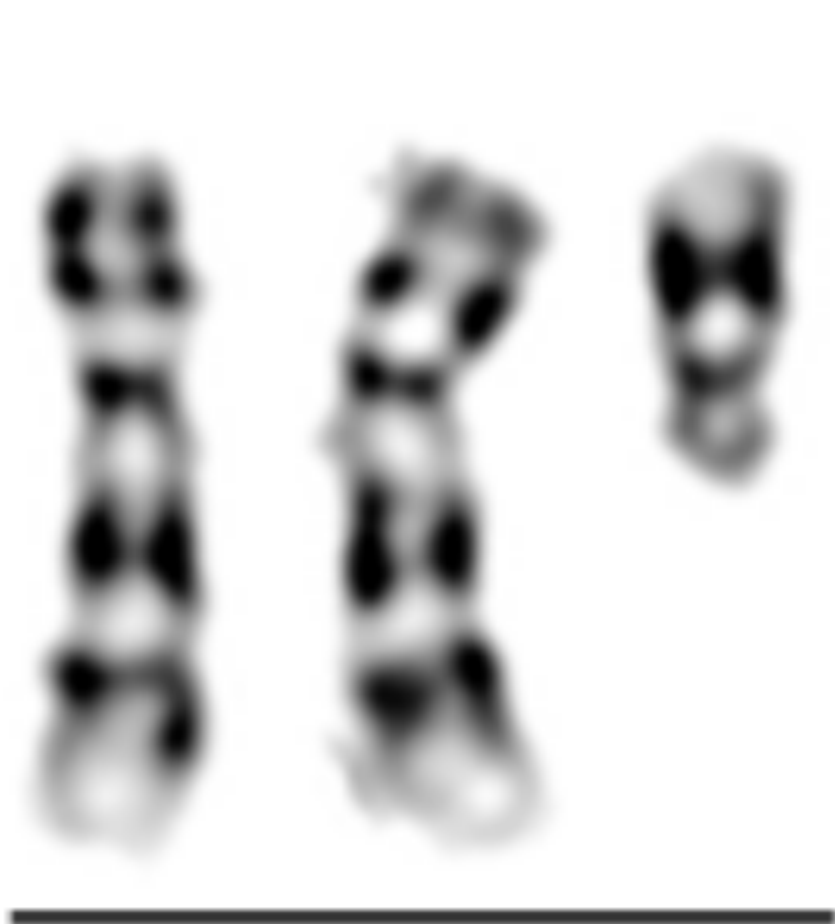

+der(9)del(9)(q13)

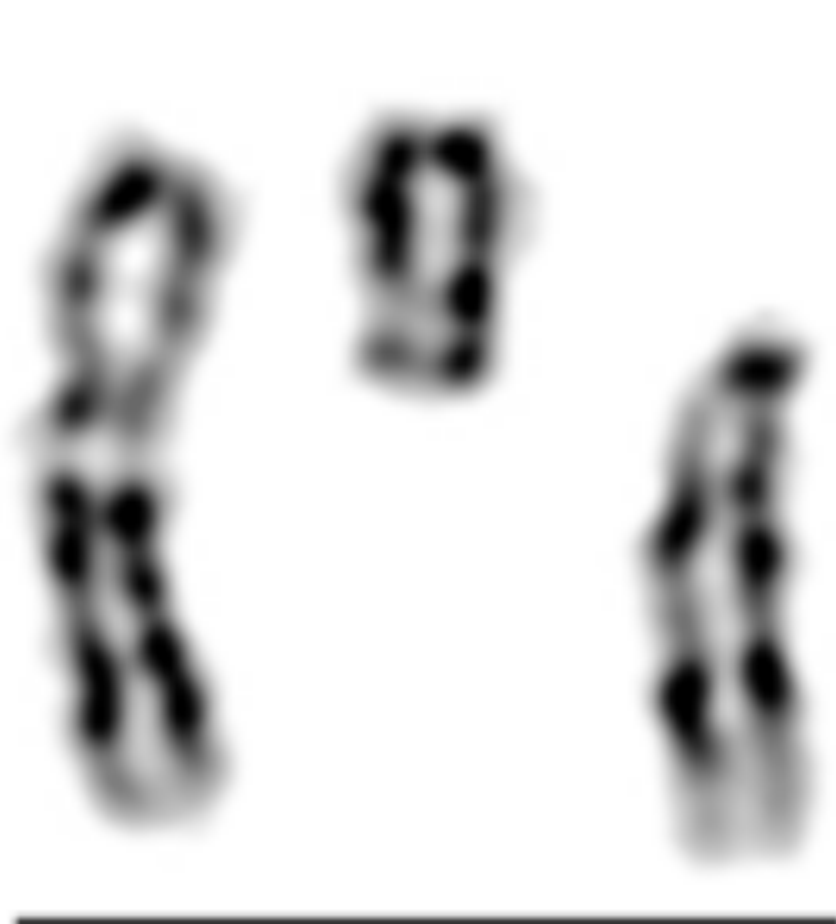

ctb(9)(q10)

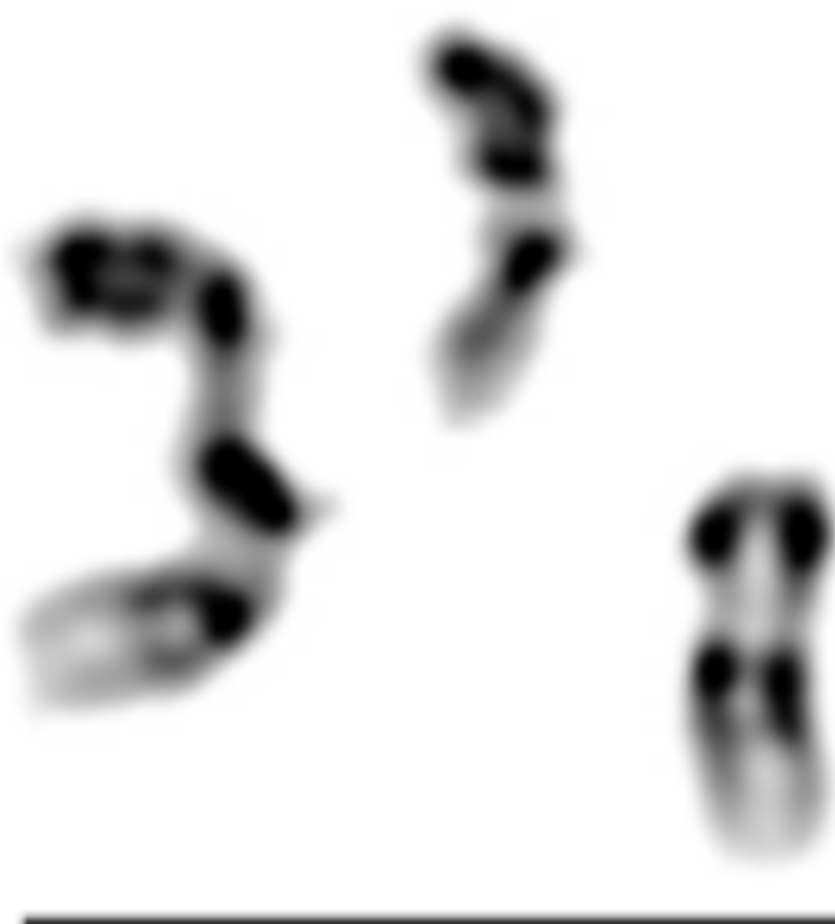

ctb(9)(q13)

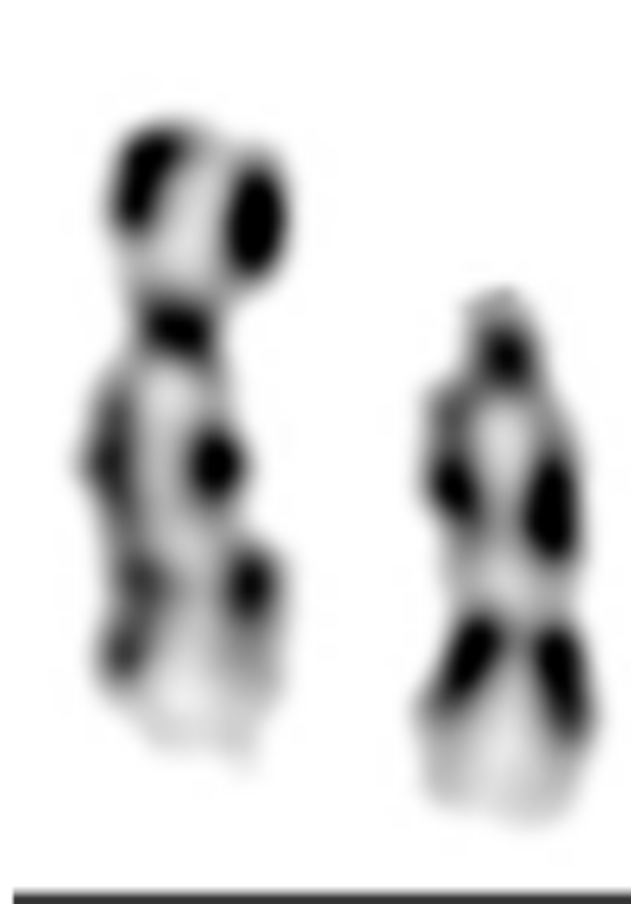

del(9)(p10)

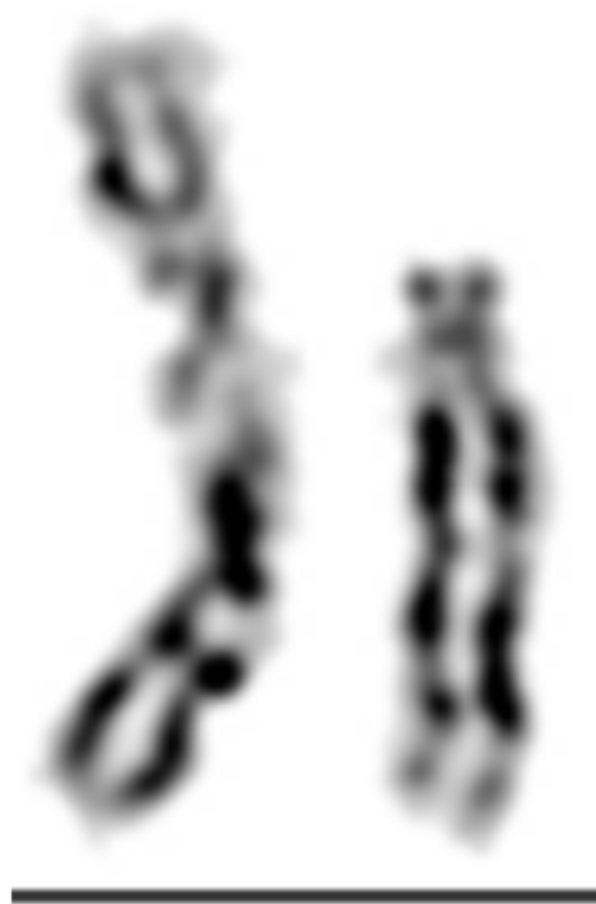

del(9)(p13)

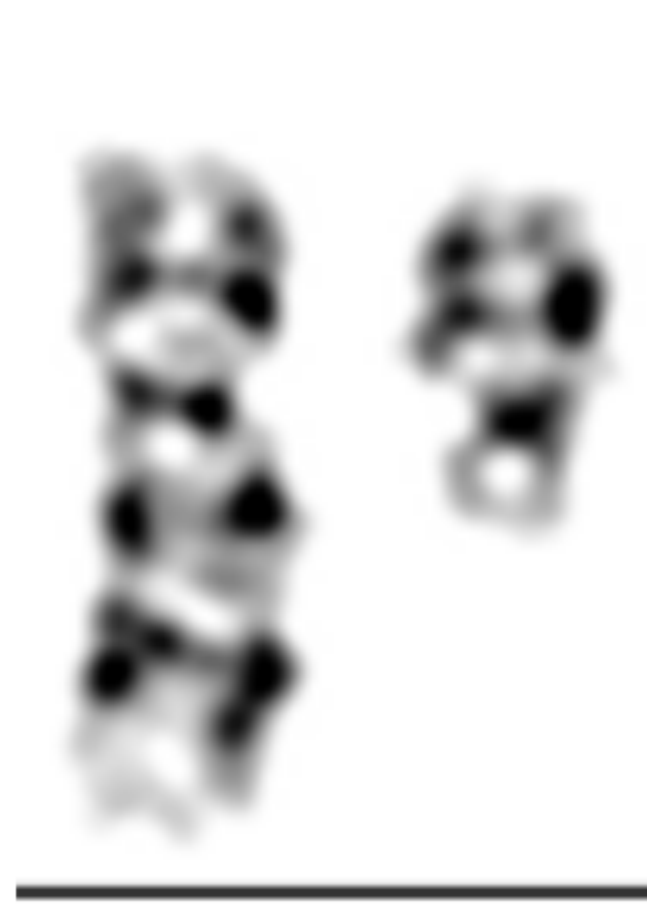

del(9)(q13)

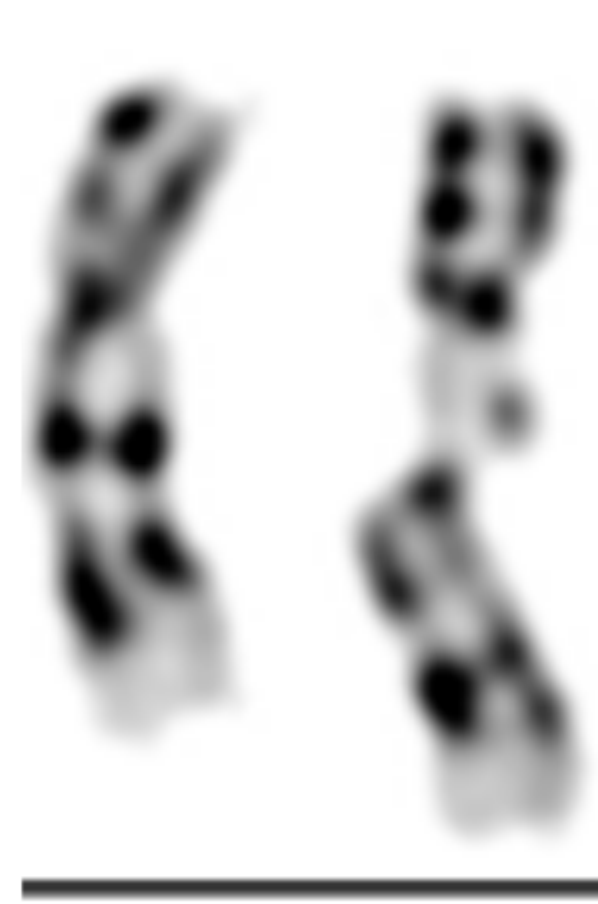

fra(9)(q13)

Supplement: Supplementary file 10 — Supporting Information 10 FIGURE S9: Representative karyotype images of incidentally detected abnormalities involving chromosome 9. [file GENR-2026-4906805-s009.pdf]

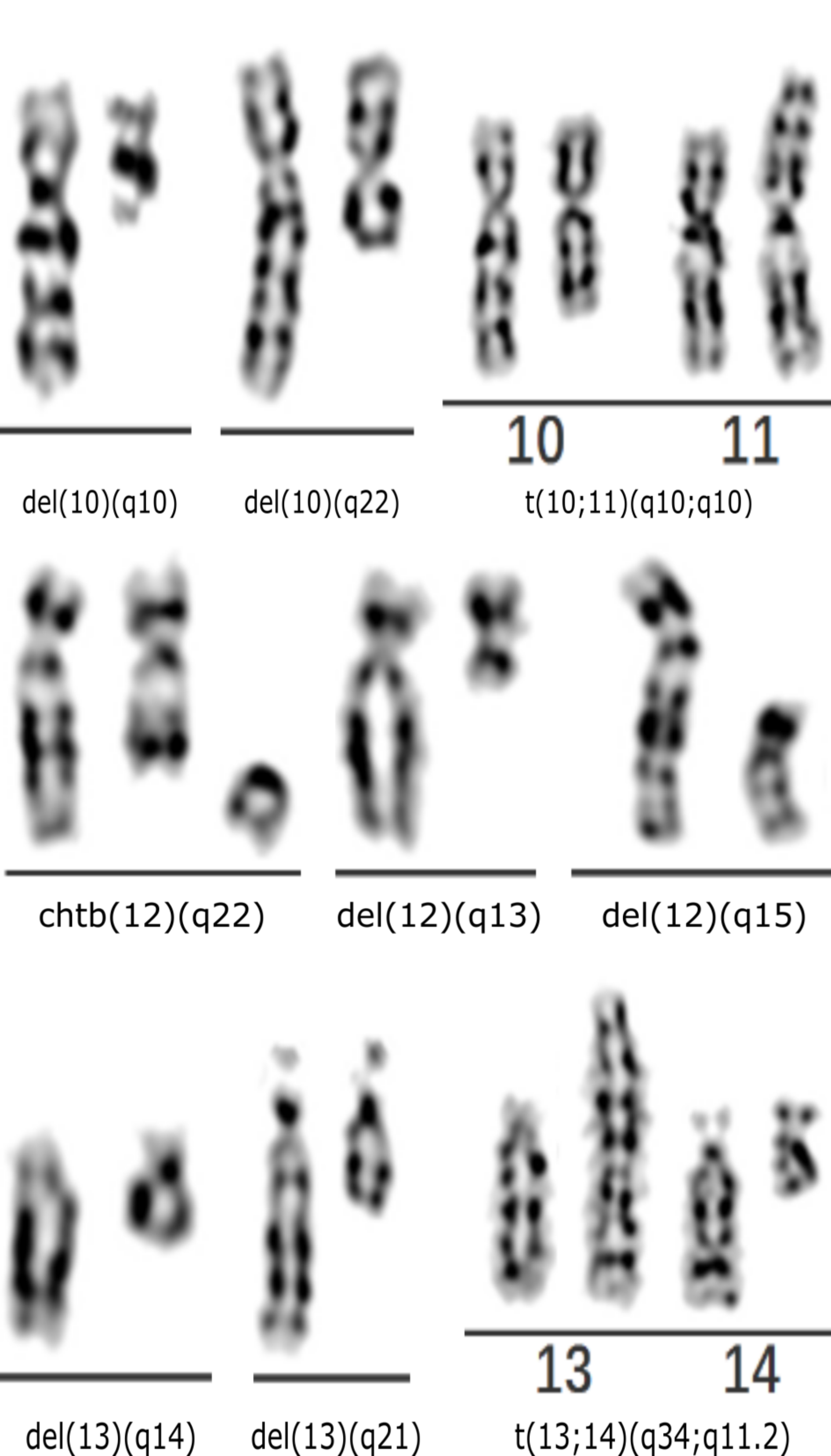

Supplement: Supplementary file 11 — Supporting Information 11 FIGURE S10: Representative karyotype images of incidentally detected abnormalities involving chromosomes 10, 12, and 13. [file GENR-2026-4906805-s010.pdf]

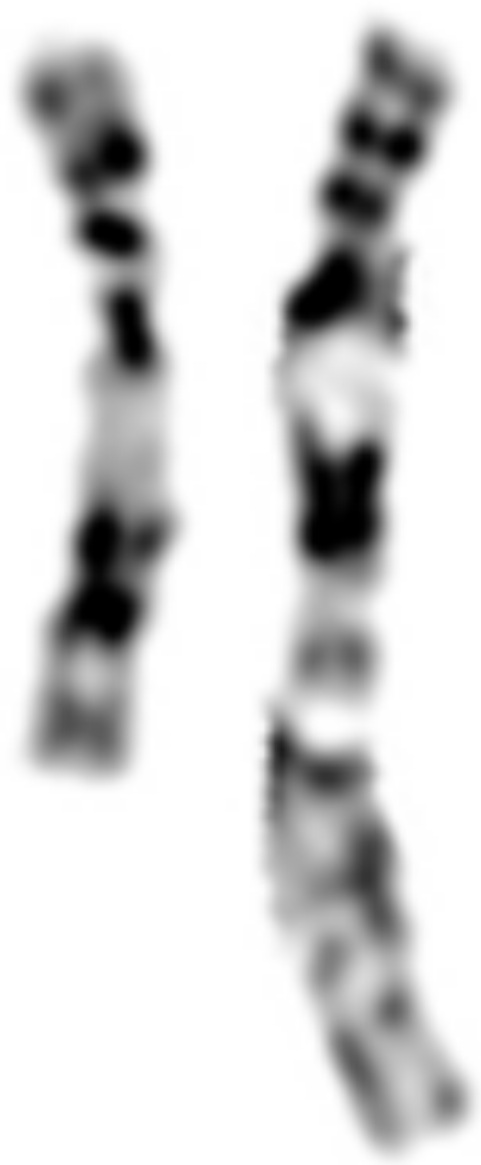

add(11)(q25)

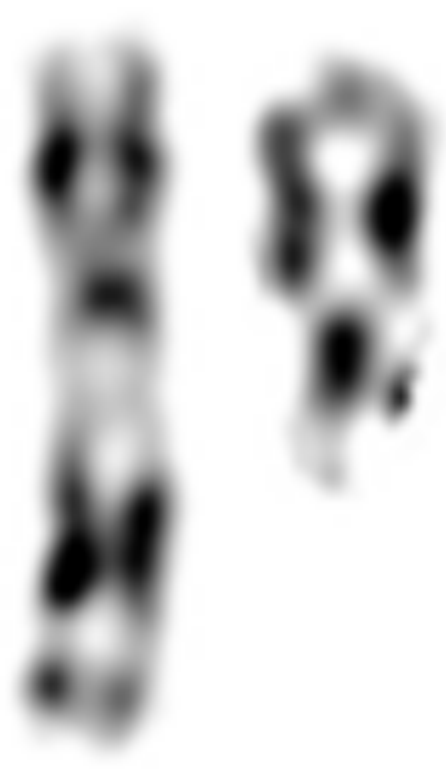

del(11)(q10)

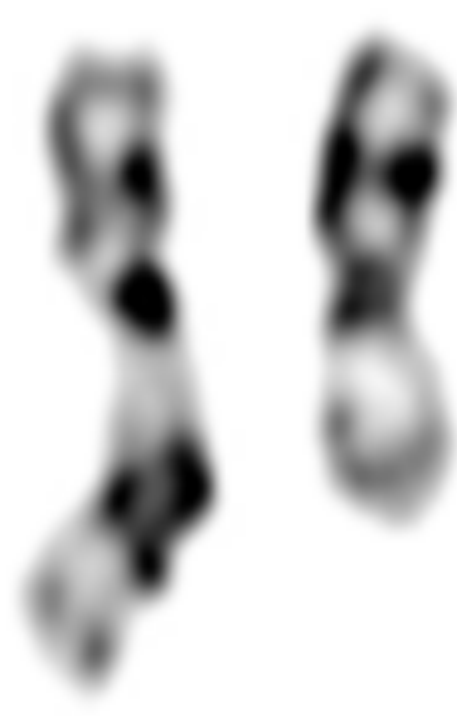

del(11)(q14)

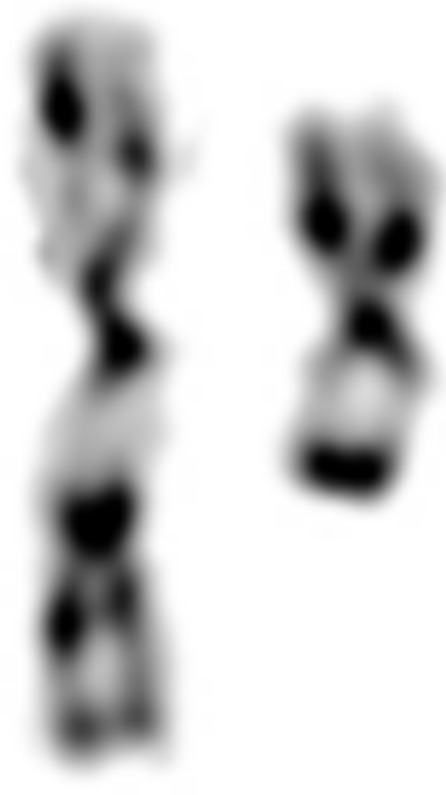

del(11)(q21)

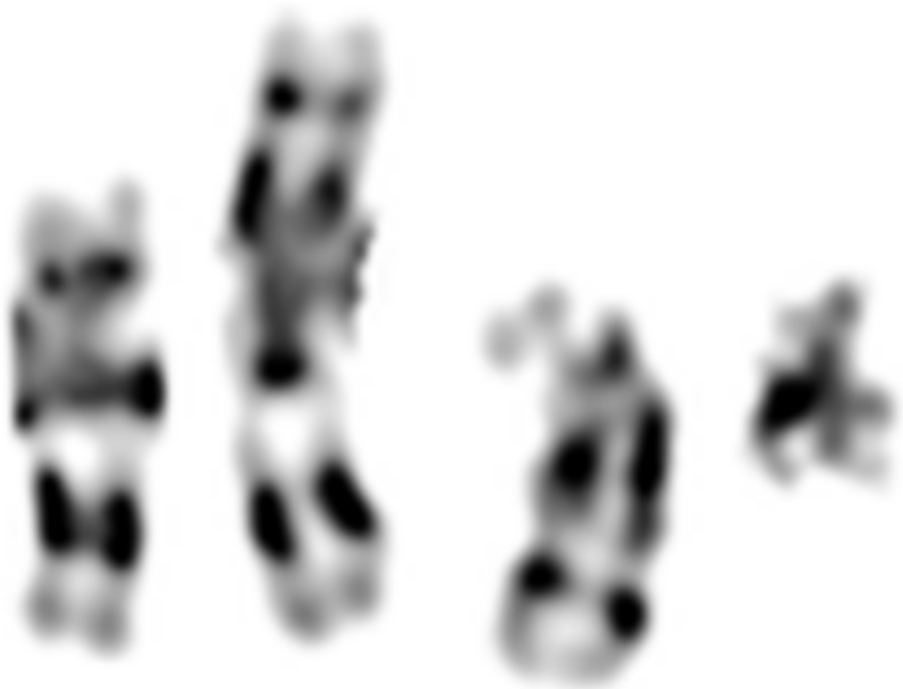

11

14

t(11;14)(p11.2;q11.2)

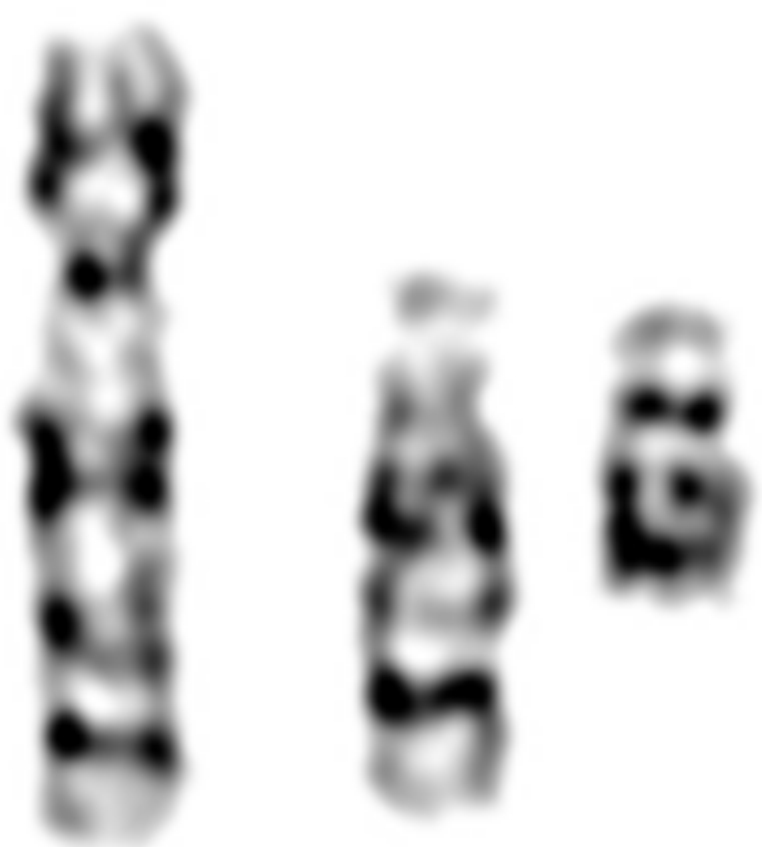

11

14

t(11;14)(q25;q22)

Supplement: Supplementary file 12 — Supporting Information 12 FIGURE S11: Representative karyotype images of incidentally detected abnormalities involving chromosome 11. [file GENR-2026-4906805-s011.pdf]

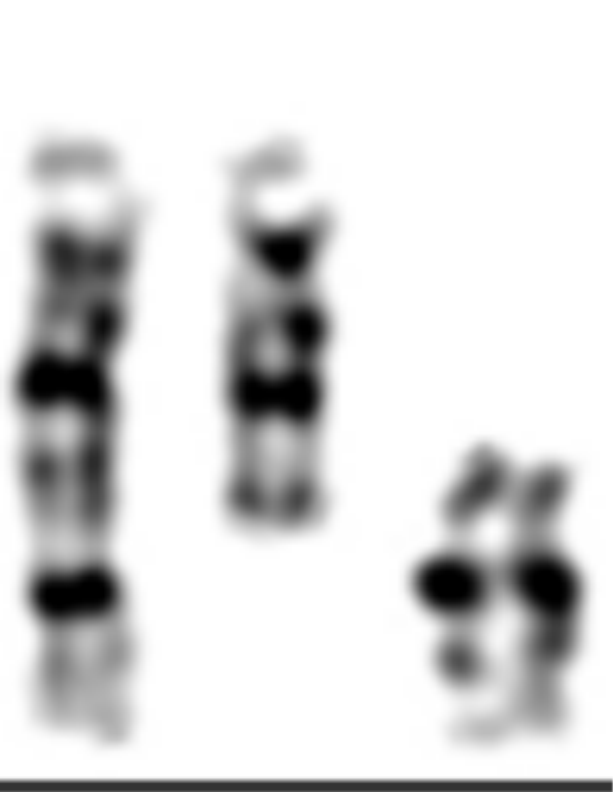

ch**tb**(14)(q24)

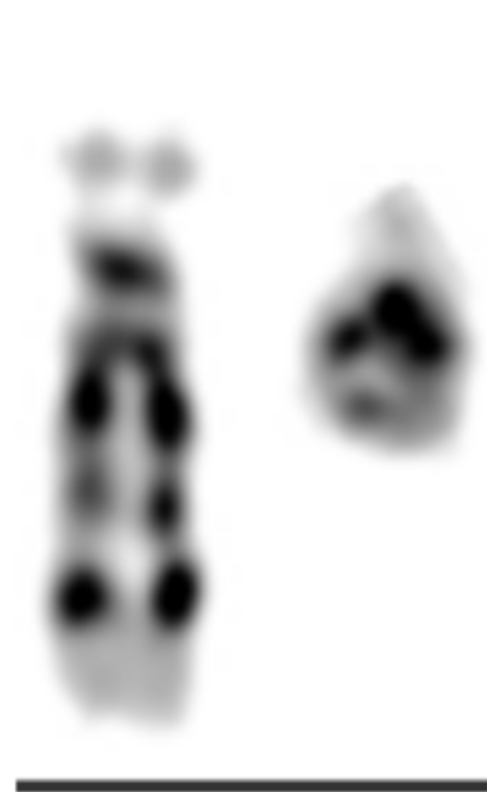

del(14)(q13)

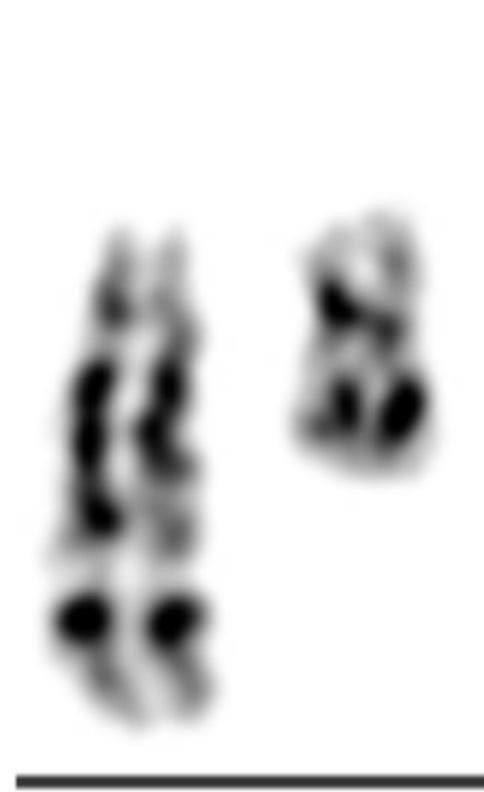

del(14)(q21)

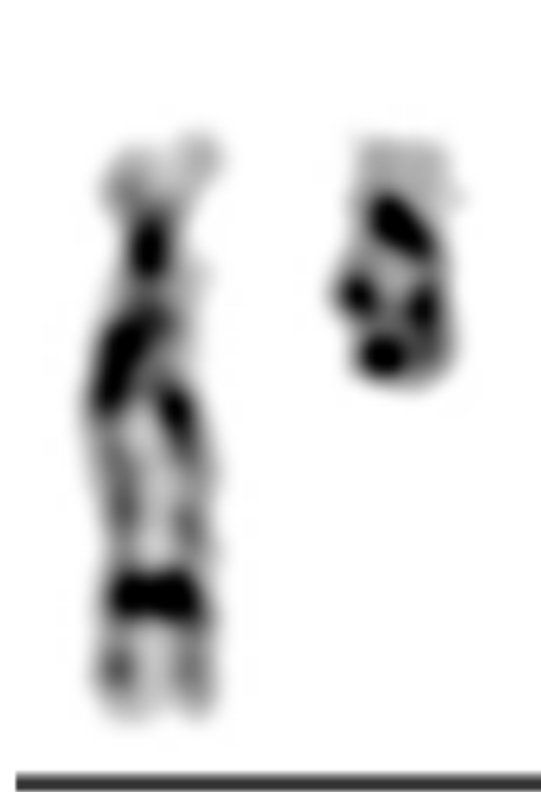

del(14)(q22)

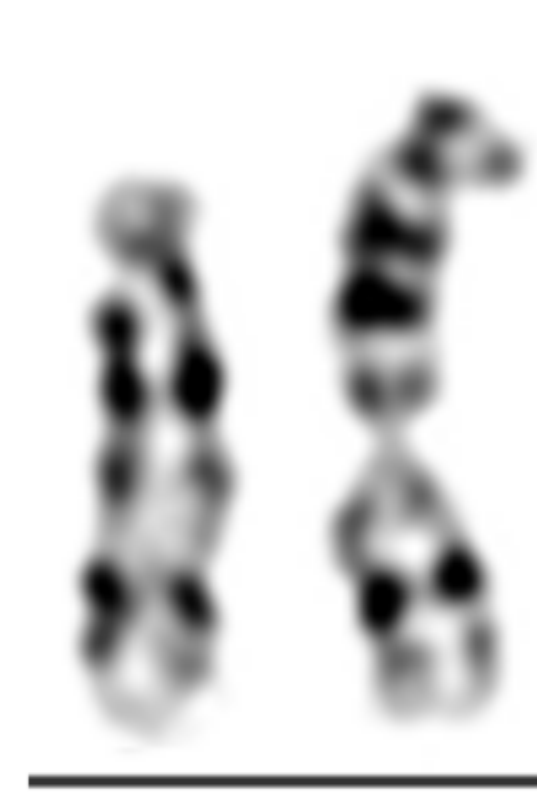

fra(14)(q24.1)

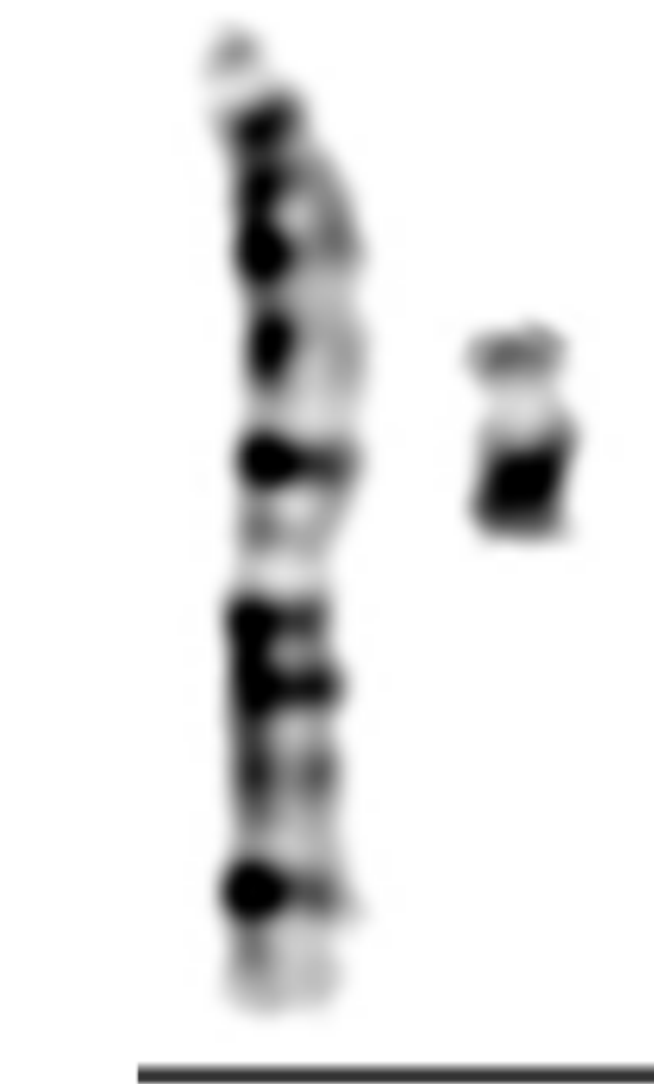

t(14;14)(q11.2;q32)

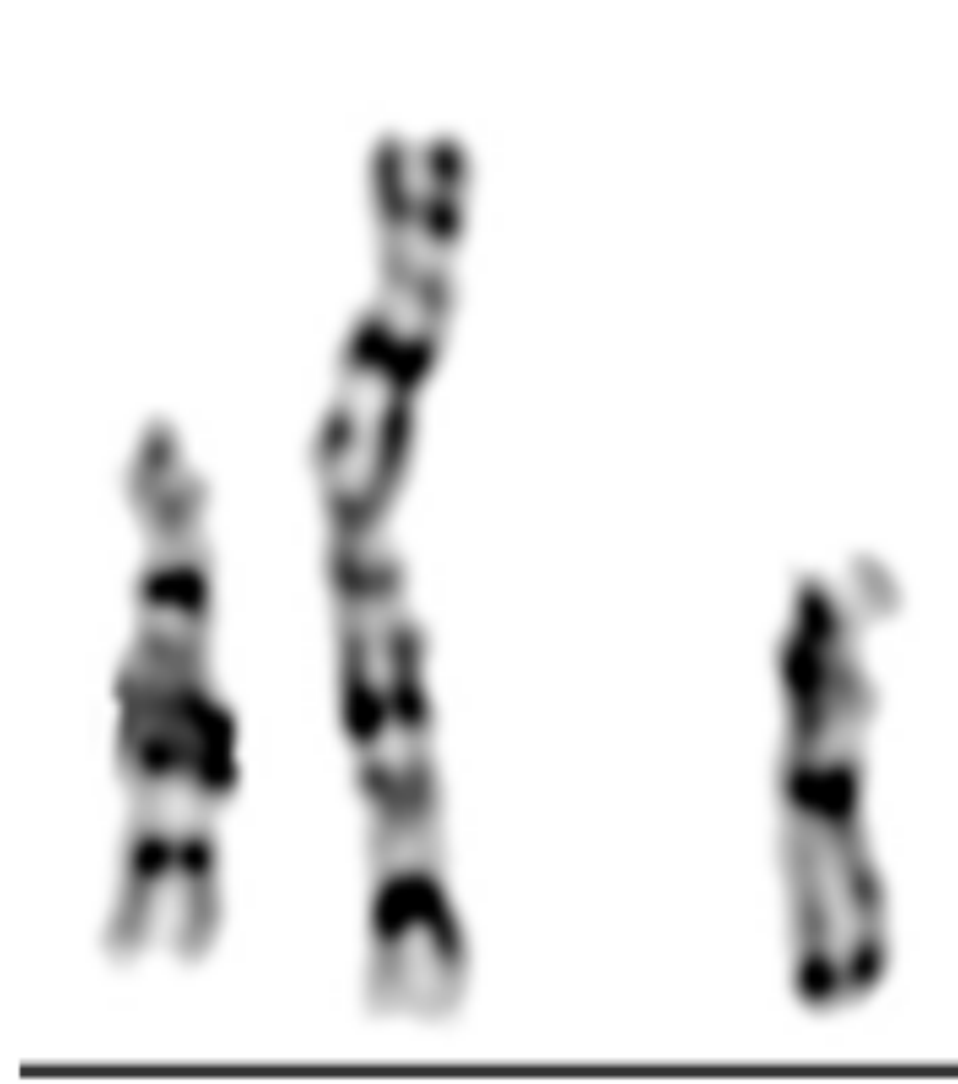

14 15  
rob(14;15)(q10;q10)

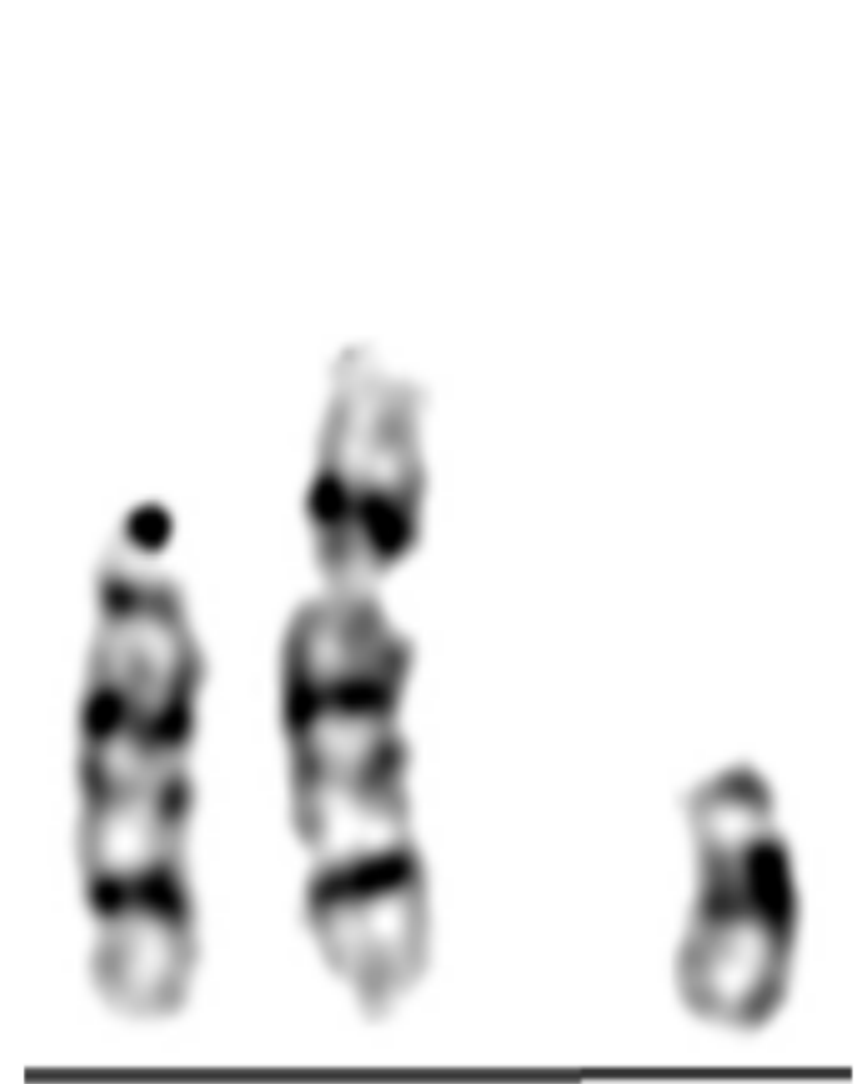

14 21  
rob(14;21)(q10;q10)

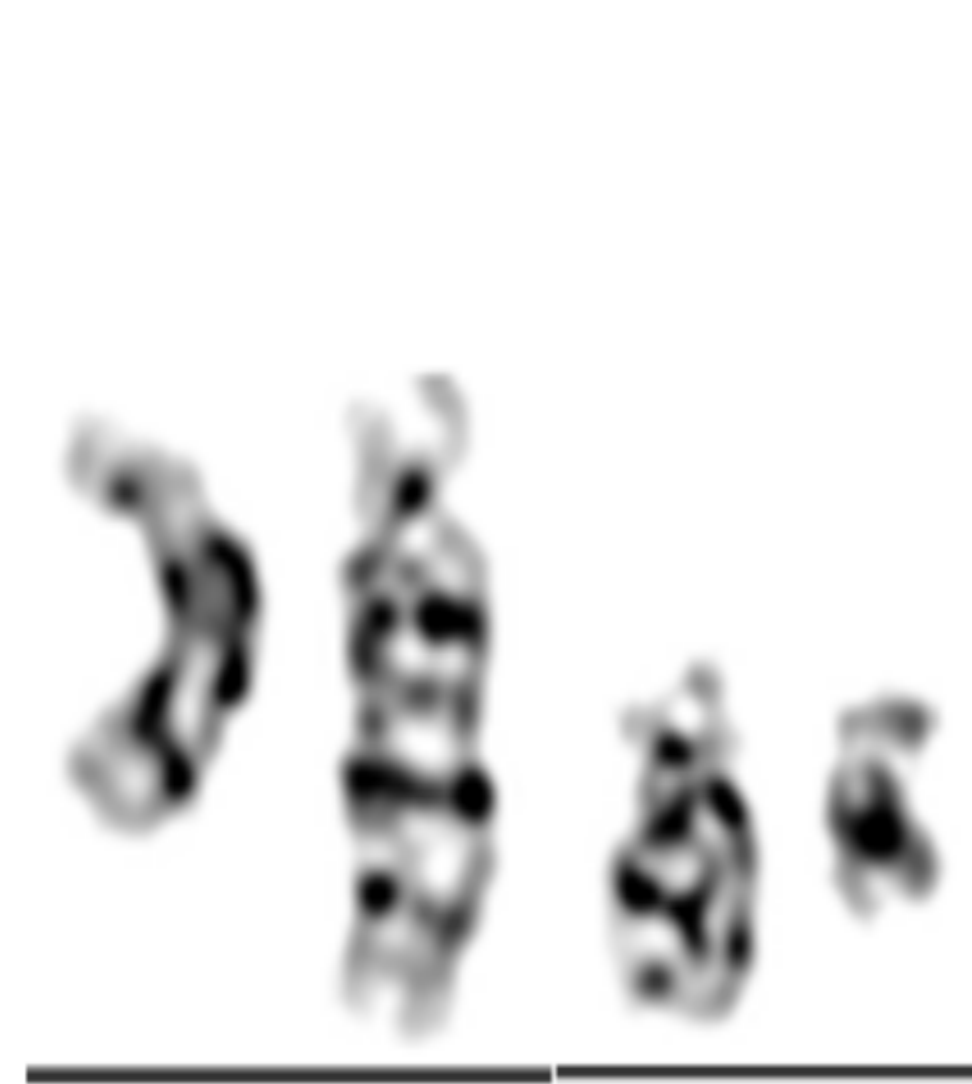

14 22  
t(14;22)(q32;q11.2)

Supplement: Supplementary file 13 — Supporting Information 13 FIGURE S12: Representative karyotype images of incidentally detected abnormalities involving chromosome 14. [file GENR-2026-4906805-s012.pdf]

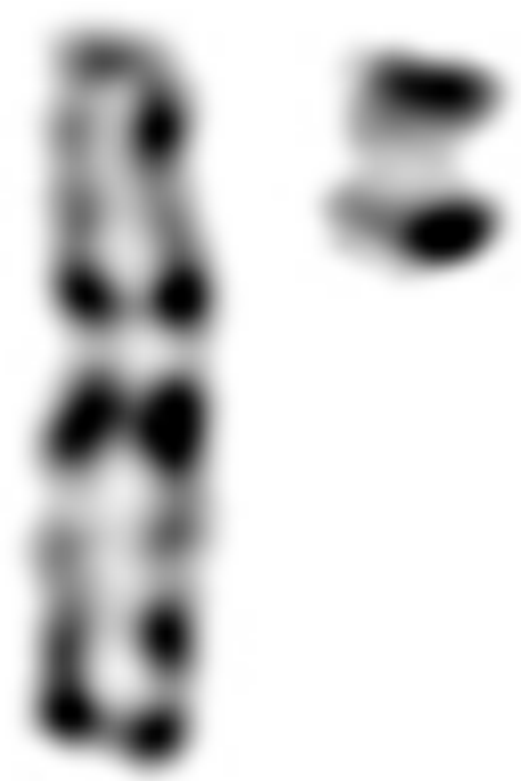

del(15)(q15)

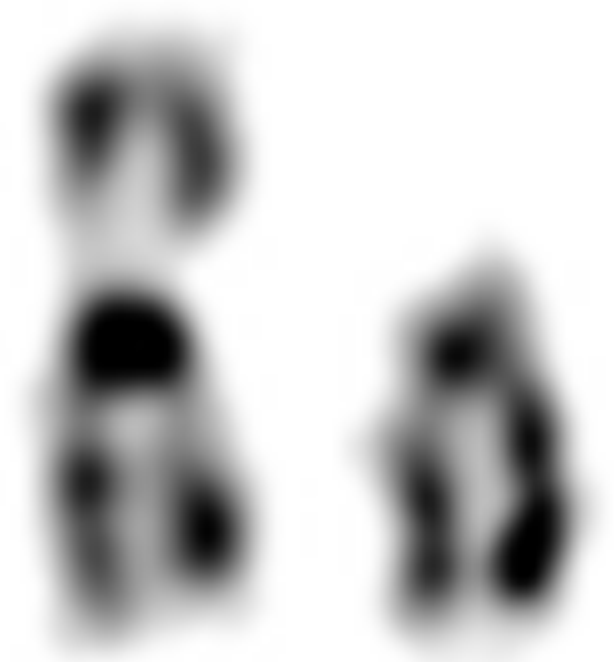

del(16)(p10)

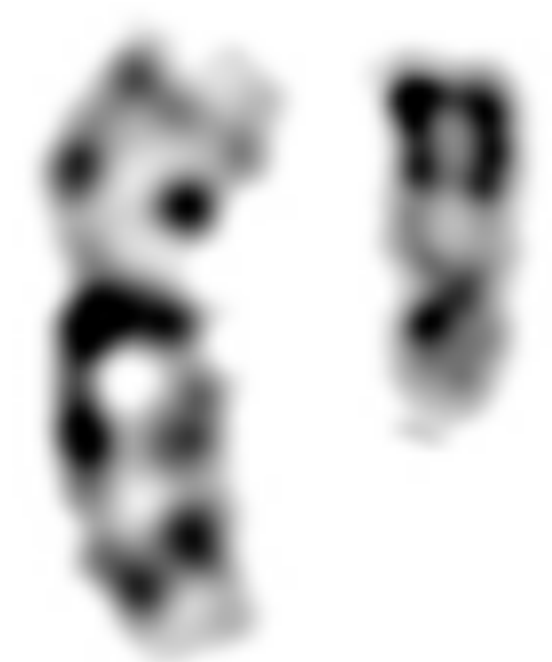

del(16)(q12)

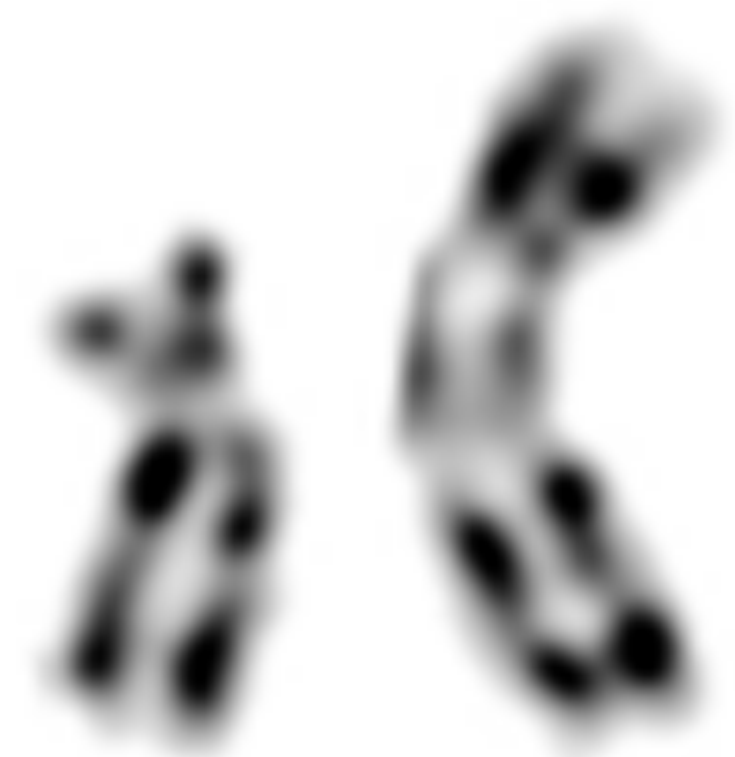

add(18)

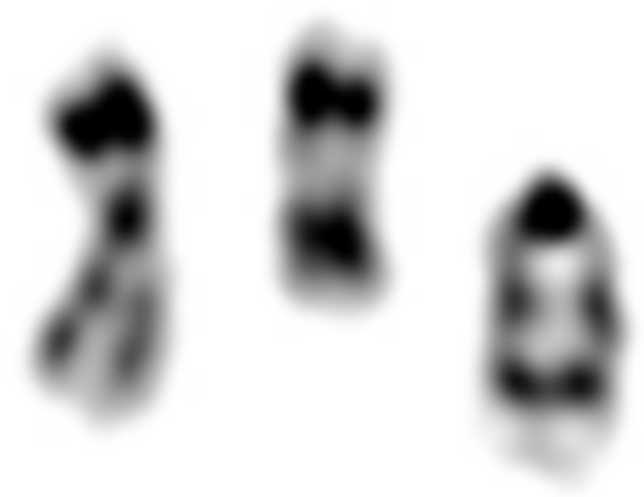

chtb(20)(q10)

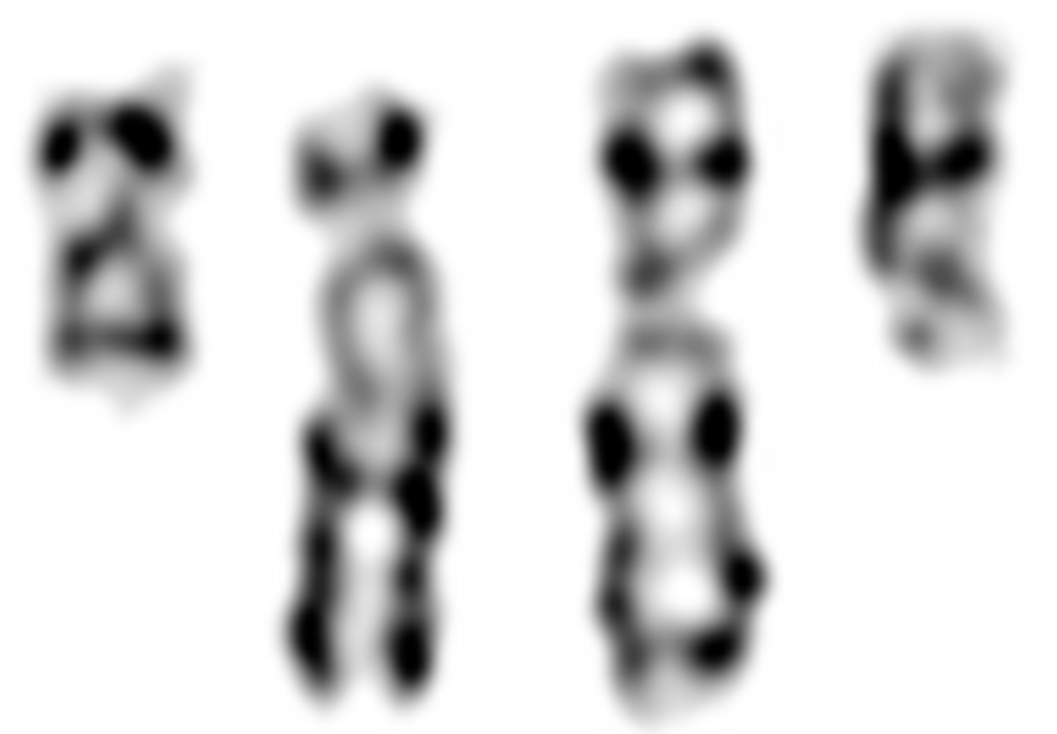

20 X  
t(X;20)(q13;q13.3)

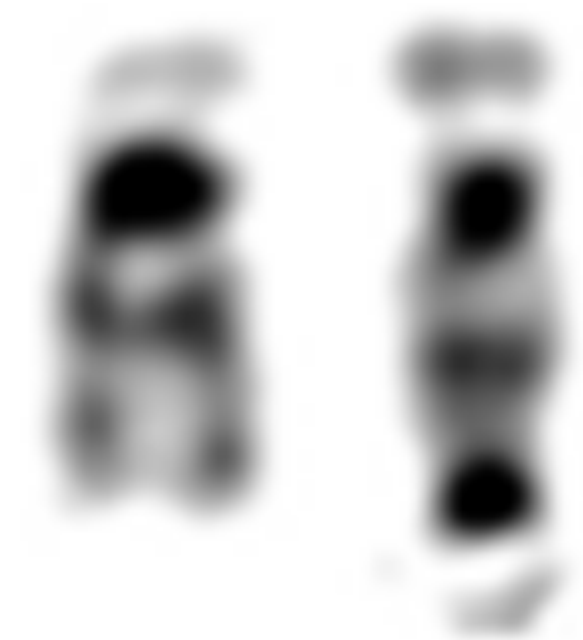

idic(22)(q13)

Supplement: Supplementary file 14 — Supporting Information 14 FIGURE S13: Representative karyotype images of incidentally detected abnormalities involving chromosomes 15, 16, 18, 20, and 22. [file GENR-2026-4906805-s013.pdf]
